# An Air‐Stable Heterobimetallic Si_2_M_2_ Tetrahedral Cluster

**DOI:** 10.1002/anie.201916116

**Published:** 2020-02-20

**Authors:** Gizem Dübek, Franziska Hanusch, Dominik Munz, Shigeyoshi Inoue

**Affiliations:** ^1^ Department of Chemistry Catalysis Research Center and Institute of Silicon Chemistry Technical University Munich Lichtenbergstraße 4 85748 Garching bei München Germany; ^2^ Friedrich-Alexander-Universität Erlangen-Nürnberg (FAU) Department of Chemistry and Pharmacy General and Inorganic Chemistry Egerlandstraße 1 91058 Erlangen Germany

**Keywords:** cluster compounds, silicon, silylidenes, silylidynes, tetrahedranes

## Abstract

Air‐ and moisture‐stable heterobimetallic tetrahedral clusters [Cp(CO)_2_MSiR]_2_ (M=Mo or W; R=Si*t*Bu_3_) were isolated from the reaction of N‐heterocyclic carbene (NHC) stabilized silyl(silylidene) metal complexes Cp(CO)_2_M=Si(Si*t*Bu_3_)NHC with a mild Lewis acid (BPh_3_). Alternatively, treatment of the NHC‐stabilized silylidene complex Cp(CO)_2_W=Si(Si*t*Bu_3_)NHC with stronger Lewis acids such as AlCl_3_ or B(C_6_F_5_)_3_ resulted in the reversible coordination of the Lewis acid to one of the carbonyl ligands. Computational investigations revealed that the dimerization of the intermediate metal silylidyne (M≡Si) complex to a tetrahedral cluster instead of a planar four‐membered ring is due to steric bulk.

## Graphical abstract

**Heterobimetallic** tetrahedrane‐type clusters with a Si_2_M_2_ (M=Mo, W) core were isolated as air‐ and moisture‐stable solids from the corresponding NHC‐stabilized metal silylidene complexes upon abstraction of the NHC. The formation of these clusters by dimerization of intermediate metal silylidyne (M≡Si) complexes was supported by DFT calculations.

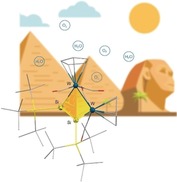

## Introduction

**Figure 1:**
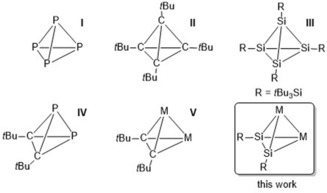
Selected examples of known neutral tetrahedranes.

Tetrahedral clusters that consist of main‐group elements are attractive synthetic targets because of their high ring strain and reactivity.^1^ Even white phosphorus (P_4_), which has been known for centuries, has recently been the subject of intense research (**I**; Figure 1).^2^ The heavier homologue, As_4_, is challenging to handle because of its thermal and photochemical instability. Nevertheless, Cummins and co‐workers even managed to isolate As_3_P, the first example of a hetero‐atomic tetrahedrane.^3^ The archetypical organic tetrahedrane (*t*BuC)_4_ (**II**) was isolated by Maier and co‐workers in 1978,^4^ whereas Wiberg et al. reported the heavier analogue, tetrasilatetrahedrane (*t*Bu_3_SiSi)_4_ (**III**), in 1993.^5^ One decade later, Sekiguchi and co‐workers isolated a further tetrasilatetrahedrane (R_4_Si_4_, R=SiMe((CH(SiMe_3_)_2_)_2_) while trying to isolate a disilyne with a Si≡Si triple bond.^6^ Very recently, another neutral tetrahedron that contains two different heteroatoms, (*t*BuCP)_2_ (**IV**), was reported to form upon dimerization of phosphaalkynes (RC≡P).^7^ In addition to neutral tetrahedral complexes, anionic tetrahedral [E_4_]^4−^ (E=Si, Ge, Sn) species, so‐called Zintl‐type polyanions, are known.^8^ Whilst binary combinations in Zintl tetrahedral clusters [E_*n*_M_4−*n*_] have been reported,^9^ there are no examples of neutral heterobimetallic tetrahedral clusters with heavier main‐group elements and transition metals.

Numerous examples are known of M_2_C_2_‐type bimetallic complexes (**V**) with bridging acetylene or acetylene derivatives that exhibit tetrahedral structures.^10^ Such complexes find application in the Pauson–Khand synthesis of cyclopentanone derivatives and are catalysts in hydroboration reactions,^11^ yet heavier congeners have remained unexplored to the best of our knowledge. Among heavier analogues of Group 14 element compounds M_2_E_2_ (E=Si, Ge, Sn), particular interest has been devoted to silicon as bimetallic clusters with bridging silicon atoms are indeed alleged key intermediates in various transition‐metal‐catalyzed transformations.^12^ Since the 1900s, various M_2_Si_2_ binuclear transition‐metal complexes have been reported and their catalytic activities have been exploited in the dehydrocoupling of hydrosilanes and the metathesis of olefins.^13^ Thus far, however, all of these complexes feature a planar, diamond‐shaped, or butterfly‐type M_2_Si_2_ core, whereas tetrahedral structures remain elusive.13a, 14 Generally, monoatomic tetrahedral derivatives R_4_E_4_ can be generated photochemically from the corresponding planar linear compounds by elimination or photoisomerization as reported for **II**.^1^, ^15^ Accordingly, tetrahedranes can form by dimerization of disilynes or nickel‐mediated dimerization of phosphaalkynes.^7^, ^16^

**Scheme 1:**
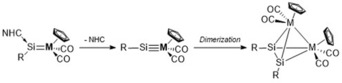
Synthetic strategy to isolate a neutral M_2_Si_2_ tetrahedrane upon dimerization of a transient silylidyne complex (Si≡M).

Herein, we report the first isolable heterobimetallic M_2_Si_2_ cluster with a tetrahedral structure. Inspired by the previous reports from the groups of Wiberg and Sekiguchi, we based our synthetic strategy on the elimination of an N‐heterocyclic carbene (NHC) from a silylidene complex (Si=M) to generate a silylidyne complex (Si≡M), which was hypothesized to dimerize subsequently to a tetrahedral M_2_Si_2_ cage cluster (Scheme 1).

The chemistry of transition‐metal silylidyne complexes has a short, yet spectacular history. The arguably first silylidyne complex [Cp*(dmpe)(H)MoSiMes] (dmpe=PMe_2_CH_2_CH_2_PMe_2_) was reported by Tilley and Mork in 2003.^17^ Shortly after, when the role of the NHC in stabilizing low‐valent silicon(II) species had been recognized, a genuine Mo≡Si triple‐bonded complex was isolated by the group of Filippou using this elegant synthetic approach.^18^ Following this achievement, a handful of transition‐metal silylidyne complexes and their reactivities were reported by further research groups.^17^, ^18^, ^19^ Characteristically, all room‐temperature isolable transition‐metal silylidyne complexes bear very bulky ligands either on the silicon center (e.g., Ar^Trip^, Eind) or on the metal center (e.g., Cp*, Tbb). We concluded that a M≡Si complex with comparably reduced bulk on both the silicon atom and the transition‐metal center should be an excellent choice for the targeted tetrahedral cluster.

## Results and Discussion

**Scheme 2:**
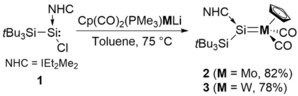
Synthesis of NHC‐stabilized molybdenum (**2**) and tungsten (**3**) silylidene complexes.

Very recently, we reported the synthesis of the first silyl‐substituted chlorosilylene (**1**) and studied the reactivity associated with its lone pair and chloride substituent.^20^ Compound **1** is prone to salt metathesis reactions because of the presence of the chloride atom. In fact, heating an orange toluene solution of chlorosilylene **1** with Cp(CO)_2_(PMe_3_)MLi (M=Mo, W) at 75 °C smoothly gave the IEt_2_Me_2_ (1,3‐diethyl‐4,5‐dimethyl‐imidazolin‐2‐ylidene) stabilized transition‐metal silylidene complexes **2** and **3** as air‐ and moisture‐sensitive, dark‐green solids (**2**, M=Mo, 82 %; **3**, M=W, 78 %; Scheme 2).

The ^29^Si NMR spectra of **2** and **3** show characteristic resonances, which are shifted strongly downfield to 278.8 ppm and 229.7 ppm (^1^ *J*_Si‐W_=261 Hz), respectively, in reference to those of **1** (*δ*=18.3 ppm). Similar chemical shifts were observed for the previously reported transition‐metal silylidene complexes.^18^, 19m, 21 The silicon‐bonded NHC carbene atoms resonate at 168.3 ppm (**2**) and 172.6 ppm (**3**) in the ^13^C NMR spectroscopic analysis, which is similar to the chemical shift found for **1** (*δ*=169.7 ppm).

**Figure 2:**
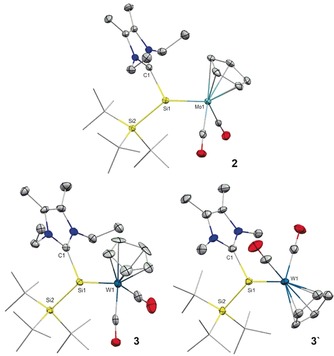
Ellipsoid plots (set at 50 % probability) of the molecular structures of compounds **2** (one out of two independent molecules in the asymmetric unit is shown), **3** (one out of three independent molecules in the asymmetric unit is shown) and **3′**.^39^ Hydrogen atoms are omitted for clarity, and *tert*‐butyl groups are depicted in wireframe for simplicity. Selected bond lengths [Å] and angles [°]: **2**: Si1–Mo1 2.3499(10), Si1–Si2 2.4418(11), Si1–C1 1.949(2); C1‐Si1‐Si2 102.64(6), Mo1‐Si1‐C1 116.33(6), Mo‐Si1‐Si2 141.03(3). **3**: Si1–W1 2.346(2), Si1–Si2 2.428(3), Si1–C1 1.935(7); C1‐Si1‐Si2 105.1(2), W1‐Si1‐C1 113.5(2), W1‐Si1‐Si2 141.38(10). **3′**: Si1–W1 2.3534(12), Si1–Si2 2.4402(16), Si1–C1 1.941(5); C1‐Si1‐Si2 104.15(14), W1‐Si1‐C1 115.67(14), W‐Si1‐Si2 140.15(6).

The IR spectra of **2** and **3** each show two *ν*(CO) absorption bands at 1782 and 1864 cm^−1^ (**2**), and at 1770 and 1849 cm^−1^ (**3**). The positions of these bands agree well with previously reported metal arylsilylidene complexes and our predictions by density functional theory (DFT) calculations (**3**: 1837 and 1892 cm^−1^).^18^, 19l Single crystals of **2** and **3** were obtained from a toluene/pentane (1:3) mixture at ambient temperature, and the structure in the solid state was determined by X‐ray diffraction analysis (Figure 2). Complex **2** features a Mo=Si double bond (2.3499(10) Å), which is shorter than that found for the donor‐free molybdenum silylidene complex Cp*(CO)_2_(SiMe_3_)Mo=Si(Mes)_2_ (2.3872(7) Å) and lies in the range of previously reported molybdenum silylidene complexes (*d*(Mo−Si)=2.288(2)–2.3872(7) Å).^22^ Similarly, **3** exhibits a W=Si (2.346(2) Å) bond that is considerably shorter than in [Cp*W(CO)_2_(=SiMes_2_)(SiMe_3_)] (2.3850(12) Å) and in the neutral alkyl(silylidene)tungsten complex [(η^5^‐C_5_Me_4_Et)(CO)_2_(H)W=Si{C(SiMe_3_)_3_}] (2.3703(11) Å), but slightly longer than that of the anionic complex [Cp*(CO)_2_W=SiH{C(SiMe_3_)_3_}][H^Me^I^*i*^Pr] (2.3367(17) Å).^21^, ^23^ UV/Vis analysis (Figure S6) revealed a characteristic absorption band at *λ* ^max^=418 nm for the Mo=Si complex **2**. For the W=Si complex **3**, a band at *λ* ^max^=418 nm and a very broad and weak band ranging from approximately *λ=*500 to 700 nm were found (Figure S12). Time‐dependent DFT (TD‐DFT) calculations reproduced these values very well (Figure S63). In addition, the Löwdin population analysis indicates that **3** has a d^4^ electron configuration with considerable negative partial charge at the tungsten atom, which is consistent with an oxidation state of +II. Both complexes **2** and **3** feature trigonal‐planar‐coordinated silicon centers, with the sum of bond angles at the silicon center being 360°.

**Scheme 3:**
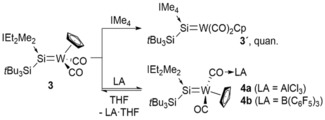
Synthesis of **3′** and **4 a**, **b** from NHC‐stabilized silyl(silylidene) tungsten complex **3**.

We investigated the replacement of the NHC moiety and treated the bulky IEt_2_Me_2_ compound with more nucleophilic IMe_4_.^24^ Indeed, treatment of **3** with excess IMe_4_ resulted in quick conversion (60 % in 30 min) and eventually, after 12 h, quantitative exchange of IEt_2_Me_2_ by IMe_4_ (**3′**; Scheme 3). As expected, only minor shifts in reference to the starting material were observed in all (^1^H, ^13^C, ^29^Si) NMR experiments. Single crystals of **3′** were obtained at ambient temperature from a C_6_D_6_/pentane (1:1) mixture, and the molecular structure was also confirmed by X‐ray diffraction analysis (Figure 2). The W−Si bond is slightly elongated for **3′** (2.3534(12) Å) in comparison to **3** (2.346(2) Å); nevertheless, the structural parameters are very similar.

**Figure 3:**
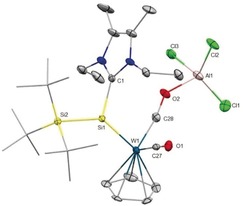
Ellipsoid plot (set at 30 % probability) of the molecular structure of compound **4 a**. Hydrogen atoms are omitted for clarity, and *tert*‐butyl groups are depicted in wireframe for simplicity.^39^ Selected bond lengths [Å] and angles [°]: Si1–W1 2.3630(18), Si1–Si2 2.437(2), Si1–C1 1.940(10), Al1–O2 1.777(8), W1–C27 1.975(7), W1–C28 1.840(7); C1‐Si1‐Si2 106.4(6), W1‐Si1‐C1 114.6(6), W‐Si1‐Si2 138.89(9).

The ligand exchange ability of **3** encouraged us to abstract the NHC by treatment with Lewis acids. Indeed, treatment of **3** with the strong Lewis acid B(C_6_F_5_)_3_ or more oxophilic AlCl_3_ resulted in an immediate color change from dark green to dark red in toluene (Scheme 3). Significant downfield shifts of the ^29^Si NMR resonances for **4 a** (*δ*=322.0 ppm) and **4 b** (*δ*=323.2 ppm) compared to that of **3** (*δ*=229.7 ppm) were observed, whereas the ^13^C NMR spectra of **4 a** and **4 b** displayed only a very small change in shift (166.9 ppm for **4 a** and 167.3 ppm for **4 b**) from **3** (^13^C *δ*=172.6 ppm). This suggests that the NHC remains coordinated to a silylidene unit. Intriguingly, the addition of coordinating THF to the red solutions of **4 a** or **4 b** resulted in the regeneration of the dark‐green color, and the ^1^H NMR spectroscopic analysis confirmed the regeneration of the initial starting material **3**. This reaction corroborated the formation of a peculiar Lewis acid–carbonyl adduct, and was likewise confirmed by X‐ray crystal structure analysis of **4 a** (Figure 3). A comparable terminal carbonyl ligand activation was observed by Cummins and co‐workers during preparation of a terminal molybdenum carbide upon acylation of a Mo^II^ carbonyl complex with pivaloyl chloride.^25^

The W−Si bond in **4 a** (2.3630(18) Å) is elongated in reference to **3** (2.346(2) Å) because of the reduced back‐donation from tungsten to silicon and in line with the downfield‐shifted resonance in the ^29^Si spectrum (*δ*=322.0 ppm). The W−C bond length for the AlCl_3_‐coordinated CO ligand (1.840(7) Å) is significantly shortened in comparison with that of the terminal carbonyl ligand (1.975(7) Å) and even in the range of tungsten carbyne complexes (1.82–1.87 Å).^26^ The Al−O bond length in **4 a** (1.777(8) Å) is comparable with previously reported AlCl_3_ coordinated to the oxygen atom of a carbonyl ligand without bond rupture (1.812(2) Å).^27^ Compound **4 a** has a similar absorption band at 400 nm in the UV/Vis spectrum in toluene. Unfortunately, **4 b** could only be isolated as a sticky solid and not in crystalline form. The IR spectrum of **4 a** in the solid state shows two *ν*(CO) bands that appear as broad bands at 1813 cm^−1^ for AlCl_3_‐coordinated CO and at 1901 cm^−1^ for the terminal CO. Two peculiar carbonyl stretching frequencies are observed because of enhanced π‐back‐donation from tungsten to the carbonyl ligand and simultaneous weakening of the C−O bond (C27–O1 1.158(8) Å, C28–O2 1.255(9) Å) that is coordinated to AlCl_3_. Similarly, **4 b** also shows two *ν*(CO) bands at 1906 cm^−1^ and 1873 cm^−1^. The latter can be assigned to CO⋅⋅⋅B(C_6_F_5_)_3_, which appears at a higher wavenumber than in [Cp*(CO)−{C_6_F_5_)_3_B⋅⋅⋅OC}W=Si(H)Tsi][H^Me^I^i^Pr] (ν(CO⋅⋅⋅BCF)=1535 cm^−1^).19a This observation indicates weaker coordination of the borane to the carbonyl group in comparison to that of the anionic silylidene complex.

**Scheme 4:**
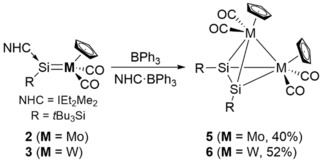
Formation of heterobimetallic tetrahedral compounds **5** and **6** by abstraction of the NHC from TM silylidene complexes **2** and **3** by BPh_3_.

In order to abstract the NHC from the silylidene complexes, we hence used a milder Lewis acid, namely triphenylboron (BPh_3_, Scheme 4). Indeed, heating toluene solutions of **2** and **3** with one equivalent of BPh_3_ for 30 min to 90 °C afforded the desired tetrahedral clusters **5** and **6** in 40 % and 52 % yield, respectively. These heterobimetallic compounds are well soluble in aromatic as well as aliphatic organic solvents. Surprisingly, the heterobimetallic tetrahedral Si_2_M_2_ clusters **5** and **6** are perfectly stable in moist air, as has been also reported for tetrasilatetrahedrane (*t*Bu_3_SiSi)_4_ (**III**). It is interesting to note that these complexes did not even react with methanol when heated to 70 °C for 24 h.

**Figure 4:**
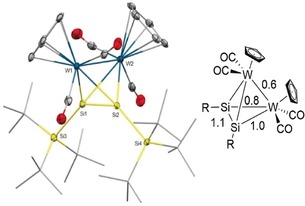
Ellipsoid plot (set at 30 % probability) of the molecular structure of compound **6** (left; one of three independent molecules in the asymmetric unit is shown) and bond orders as predicted by the Löwdin population analysis (right). Hydrogen atoms are omitted for clarity, and *tert*‐butyl groups are depicted in wireframe for simplicity. Selected bond lengths [Å] and angles [°]: Si1–W1 2.5507(15), Si1–W2 2.6913(15), Si2–W1 2.6790(14), Si2–W2 2.5593(14), W1–W2 3.0732(8), Si1–Si2 2.2221(19); W1‐Si1‐W2 71.73(4), W1‐Si1‐Si2 67.89(5), W2‐Si1‐Si2 61.92(5).

The single‐crystal structure of **6** revealed the formation of a tetrahedral, bimetallic transition‐metal silicon cluster (Figure 4). The W−Si (2.5507(15)–2.6913(15) Å) bonds in **6** are significantly longer than those in **3** (2.346(2) Å) and reported W=Si double bonds (2.34–2.47 Å), and fall in the range of W−Si single bonds (2.47–2.71 Å).^21^, ^23^, ^28^, ^29^ The structural parameters of complex **6** are akin to the planar complexes W‐Si‐W‐Si (W–W 3.183(1) Å, W–Si 2.586(5)–2.703(4) Å) and W‐Si‐W‐H (W–Si 2.489(2)–2.487(2) Å).14c, 30 The Si−Si bond (2.2221(19) Å) is significantly shorter than those in previously reported MSiSiM butterfly‐shaped clusters (*d*(Si⋅⋅⋅Si)=2.85–2.98 Å) and also those of tetrasilatetrahedranes, where the Si−Si bond lengths range from 2.315(2) to 2.3830(19) Å.^5^, ^6^, 14a, 31 The short Si–Si separation is attributed to the partial multiple bond character (Figure 4, right) and reduced σ‐donation from the silyl substituents due to the elongation of the exocyclic Si−Si bonds (Si1–Si3 2.437(2) Å, Si2–Si4 2.430(2) Å). The formation of bimetallic clusters for **5** and **6** was also confirmed by mass‐spectrometric analysis (Figures S36–S46), which suggested that **5** is isostructural with **6**. The tetrahedral M_2_Si_2_ clusters melt below 70 °C (m.p. 64–65 °C for **5**; 67–69 °C for **6**), yet we did not observe any changes in the ^1^H NMR spectra upon heating xylene solutions to 120 °C. This observation corroborates the high thermal stability and the absence of an equilibrium between the monomer (Cp(CO)_2_M≡SiSi*t*Bu_3_) and the dimeric forms of **5** and **6**, and is consistent with diffusion NMR experiments (DOSY; Figures S28 and S40). The ^29^Si NMR signals of skeletal silicon atoms shifted strongly to higher fields (*δ*=3.65 ppm for **5**, GIAO=5 ppm; *δ*=−63.04 ppm (^1^ *J*_Si‐W_=52 Hz) for **6**; GIAO=−48 ppm), which corroborates strong silyl character due to a change in hybridization from sp^2^ to sp^3^.19j, 32 In addition, such an upfield shift is also typically observed for ring C atoms of tetrahedranes.^1^, ^4^, 15a, 33 The small ^1^ *J*_Si−W_ coupling constant of 52 Hz indicates a high degree of p‐character at the Si atom and a relatively small contribution from the silicon's s‐orbital. A significant low‐field shift was observed for the supersilyl ligand (*δ*=48.32 ppm for **5**; 43.99 ppm for **6**). Similar shifts for *t*Bu_3_Si were also reported for Wiberg's tetrasilatetrahedrane (*t*Bu_3_SiSi)_4_ (^29^Si, *δ*=53.07 ppm; **III**, Figure 1).^5^ The IR spectra of **5** and **6** showed two *ν*(CO) bands at higher wavenumbers than for the NHC‐TM‐silylidenes **2**–**4 a**, **b** (1844 and 1918 cm^−1^ for **5**; 1860 and 1914 cm^−1^ for **6**; DFT: 1904 and 1916 cm^−1^). This hypsochromic shift is due to reduced π‐back‐donation from the transition metal to the CO ligand. The UV/Vis spectra of **5** and **6** in toluene showed absorption bands at 543 nm and 530 nm, respectively. In excellent agreement, TD‐DFT calculations for **6** predict the HOMO–LUMO transition to lie at 501 nm.

**Figure 5:**
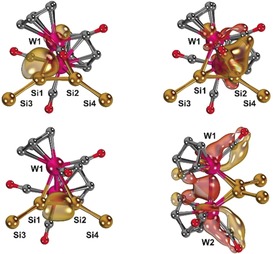
Intrinsic bond orbitals (IBOs) associated with the Si1−W1 (top, left), Si1−W2 (top, right), Si1−Si2 (bottom, left) and W1−W2 (bottom, right) bonds (*t*Bu groups and hydrogen atoms are omitted for clarity, but were included in the calculations).

We performed detailed DFT calculations at the PBE0‐D3BJ(SMD)/ZORA‐def2‐TZVPP//PBE0‐D3BJ/def2‐SVP level of theory in order to understand the electronic structures of **6** and **3** (Figures S57–S62).^34^, ^35^ Indeed, Löwdin population analysis of the DFT‐optimized structure of **6** also indicated a Si−Si bond order of 1.1, indicative of only very small multiple bond character (Figure 4, right), which is in line with the short Si−Si bond length in **6**. Interestingly, the calculations suggest a higher Si–Si multiple bond character of 1.3 for the molybdenum complex **5** (Figure S61), which is in line with the relative ^29^Si NMR shifts of **5** and **6** (see above). Plotting the HOMO (highest occupied molecular orbital) and LUMO (lowest unoccupied molecular orbital) corroborates delocalization of both orbitals over the whole cluster (Figure S58). The intrinsic bond orbitals (IBOs)^36^ show two different, yet quite covalent, σ‐interactions between Si1 and W1 or W2, respectively (Figure 5, top). The Si1−W1 σ‐bond is polarized towards Si, whereas the longer Si1−W2 bond features additionally a π‐back‐bonding interaction with the CO π* orbitals. Besides, we found a Si1−Si2 σ‐bond as well as considerable bonding interactions between the W1 and W2 atoms (Figure 5, bottom). Overall, the calculations confirmed a tetrahedral structure with strong and covalent interactions between all silicon and tungsten atoms.

**Figure 6:**
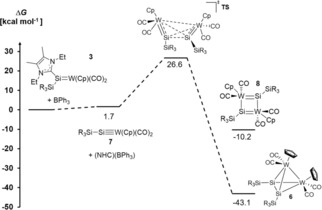
Proposed reaction profile for the formation of W_2_Si_2_ tetrahedral cluster **6**.

Furthermore, the reaction mechanism for the formation of **6** was modeled in order to understand the peculiar Si–Si dimerization (Figure 6).^37^ The restricted DFT calculations suggest that the formation of the intermediate silylidyne complex **7** proceeds essentially in isoergic fashion (Δ*G*= +1.7 kcal mol^−1^). The subsequent dimerization (Δ*G*=−43.1 kcal mol^−1^) features a barrier of Δ*G* ^≠^=+26.6 kcal mol^−1^, which agrees well with a reaction occurring at elevated temperatures. The transition state (Figure 6) shows a very large separation of the two tungsten atoms (5.05 Å) and hence is indicative of only weak interactions between these two atoms. Nevertheless, a very small orbital overlap in HOMO‐3 substantiates a very asynchronous, yet still concerted formation of the Si−Si and W−W bonds.^38^

Most importantly, the transition state reveals that the steric bulk associated with the supersilyl groups and the Cp substituents allows only for a perpendicular arrangement of the two silylidyne groups. This orientation consequently determines the formation of the tetrahedral cluster instead of four‐membered rings as would be expected by simplifying polarity considerations. Indeed, attempts to geometry‐optimize four‐membered rings with either Si−Si/W−W or alternating Si−W bonds led to isomeric tetrahedra (Δ*G*=−34.0 kcal mol^−1^; Figure S70), three‐membered rings (Δ*G*=−15.8 kcal mol^−1^; Figure S70), or quadrangles of much higher energy (Si‐W‐Si‐W quadrangle **8**: Δ*G*=−10.2 kcal mol^−1^). Attempts to model dimeric compounds with decoordination of only one NHC also did not meet with success. We conclude that the steric bulk in **6** prevents the formation of quadrangles or triangles and allows only for the formation of a tetrahedral cluster subsequent to abstraction of the NHC ligand.

## Conclusion

We have reported the isolation of the heteroatomic, bimetallic M_2_Si_2_ tetrahedral clusters **5** and **6**. These compounds form after NHC abstraction from the respective NHC‐stabilized silylidene complexes **2** and **3** by dimerization of transient transition‐metal silylidyne complexes. These tetrahedral clusters are air‐ and moisture‐stable unlike many other main‐group organometallic compounds. Furthermore, we have shown that the NHC (IEt_2_Me_2_) in complex **3** can be exchanged for a more nucleophilic NHC (IMe_4_). Contrarily, the addition of stronger Lewis acids such as AlCl_3_ or B(C_6_F_5_)_3_ resulted in reversible activation of the carbonyl ligands (**4 a**, **b**). Calculations confirm the covalent bonding in the cluster and indicate that steric bulk is crucial for the formation of the tetrahedron‐type structure.

## Conflict of interest

The authors declare no conflict of interest.

## Supporting information

As a service to our authors and readers, this journal provides supporting information supplied by the authors. Such materials are peer reviewed and may be re‐organized for online delivery, but are not copy‐edited or typeset. Technical support issues arising from supporting information (other than missing files) should be addressed to the authors.

Supplementary
