# Supplementary material for: An Air‐Stable Heterobimetallic Si2M2 Tetrahedral Cluster
Source: Angew Chem Int Ed Engl. 2020 Feb 20;59(14):5823–9. doi: 10.1002/anie.201916116 (PMC7154520; doi:10.1002/anie.201916116)
Supplement: Supplementary file 1 — Supplementary [file ANIE-59-5823-s001.pdf]

Angewandte Chemie
Eine Zeitschrift der Gesellschaft Deutscher Chemiker

Supporting Information

An Air-Stable Heterobimetallic Si_2M_2 Tetrahedral Cluster

*Gizem Dübek, Franziska Hanusch, Dominik Munz, and Shigeyoshi Inoue**

anie_201916116_sm_miscellaneous_information.pdf

Supporting Information

Contents

1. Experimental Section S2

General Methods and Instrumentation S2

Synthesis of $Cp(CO)_2Mo{=}Si(Si\textit{t}Bu_3)(IEt_2Me_2)$ (**2**) S3

Synthesis of $Cp(CO)_2W{=}Si(Si\textit{t}Bu_3)(IEt_2Me_2)$ (**3**): S6

Synthesis of $Cp(CO)_2W{=}Si(Si\textit{t}Bu_3)(IMe_4)$ (**3`**): S11

$[Cp(CO)(Cl_3Al\cdots OC)W{=}Si(Si\textit{t}Bu_3)(IEt_2Me2)$ (**4a**) S13

$[Cp(CO)((C_6F_5)_3B\cdots OC)W{=}Si(Si\textit{t}Bu_3)(IEt_2Me2)$ (**4b**): S13

$[Cp(CO)_2MoSi(Si\textit{t}Bu_3)]_2$ (**5**): S18

$[Cp(CO)_2WSi(Si\textit{t}Bu_3)]_2$ (**6**): S25

Isolation of $IEt_2Me_2{\cdot}BPh_3$ S32

2. Single Crystal X-ray structure determination S35

3. Computational Data S40

General S40

4. References S63

1. Experimental Section

General Methods and Instrumentation

All experiments and manipulations were carried out under argon atmosphere using standard Schlenk techniques or in an MBraun inert-atmosphere glovebox unless otherwise stated. Glassware was heat dried under vacuum prior to use. Solvents were dried by standard methods. NMR spectra at ambient temperature (298 K) were recorded on a Bruker AV400US, DRX400, AVHD300, or AV500C device. δ(^{1}H) and δ(^{13}C) were referenced internally to the relevant residual solvent resonances. δ(^{29}Si) was referenced to the signal of tetramethylsilane (TMS) (δ = 0 ppm) as external standard. Some NMR spectra include resonances for silicone grease (C_6D_6: δ(^{1}H) = 0.29 ppm, δ(^{13}C) = 1.4 ppm and δ(^{29}Si) = –21.8 ppm) derived from B. Braun Melsungen AG Sterican® cannulas. Elemental analyses (EA) were conducted with a EURO EA (HEKA tech) instrument equipped with CHNS combustion analyzer and melting points (m.p.) were determined in small glass capillaries under air by a Büchi M-565 melting point apparatus by microanalytical laboratory of the Catalysis Research Center, Technische Universität München. IR spectra were recorded on a Perkin Elmer FT-IR spectrometer (diamond ATR) in a range of 400–4000 cm^{-1} at room temperature inside an argon-filled glovebox. Mass spectrometry data were acquired using an Exactive Plus Orbitrap system (ionization method: LIFDI) by Thermo Fisher Scientific. The compounds 1,3,4,5-tetramethylimidazol-2-ylidene (IMe_4)[1], $Cp(CO)_2(PMe_3)MLi$[2] and *t*$Bu_3SiSi(Cl)(IEt_2Me_2)$[3] were prepared according to literature procedures. Commercially available chemicals (BPh_3, $AlCl_3$ and $B(C_6F_5)_3$) were purchased from *abcr GmbH* or *Tokyo Chemical Industry Co., Ltd* and used without further purification. Abbreviations: s = singlet, d = doublet, t = triplet, m = multiplet, br = broad, n.a. = not applicable/no answer, n.o. = not observed, SCXRD = Single Crystal X-ray diffraction, IG = Inverse-Gated, INEPT = Insensitive Nuclei Enhanced by Polarization Transfer, HMBC = Heteronuclear Multiple Bond Correlation.

Synthesis of $Cp(CO)_2Mo{=}Si(Si\textit{t}Bu_3)(IEt_2Me_2)$ (2) : $\textit{t}Bu_3Si(Cl)Si{:}\leftarrow IEt_2Me_2$ (**1**) (500 mg, 1.2 mmol) and $CpMo(CO)_2PMe_3Li$ (380 mg, 1.26 mmol, 1.05 equiv.) were mixed in 15 mL toluene and the suspension heated to 75 ˚C overnight. During this time almost all solids were dissolved and color of reaction changed from orange to dark green. Suspension filtered from colorless precipitate (LiCl) and toluene was removed under vacuum to yield dark green residue. Residue filtered by toluene:pentane mixture (10 mL:30 mL) from insoluble brown material. Suitable crystals for single X-ray diffraction analysis were obtained by toluene:pentane (1:3) mixture of compound **2** at room temperature. Yield: 590 mg (82%)

^{1}H NMR (400 MHz, C_6D_6, 298K): δ 5.44 (s, 5H, C_5H_5), 4.32 (m, 2H, N-CH_2CH_3), 3.62 (m, 2H, N-CH_2CH_3), 1.42 (s, 6H, C-CH_3), 1.35 (s, 27H, (($CH_3)_3C$)), 1.25 (t, 6H, N-CH_2CH_3).

^{13}C NMR (101 MHz, C_6D_6, 298K): δ 241.51 (CO), 168.26 (:CN_2), 125.79 ($CH_3C{=}CCH_3$), 90.14 (C_5H_5), 42.59 (N-CH_2CH_3), 32.57 ($C(CH_3)_3$), 24.96 ($C(CH_3)_3$), 14.58 (N-CH_2CH_3), 8.33 ($CH_3C{=}CCH_3$).

^{29}Si{^{1}H} NMR (80 MHz, C_6D_6, 298K): δ 278.76 (*Si*=Mo), 6.36 (*t*Bu_3*Si*)

^{29}Si INEPT NMR (80 MHz, C_6D_6, 298K): δ 6.36 (*t*Bu_3*Si*)

IR (ATR, neat) [cm^{-1}]: υ(CO) = 1782, 1864

EA: $C_{28}H_{48}MoN_2O_2Si_2$; Calculated [%]: C (56.35), H (8,11), N (4.69); Measured: C (56.34), H (8.12), N (4.46)

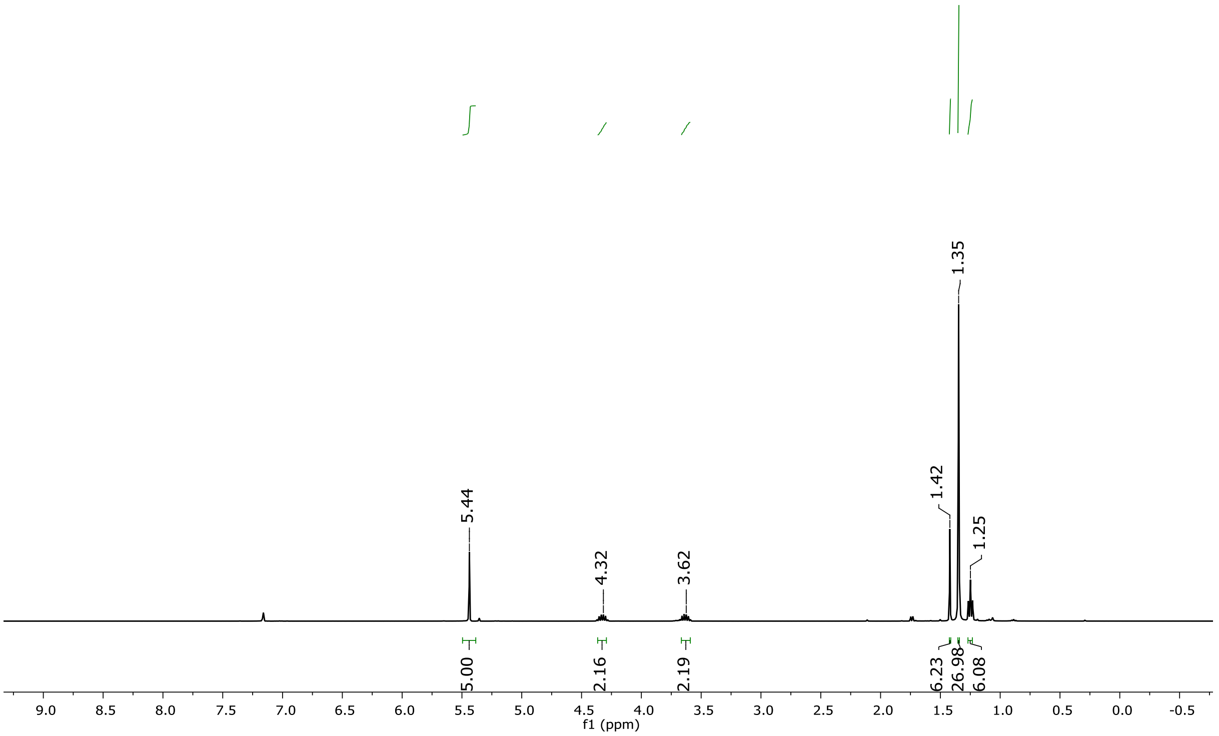

Figure S1. ^{1}H spectrum of compound **2** in C_6D_6 at 298 K.

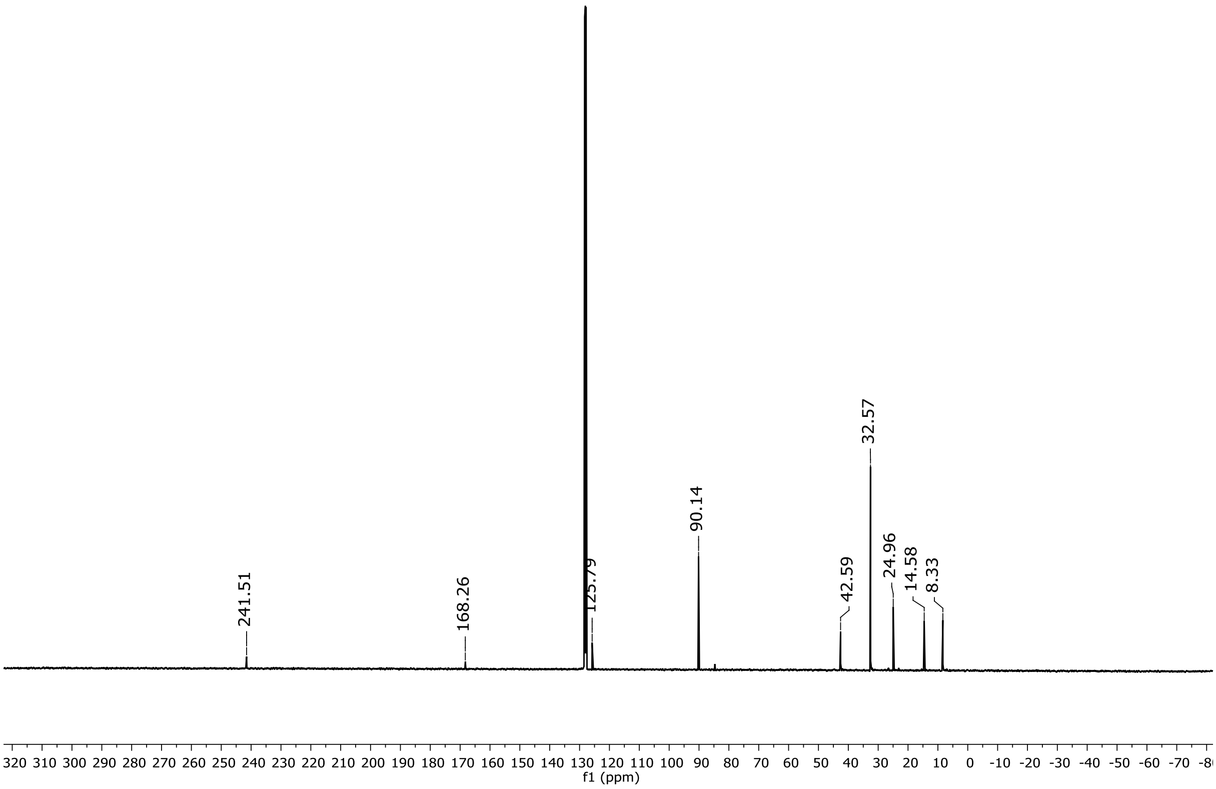

Figure S2. ^{13}C spectrum of compound **2** in C_6D_6 at 298 K.

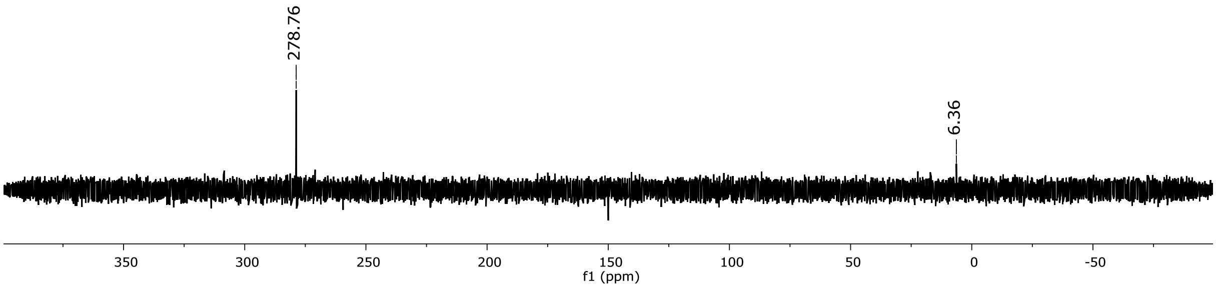

Figure S3. $^{29}Si\{^1H\}$ spectrum of compound **2** in C_6D_6 at 298 K.

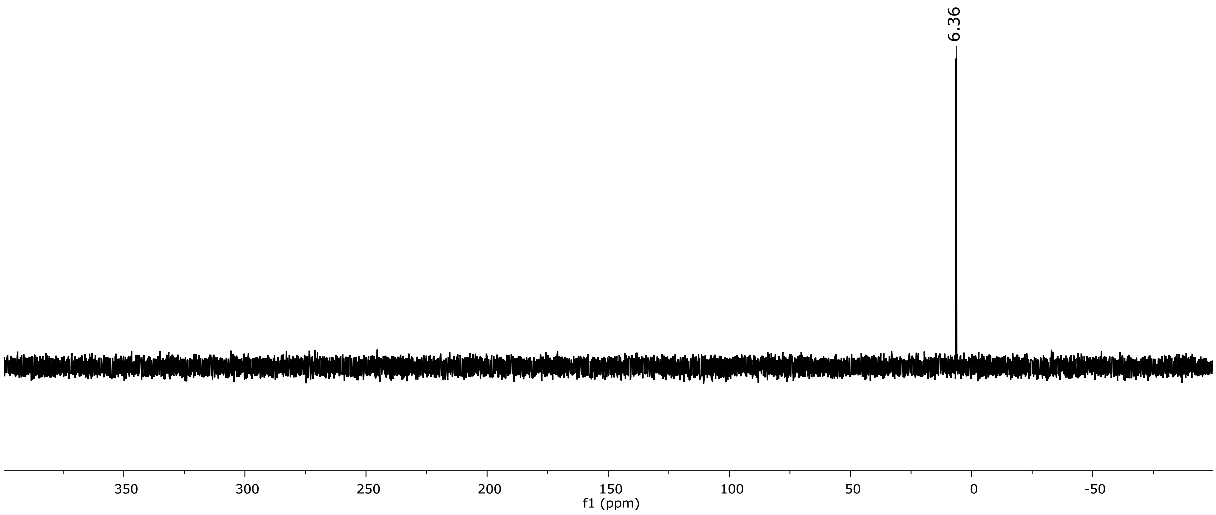

Figure S4. ^{29}Si-INEPT spectrum of compound **2** in C_6D_6 at 298 K.

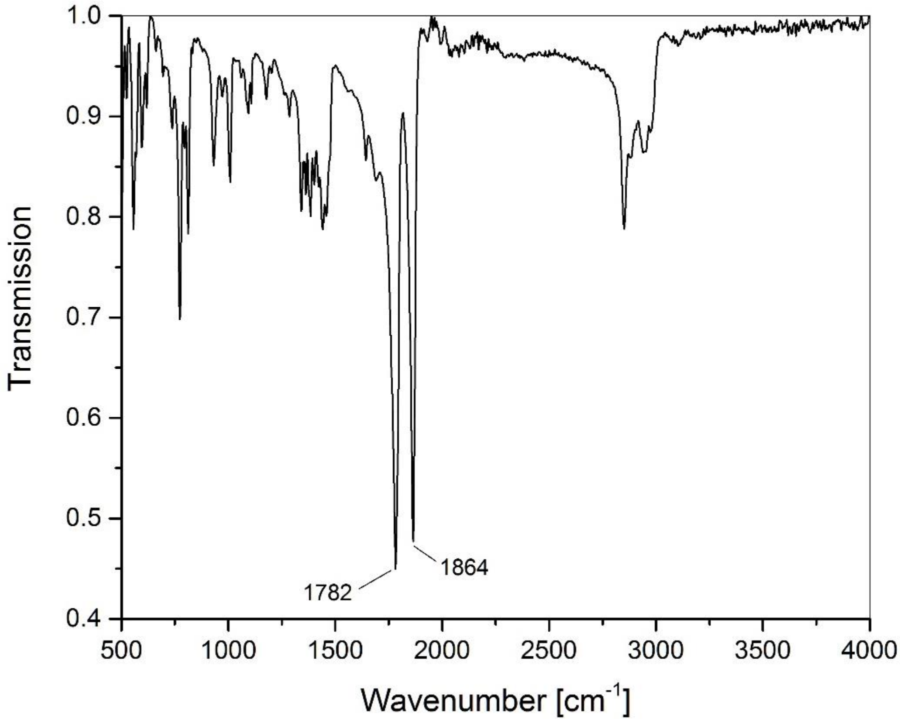

Figure S5. IR Spectrum of compound **2**. (ATR, neat)

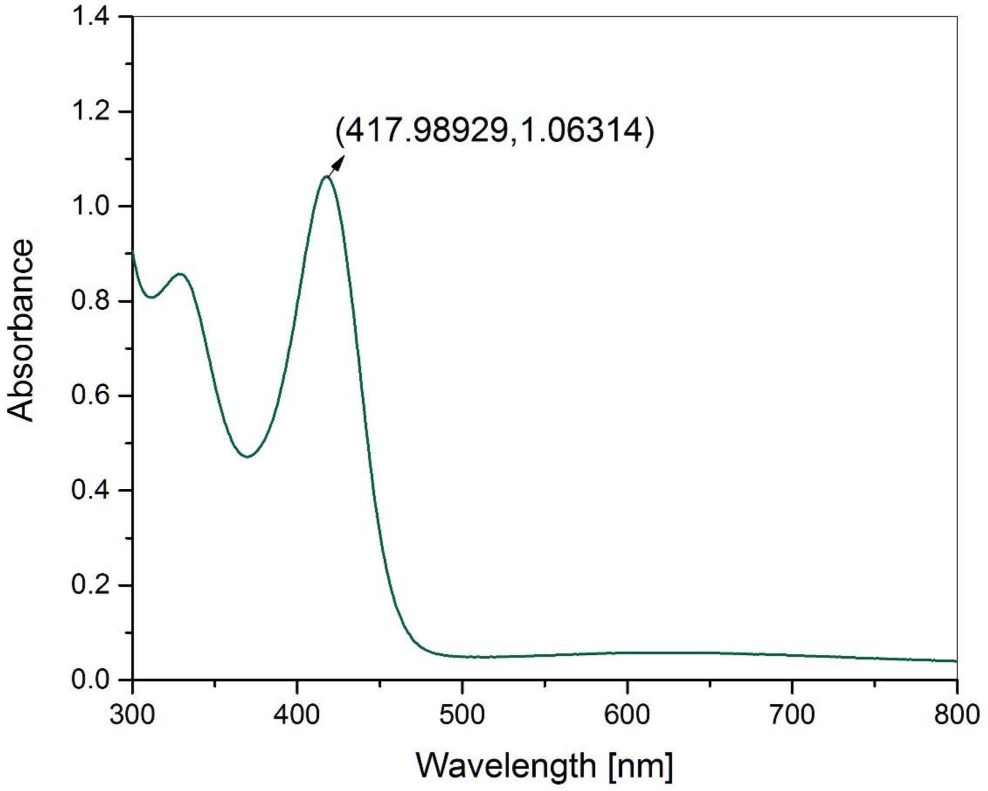

Figure S6. UV-Vis spectra of compound **2** in toluene. (Conc. 1.173 x 10^{-4}M; Ɛ= 9063 L mol $^{-1}$cm^{-1})

Synthesis of $Cp(CO)_2W$=Si(Si*t*Bu_3)(IEt_2Me_2) (3): *t*Bu_3Si(Cl)Si: ←IEt_2Me_2 (**1**) (500 mg, 1.2 mmol) and $CpW(CO)_2PMe_3Li$ (490 mg, 1.26 mmol, 1.05 equiv.) were mixed in 15 mL toluene and the suspension heated to 75 ˚C overnight. During this time almost all solids were dissolved and color of reaction changed from orange to dark-brown green. Suspension filtered from colorless precipitate (LiCl) and toluene was removed under vacuum to yields dark green residue. Residue filtered by toluene:pentane mixture (15 mL:20mL) from insoluble brown material. Suitable crystals for single X-ray diffraction analysis were obtained by toluene:pentane (1:1) mixture of compound **3** at 4 ˚C. Yield: 640 mg (78%).

^{1}H NMR (400 MHz, C_6D_6, 298K): δ 5.38 (s, 5H, C_5*H_5*), 4.54 (m, 2H, N-C*H_2*CH_3), 3.80 (m, 2H, N-C*H_2*CH_3), 1.43 (s, 6H, C-C*H_3*), 1.34 (s, 27H, ((C*H_3*)$_3$C)), 1.21 (t, 6H, N-CH_2C*H_3*).

^{13}C NMR (101 MHz, C_6D_6, 298K): δ 232.77 (CO), 172.62 (:*C*N_2), 125.60 (CH_3*C*=*C*CH_3), 88.68 (*C*$_5H_5$), 40.95 (N-*C*H_2CH_3), 32.51 (*C*(CH_3)$_3$), 24.45 (C(*C*H_3)$_3$), 14.63 (N-CH_2*C*H_3), 8.28 (*C*H_3C=C*C*H_3).

^{29}Si{^{1}H} NMR (99 MHz, C_6D_6, 298K): δ 229.71 (*Si*=W, $^{1}J_{WSi}$ = 261 Hz), 12.32 (*t*Bu_3*Si*)

^{29}Si INEPT NMR (80 MHz, C_6D_6, 298K): δ 12.38 (*t*Bu_3*Si*)

IR (ATR, neat) [cm^{-1}]: υ(CO) = 1770, 1849

EA: $C_{28}H_{48}WN_2O_2Si_2$; Calculated [%]: C (49.12), H (7.07), N (4.09); Measured: C (51.38), H (7.79), N (3.90)

LIFDI-MS [m/z]: calculated (for $C_{28}H_{48}N_2O_2Si_2W$): 684.2765, observed: 684.2765

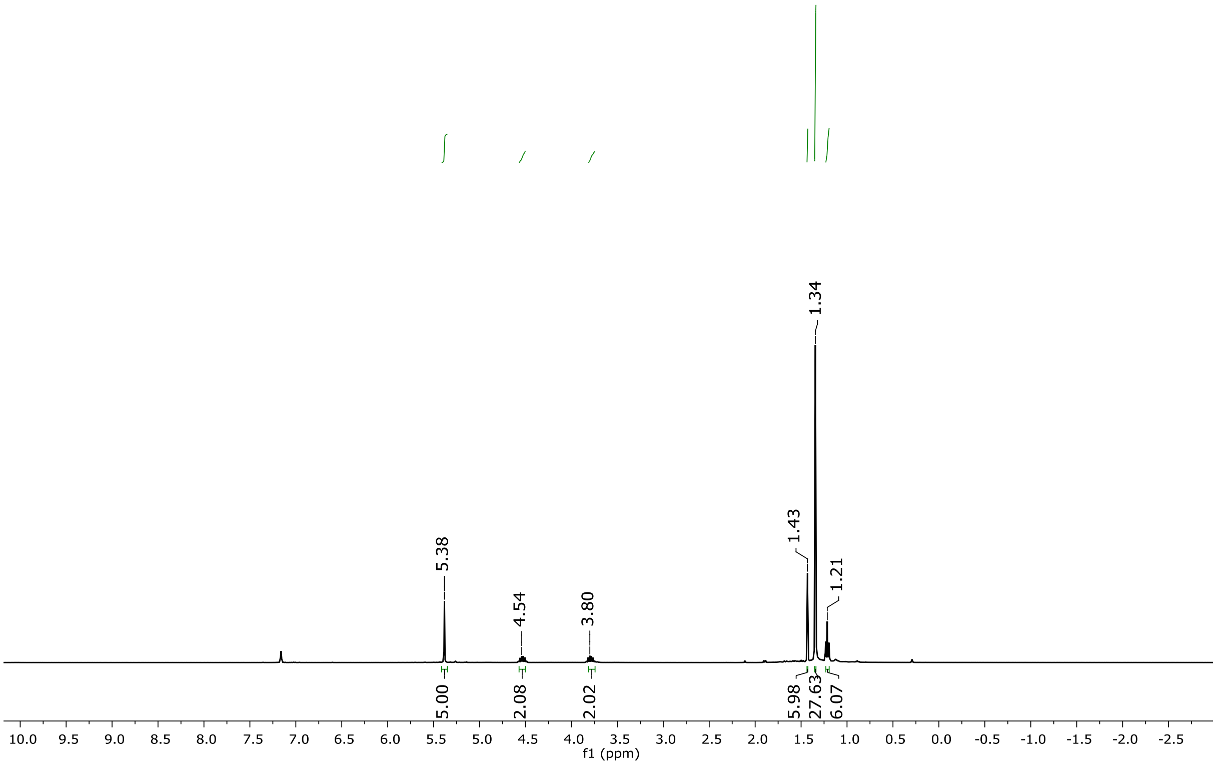

Figure S7. ^{1}H spectrum of compound **3** in C_6D_6 at 298 K.

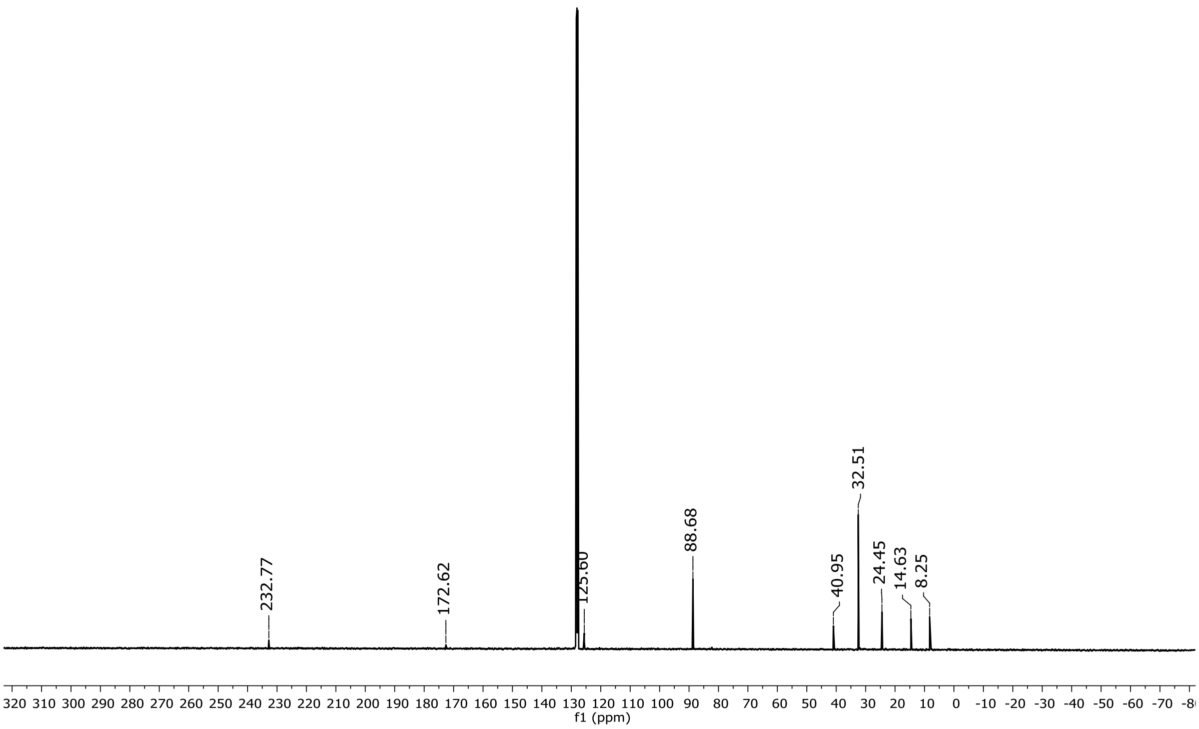

Figure S8. ^{13}C spectrum of compound **3** in C_6D_6 at 298 K.

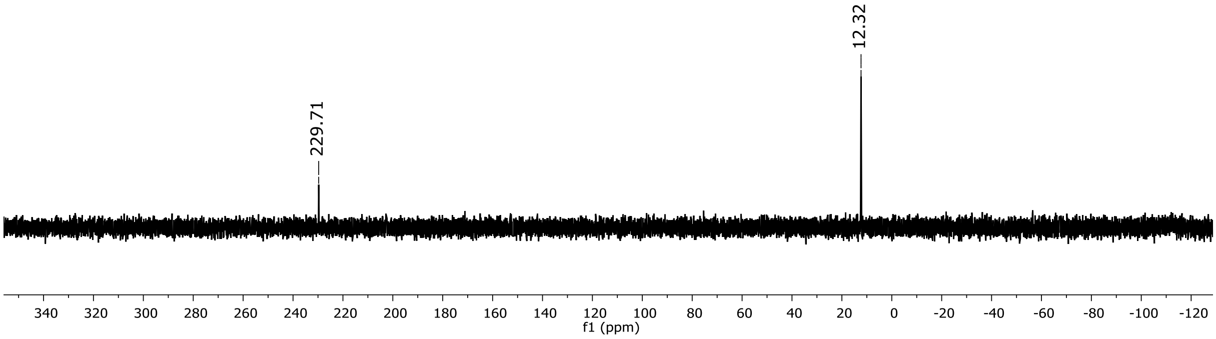

Figure S9. $^{29}Si\{^{1}H\}$ spectrum of compound **3** in C_6D_6 at 298 K.

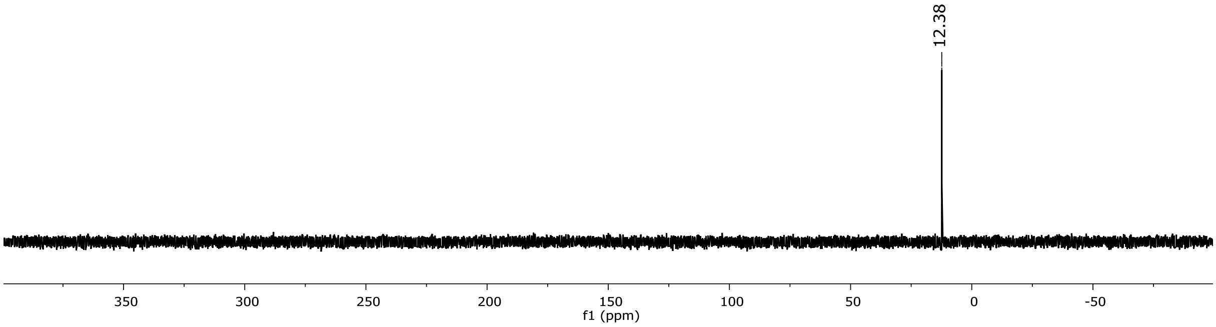

Figure S10. ^{29}Si-INEPT spectrum of compound **3** in C_6D_6 at 298 K.

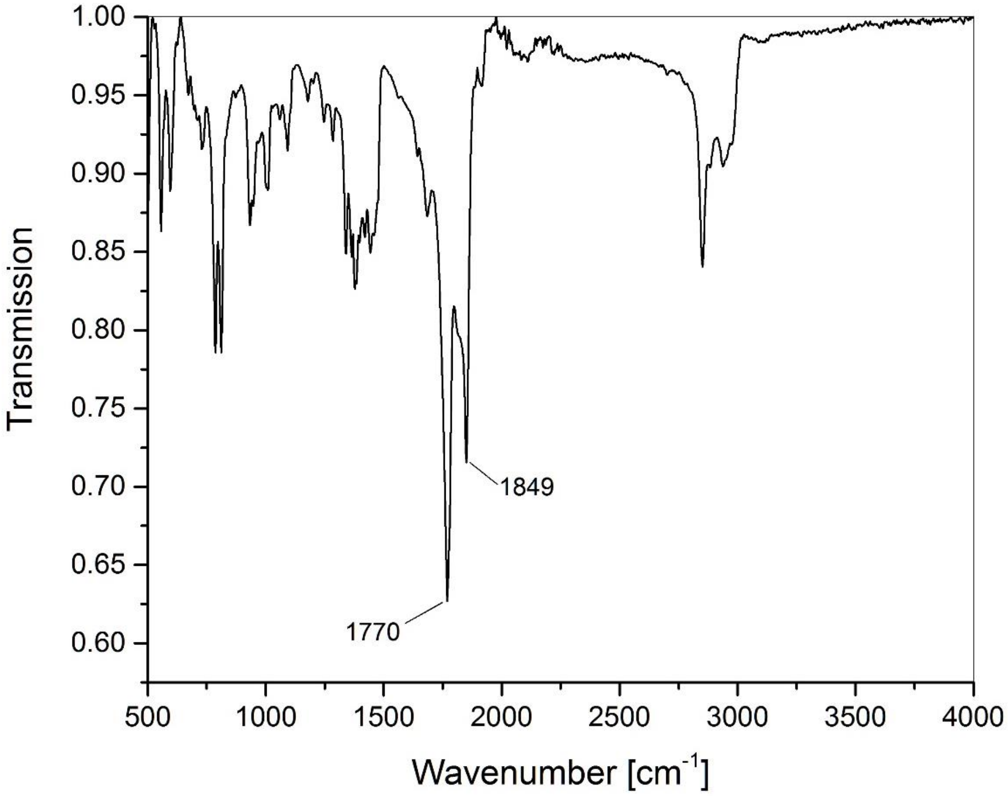

Figure S11. IR Spectrum of compound **3**. (ATR, neat)

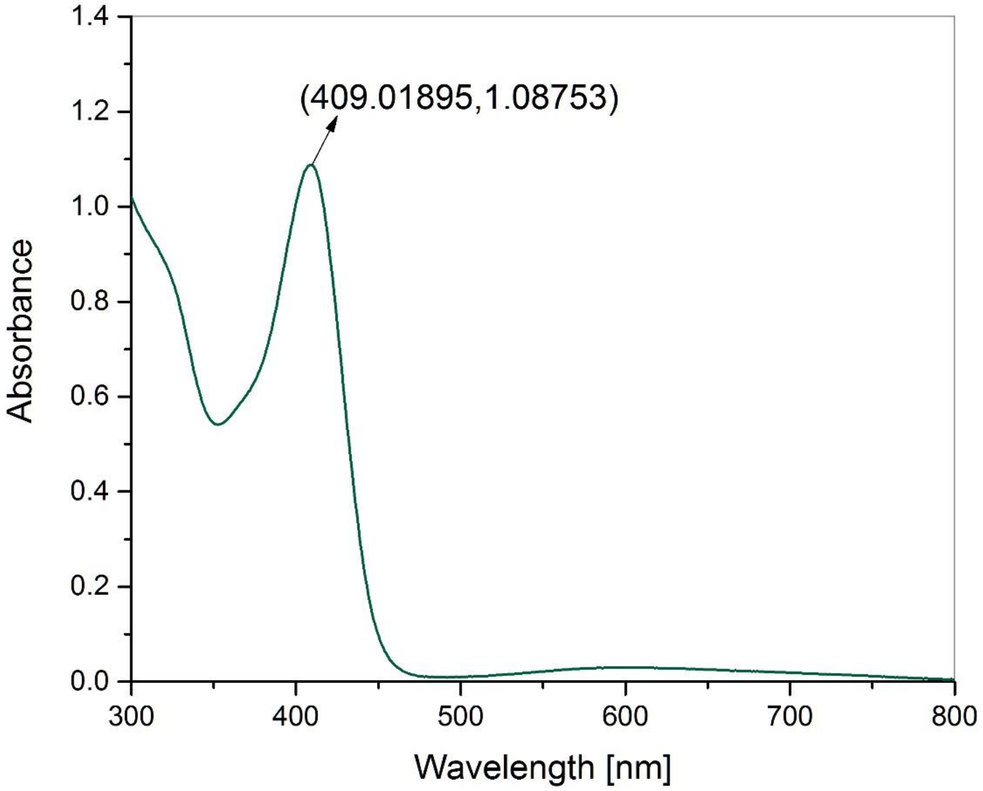

Figure S12. UV-Vis spectra of compound **3** in toluene. (Conc. 1.168 x 10^{-4}M; Ɛ= 9311 L mol $^{-1}$cm^{-1})

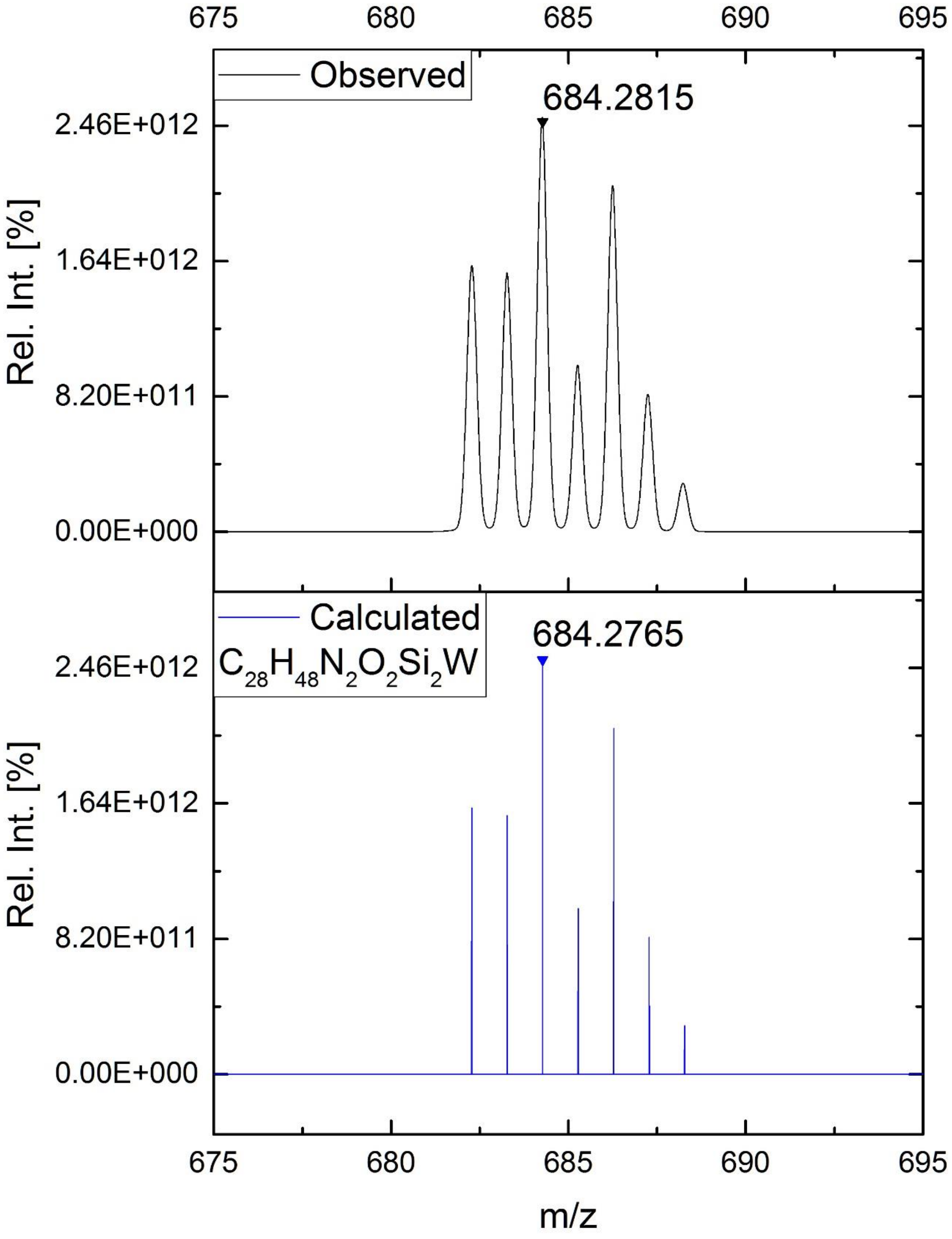

Figure S13. LIFDI-MS Spectrum: Expanded region of the compound signal showing the isotopic pattern of compound **3**. Observed (top) and calculated (bottom).

Synthesis of $Cp(CO)_2W$=Si(Si*t*Bu_3)(IMe_4) (3`):** To an NMR solution of compound **3** (25mg, 0.036 mmol) in 0.4 mL $C_6D_6$, $IMe_4$ (5 mg, 0.04 mmol, 1.1 equiv.) added. The $^1$H NMR spectra of reaction mixture was measured at room temperature after 30 minutes in which signals of **3** and **3` were observed in an approximately 40:60 ratio. After 12 hours a quantitative exchange was observed in ^{1}H NMR spectrum. Suitable crystals for single X-ray diffraction analysis were obtained by C_6D_6:pentane (1:1) mixture of compound **3`** at ambient temperature.

^{1}H NMR (400 MHz, C_6D_6, 298K): δ 5.48 (s, 5H, C_5H_5), 3.64 (s, 6H, N-CH_3), 1.34 (s, 6H, C-CH_3), 1.28 (s, 27H, ((CH_3)$_3$C)).

^{13}C NMR (101 MHz, C_6D_6, 298K): δ 235.48 (CO), 172.73 (:CN_2), 125.69 (CH_3C=CCH_3), 89.88 (C$_5H_5$), 32.27 (C(CH_3)$_3$), 31.68 (N-CH_3), 24.01 (C(CH_3)$_3$), 7.84 (CH_3C=CCH_3).

^{29}Si{^{1}H} NMR (80 MHz, C_6D_6, 298K): δ 231.14 (*Si*=W, $^1J_{WSi}$ = n.a.), 13.49 (*t*Bu_3*Si*)

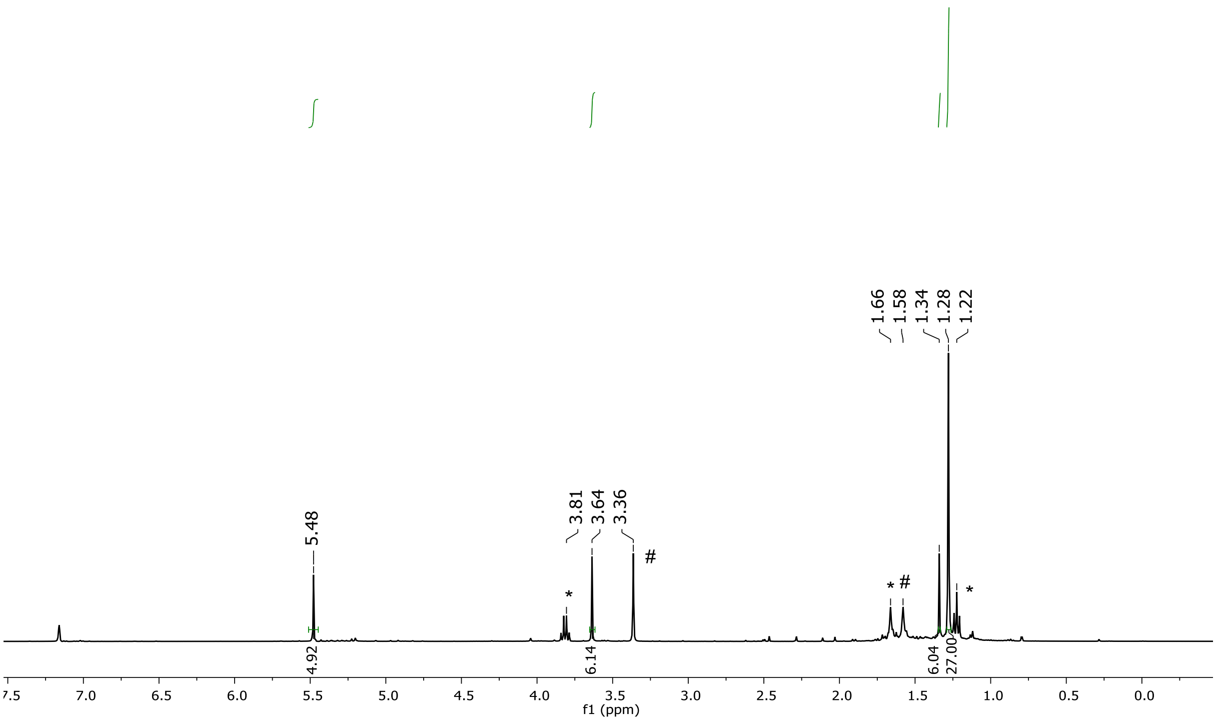

Figure S14. ^{1}H spectrum of compound **3`** in C_6D_6 at 298 K. (* = free IEt_2Me_2, # = Excess IMe_4)

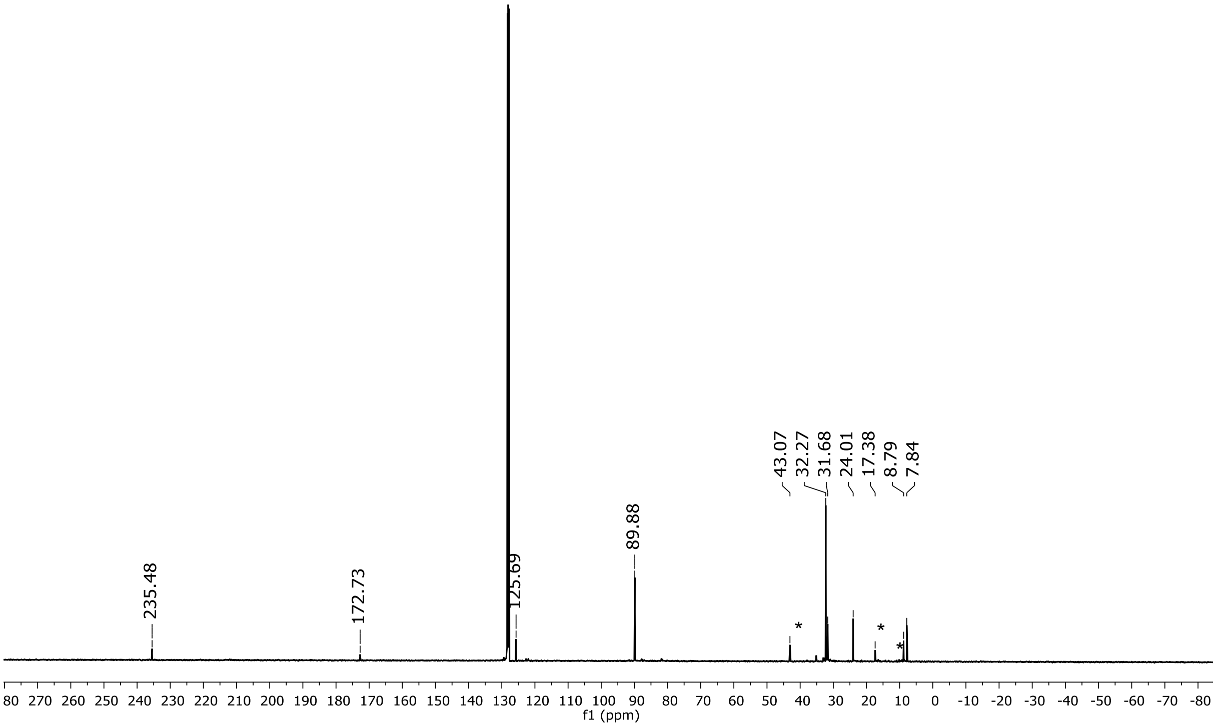

Figure S15. ^{13}C spectrum of compound **3`** in C_6D_6 at 298 K. (* = Free IEt_2Me_2)

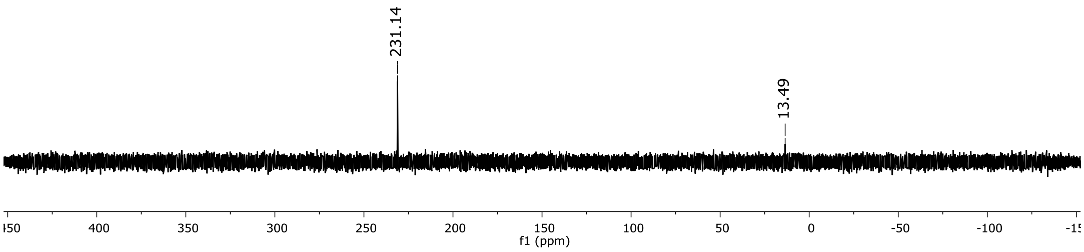

Figure S16. $^{29}Si\{^1H\}$ spectrum of compound **3`** in C_6D_6 at 298 K.

[Cp(CO)(Cl_3Al···OC)W=Si(Si*t*Bu_3)(IEt_2Me2) (4a) : To a toluene solution of **3** (50 mg, 0.073 mmol), $AlCl_3$ (10 mg, 0.073 mmol) added. Dark green solution turned immediately to dark red which was filtered from insoluble oily residue. Toluene was removed and **4a** was obtained as sticky dark red solid in 64% yield. Suitable crystals for single X-ray diffraction analysis were obtained by concentrated toluene solution of **4a** at 3 ^oC.

^1H NMR (400 MHz, C_6D_6, 298K): δ 5.28 (s, 5H, C_5H_5), 3.97 (m, 1H, N-CH_2CH_3), 3.88 (m, 1H, N-CH_2CH_3), 3.49 (m, 1H, N-CH_2CH_3), 3.32 (m, 1H, N-CH_2CH_3), 1.76 (s, 3H, C-CH_3), 1.74 (s, 3H, C-CH_3), 1.14 (s, 27H, (($CH_3)_3$C)), 1.36 (t, 6H, N-CH_2CH_3).

^{13}C NMR (101 MHz, C_6D_6, 298K): δ 220.22 (CO), 166.86 (:CN_2), 126.82 (CH_3C=CCH_3), 91.51 (C_5H_5), 43.55 (N_a-CH_2CH_3), 41.85 (N_b-CH_2CH_3). 32.14 ($C(CH_3)_3$), 24.80 ($C(CH_3)_3$), 15.15 (N_a-CH_2CH_3), 13.21 (N_b-CH_2CH_3), 8.75 (CH_3C=CCH_3), 8.40 (CH_3C=CCH_3).

$^{29}Si\{^1H\}$ NMR (80 MHz, C_6D_6, 298K): δ 322.03 (*Si*=W, $^1J_{WSi}$ = n.a.), 15.59 (*t*Bu_3*Si*)

^{29}Si INEPT NMR (80 MHz, C_6D_6, 298K): δ 15.57 (*t*Bu_3*Si*)

IR (ATR, neat) [cm^{-1}]: υ(CO) = 1813, 1901

EA: Due to presence of undefined oily impurities, satisfactory elemental analysis results were not obtained.

[Cp(CO)(($C_6F_5)_3$B···OC)W=Si(Si*t*Bu_3)(IEt_2Me2) (4b): Compound **4b** was synthesized in similar manner as **4a**. Unfortunately, we could not isolate **4b** due to its oily nature hence we could not obtain pure NMR spectra except ^{11}B and ^{29}Si.

^{11}B NMR (128 MHz, C_6D_6, 298K): δ – 14.53

$^{29}Si\{^1H\}$ NMR (80 MHz, C_6D_6, 298K): δ 323.15 (*Si*=W, $^1J_{WSi}$ = n.a.), 17.06 (*t*Bu_3*Si*).

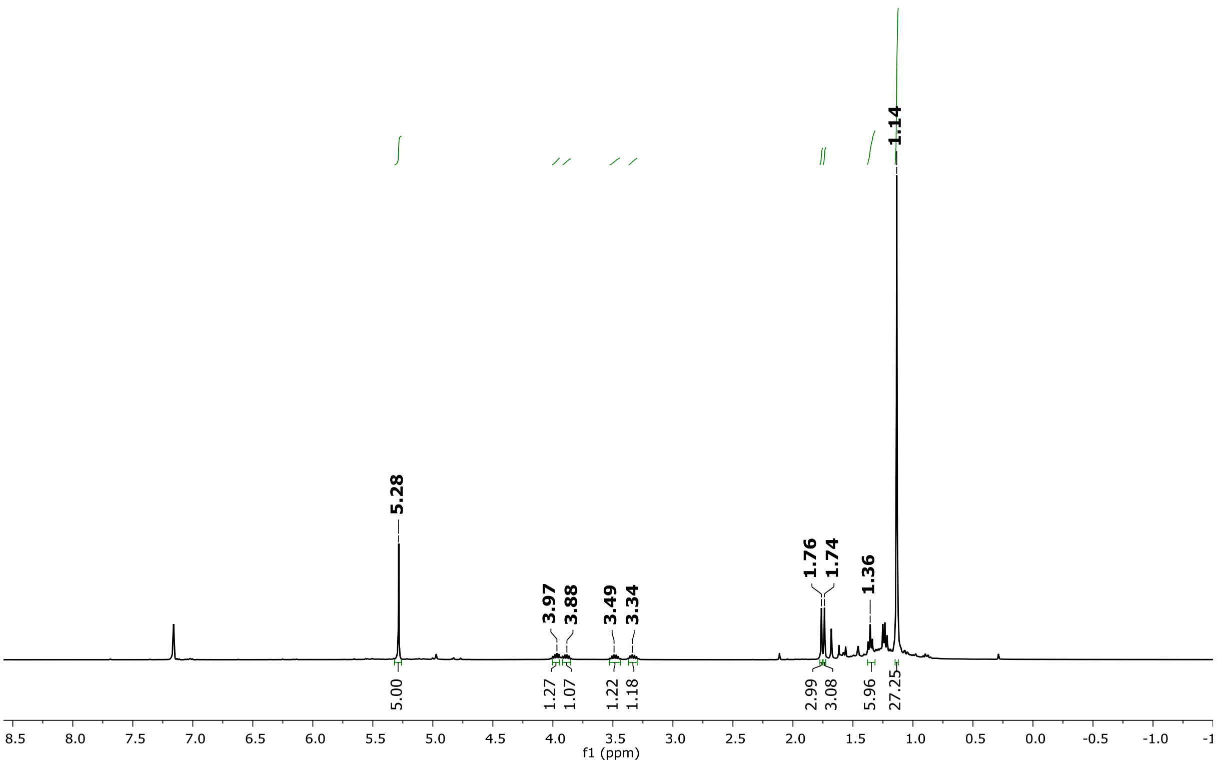

Figure S17. ^{1}H spectrum of compound **4a** in C_6D_6 at 298 K.

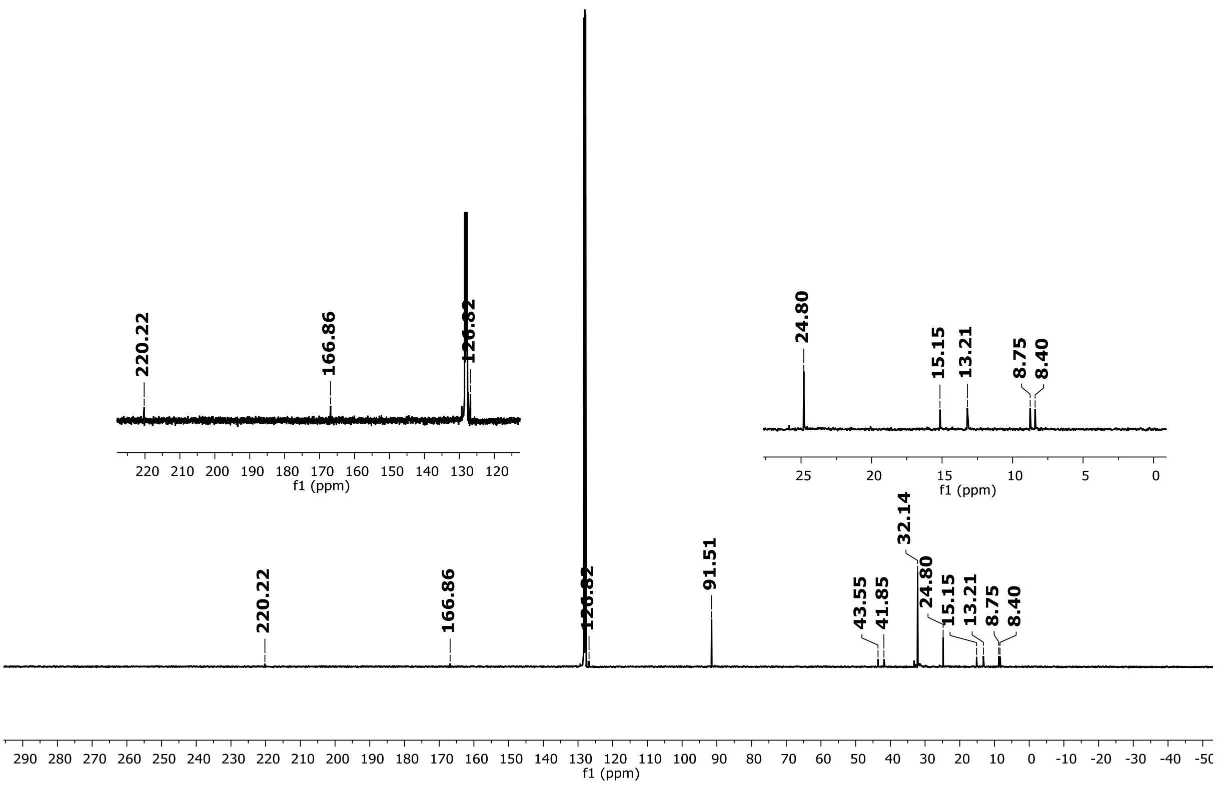

Figure S18. ^{13}C spectrum of compound **4a** in C_6D_6 at 298 K.

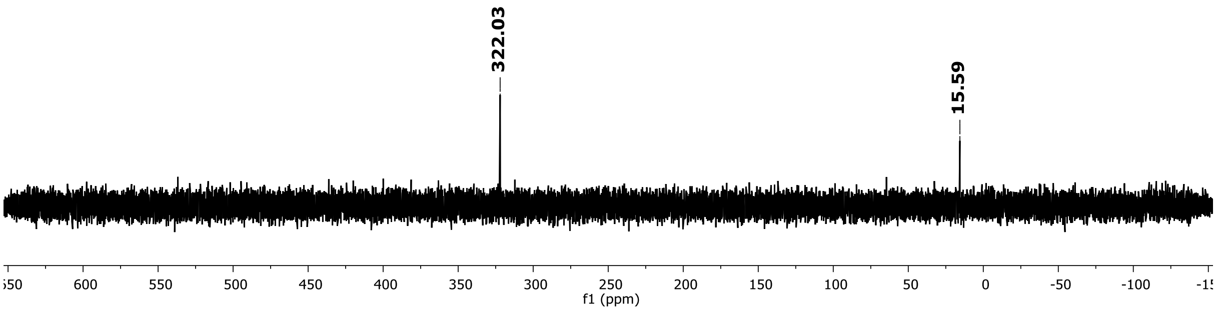

Figure S19. $^{29}Si\{^{1}H\}$ spectrum of compound **4a** in C_6D_6 at 298 K.

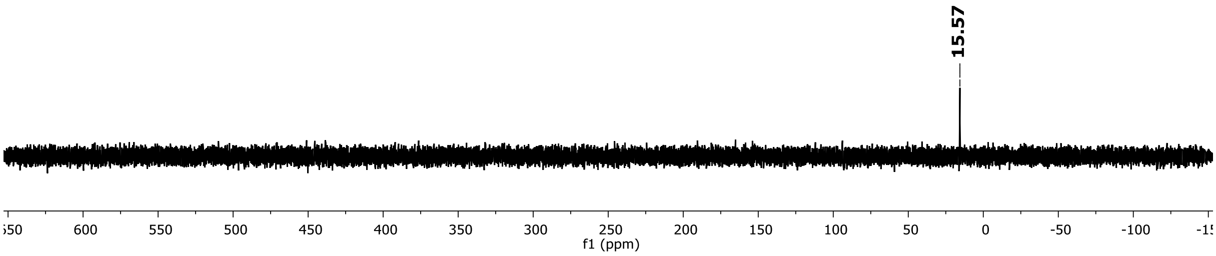

Figure S20. ^{29}Si−INEPT spectrum of compound **4a** in C_6D_6 at 298 K.

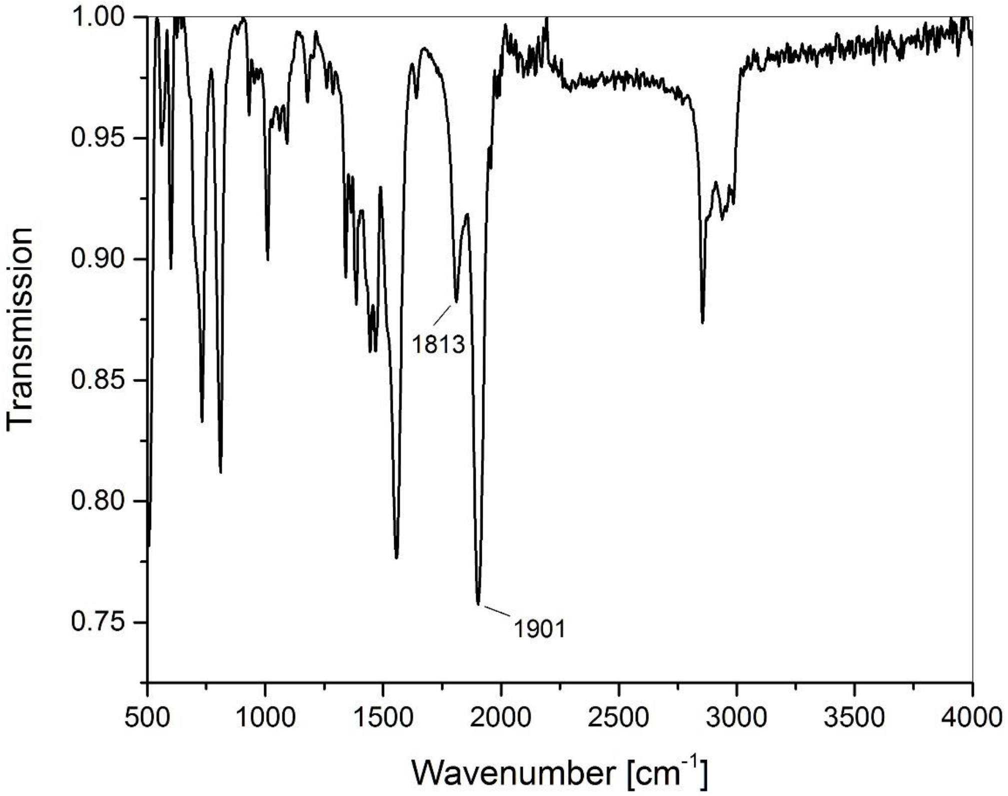

Figure S21. IR Spectrum of compound **4a**. (ATR, neat)

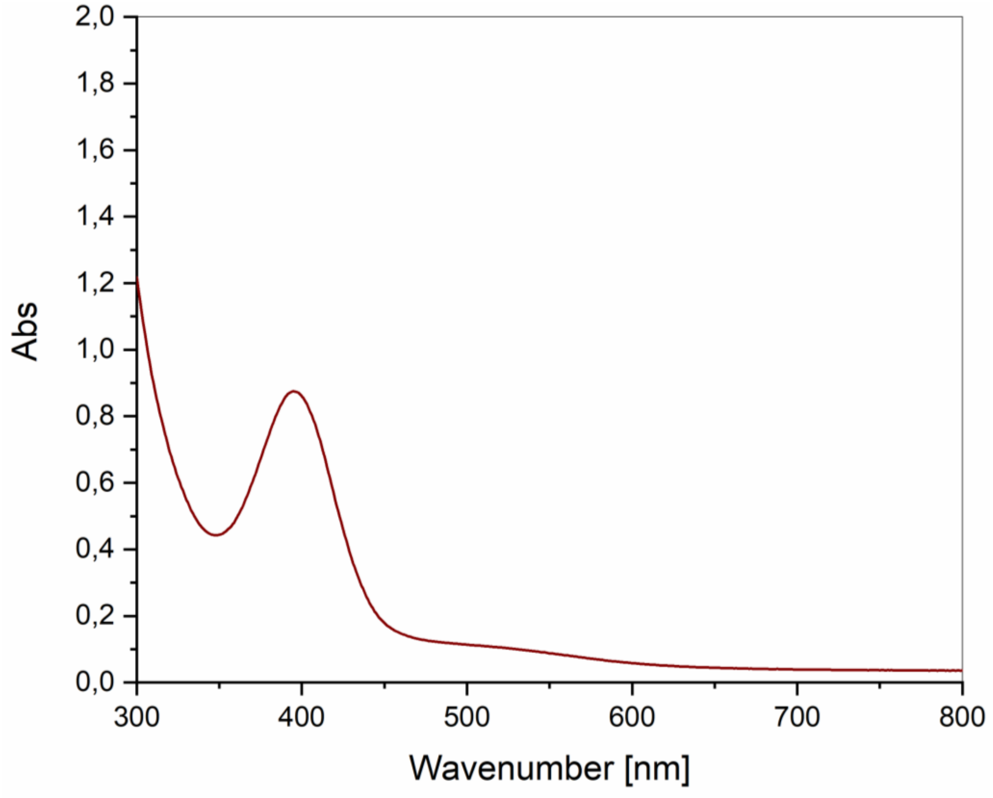

Figure S22. UV-Vis spectra of compound **4a** in toluene. (Conc. 1.17 x 10^{-4}M; ε_{395}= 7474 L mol $^{-1}$cm^{-1})

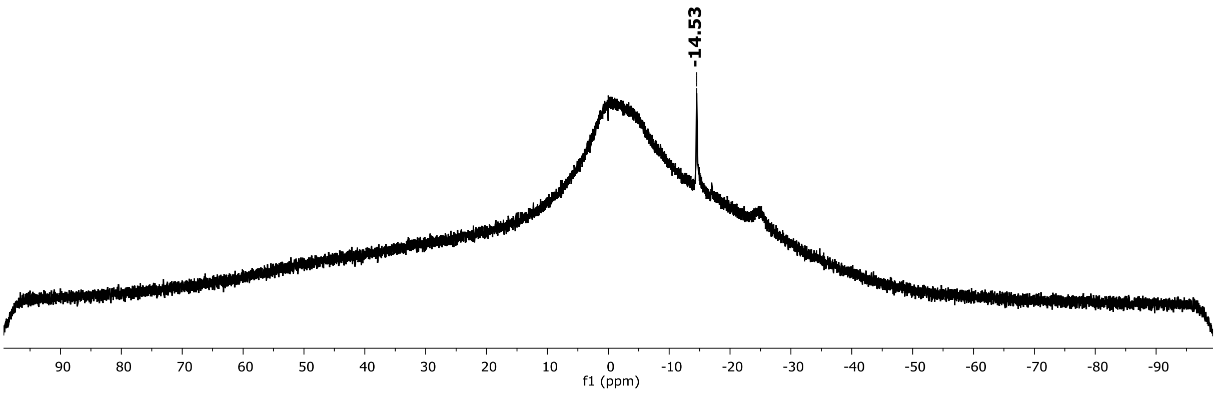

Figure S23. ^{11}B spectrum of compound **4b** in C_6D_6 at 298 K.

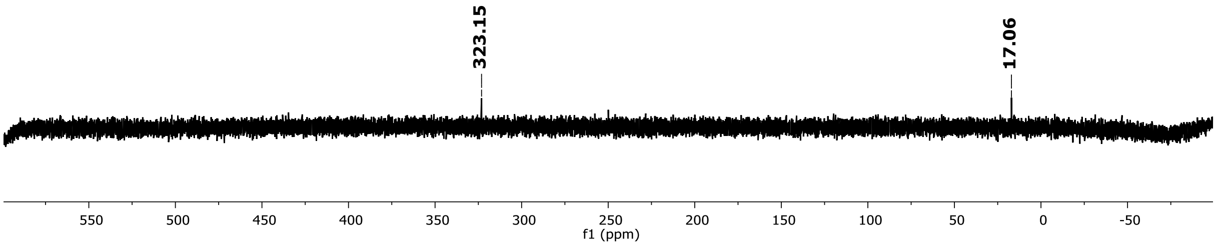

Figure S24. $^{29}Si\{^1H\}$ spectrum of compound **4b** in C_6D_6 at 298 K.

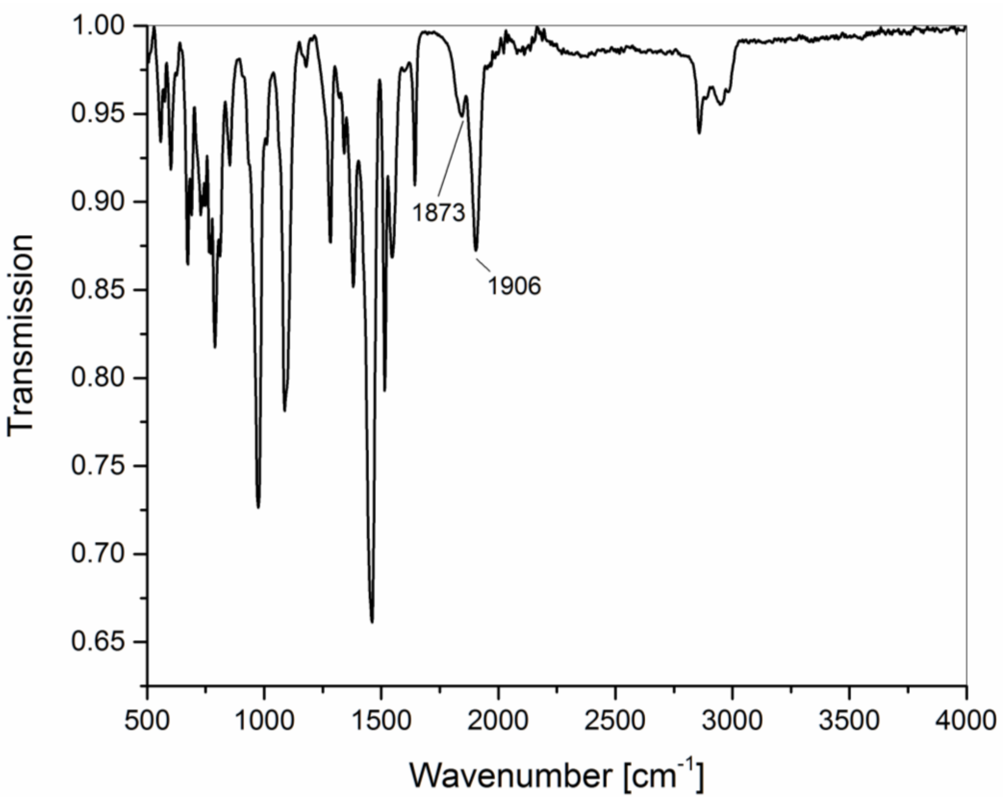

Figure S25. IR Spectrum of compound **4b**. (ATR, neat)

[Cp(CO)$_2$MoSi(Si*t*Bu$_3$)]$_2$ (5): Compound **2** (250 mg, 0.42 mmol) and BPh_3 (102 mg, 0.42 mmol) were dissolved in 10 mL toluene and heated to 90 ˚C. After 30 minutes the dark green solution turned into purple-brown solution. Toluene was removed to yield purple waxy residue and 15 mL pentane was added (upon pentane addition, beige solid precipitated, which was later characterized as BPh_3·IEt_2Me_2). Suspension placed at -80 ˚C cold bath for 30 minutes and purple solution was filtered at cold. Added 5 mL pentane to the beige solid, stirred 10 minutes, placed in cold bath again for 40 minutes and filtered again. Pentane was evaporated to yield compound **5** as a purple solid. (75 mg, 40%)

^{1}H NMR (400 MHz, C_6D_6, 298K): δ 4.72 (s, 5H, C_5H_5), 1.43 (s, 27H, ((CH_3)$_3$C)).

^{13}C NMR (101 MHz, C_6D_6, 298K): δ 238.09 (*C*O), 230.37 (*C*O), 86.50 (C_5H_5), 32.77 (*C*(CH_3)$_3$), 25.96 (C(*C*H_3)$_3$).

^{29}Si{^{1}H} NMR (79 MHz, C_6D_6, 298K): δ 3.65 (SiMo), 48.32 (*t*Bu$_3$*Si*)

^{29}Si INEPT NMR (79 MHz, C_6D_6, 298K): δ 48.52 (*t*Bu$_3$*Si*)

IR (ATR, neat) [cm^{-1}]: υ(CO) = 1844, 1918

LIFDI-MS [m/z]: calculated (for $C_{38}H_{64}O_4Si_4Mo_2$): 889.2004, observed: 889.1627

M.P.: 64–65 °C

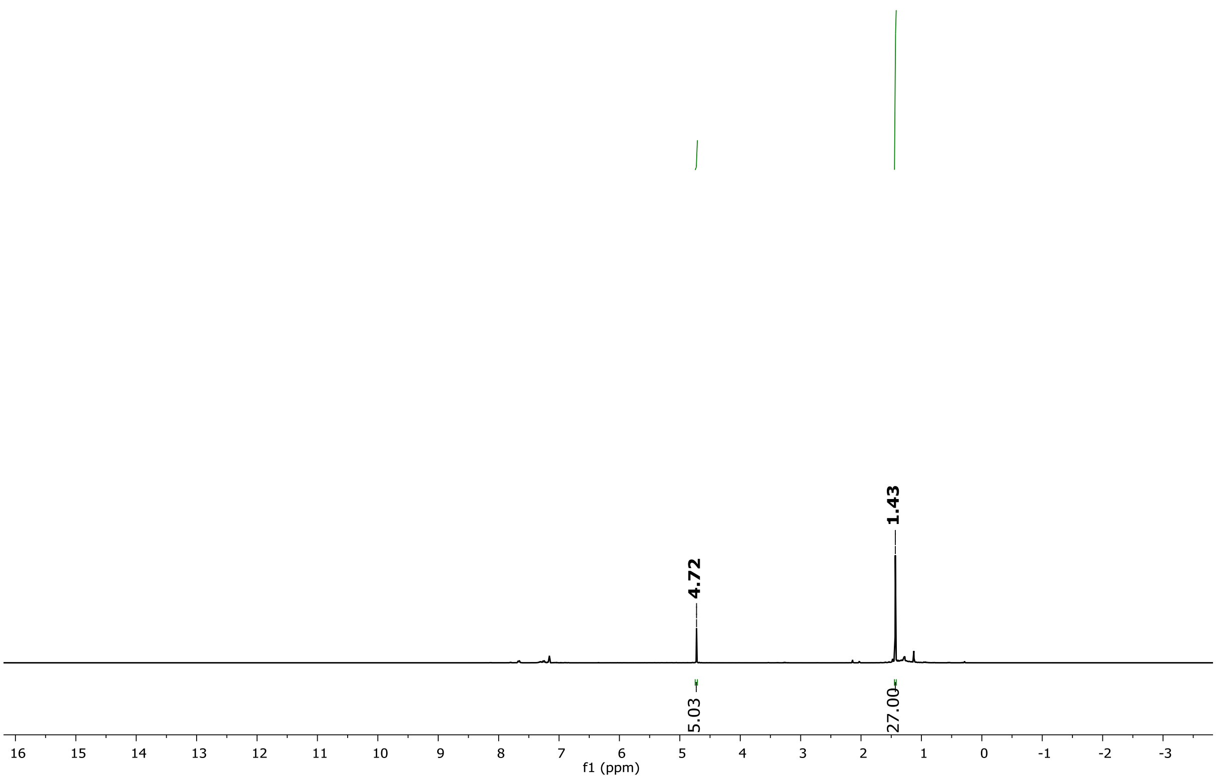

Figure S26. ^{1}H spectrum of compound **5** in C_6D_6 at 298 K.

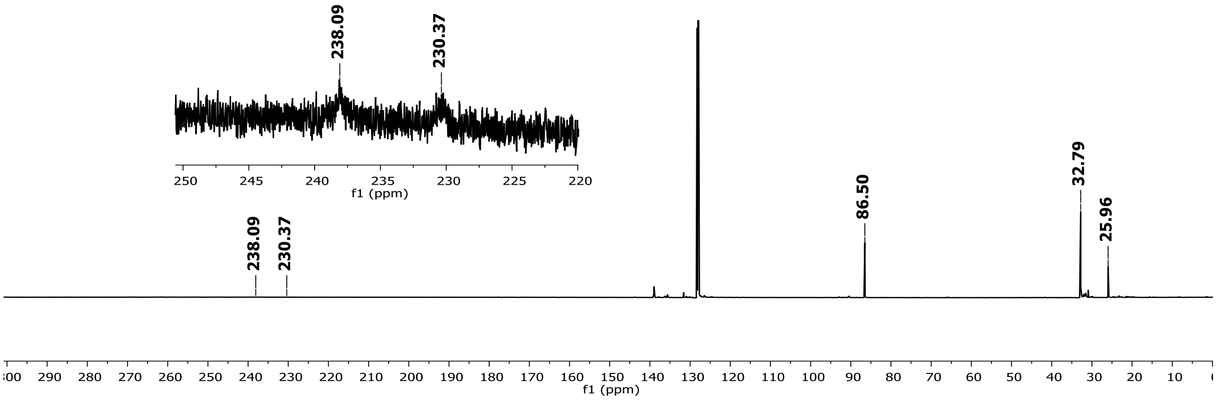

Figure S27. ^{13}C spectrum of compound **5** in C_6D_6 at 298 K.

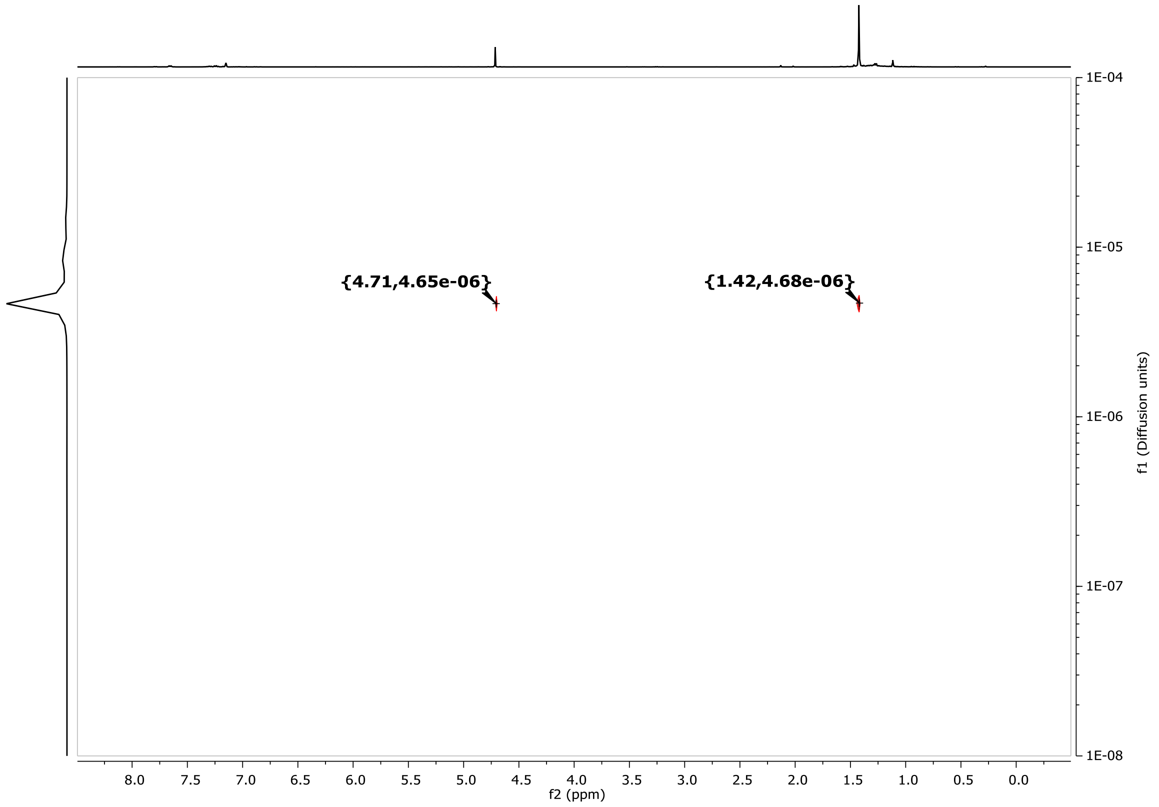

Figure S28. ^{1}H-2D DOSY NMR spectrum of compound **5** in C_6D_6 at 298 K.

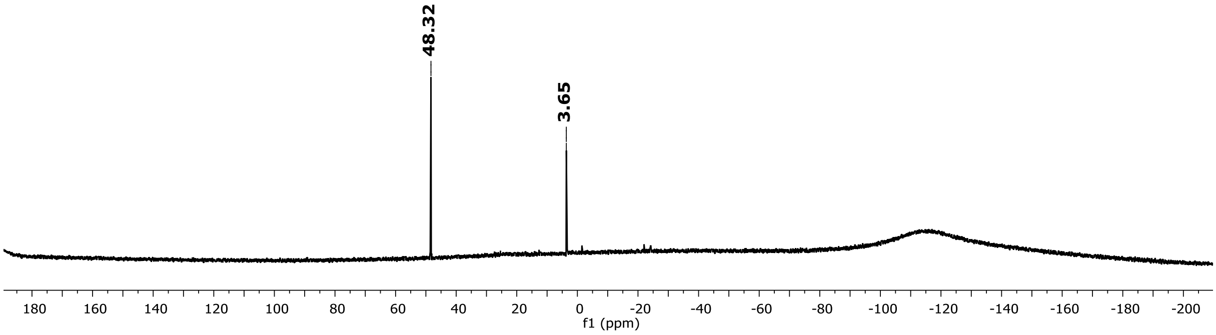

Figure S29. $^{29}Si\{^{1}H\}$ NMR spectrum of compound **5** in C_6D_6 at 298 K.

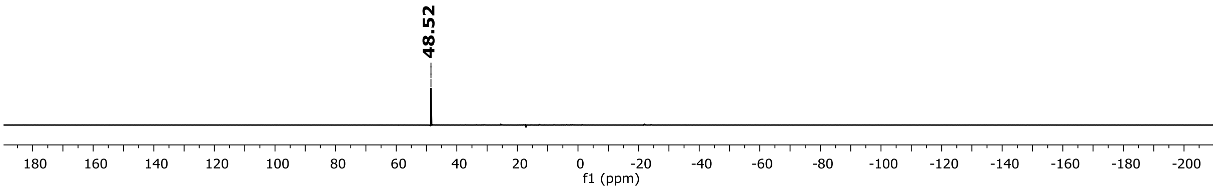

Figure S30. ^{29}Si–INEPT NMR spectrum of compound **5** in C_6D_6 at 298 K.

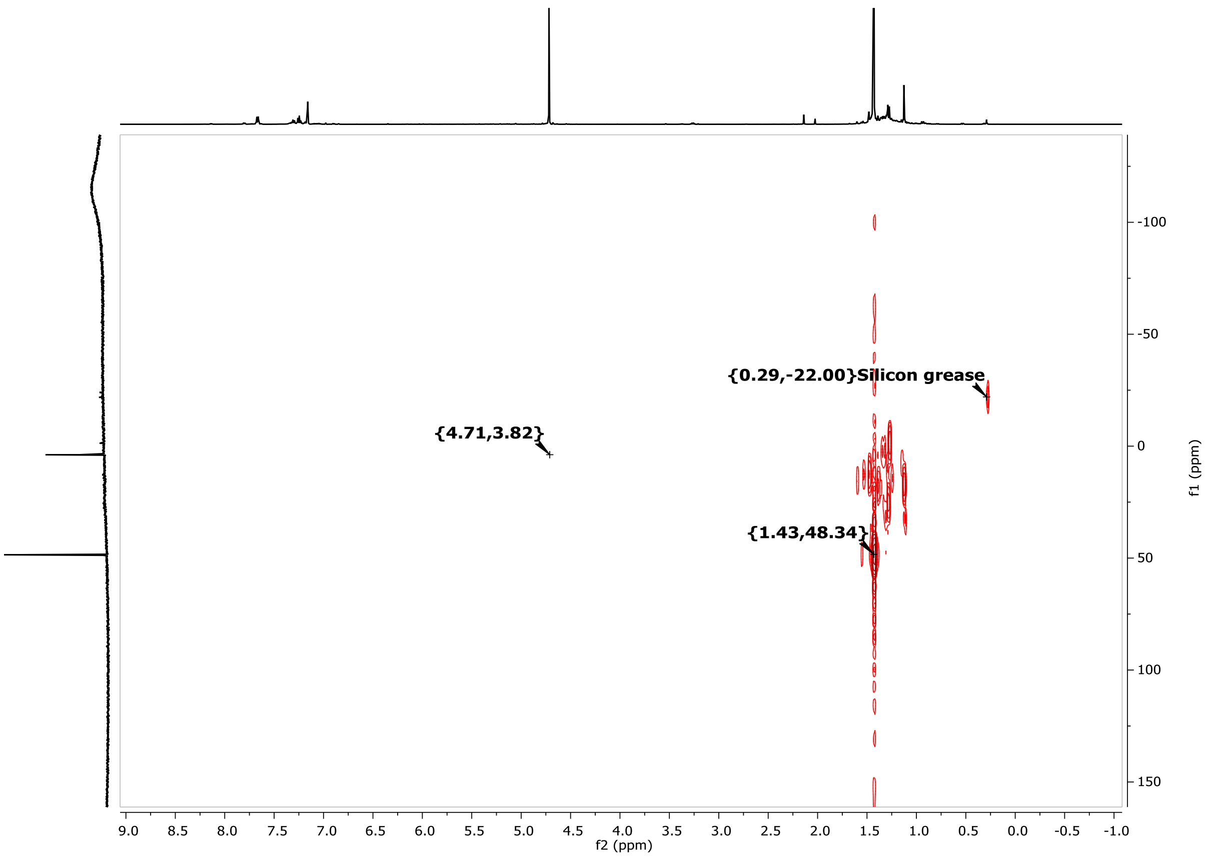

Figure S31. ^{1}H-^{29}Si HMBC NMR spectrum of compound **5** in C_6D_6 at 298 K.

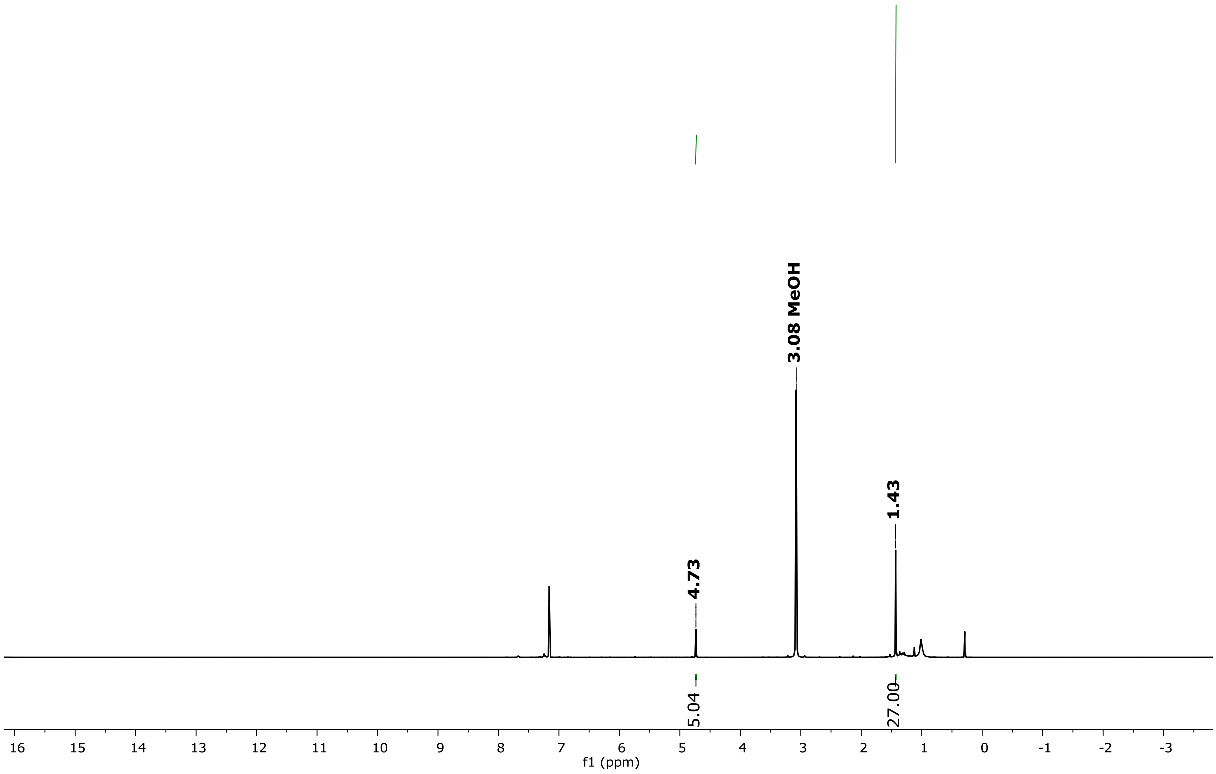

Figure S32. ^{1}H NMR spectrum of compound **5** upon treatment of excess MeOH in C_6D_6 at 298 K.

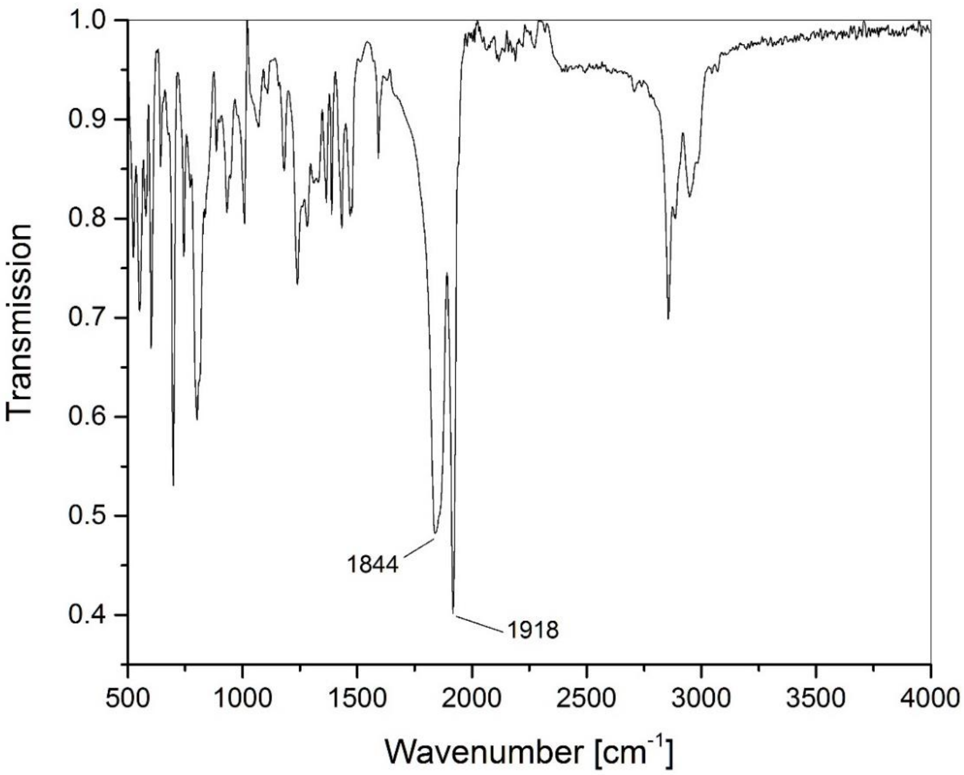

Figure S33. IR Spectrum of compound **5**. (ATR, neat)

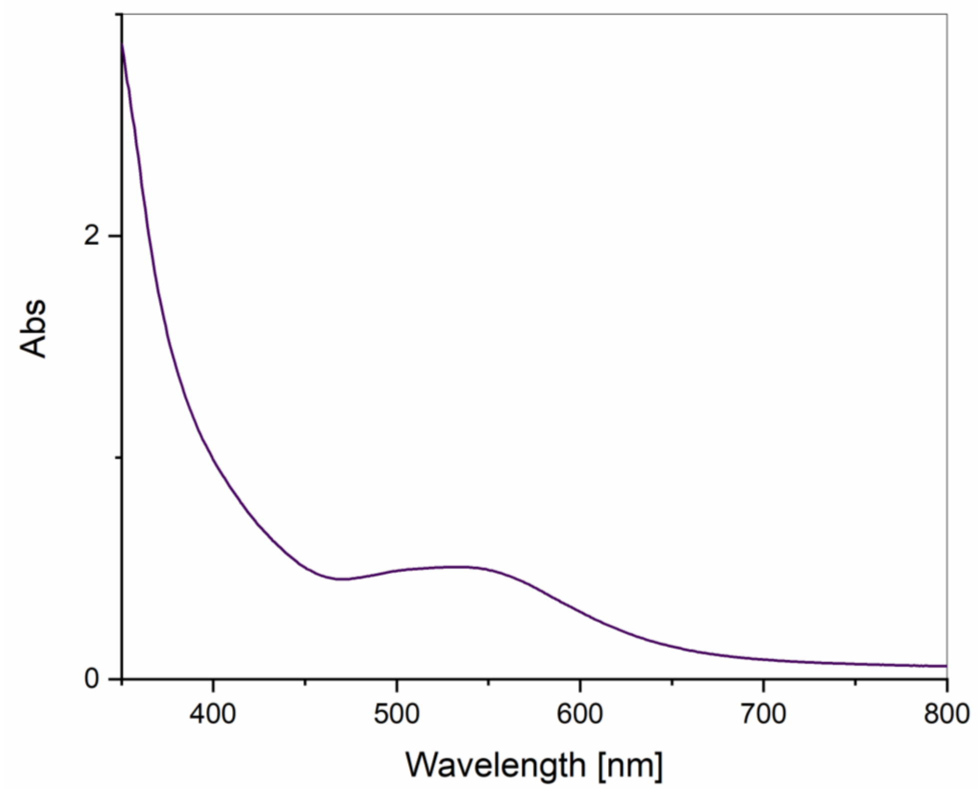

Figure S34. UV-Vis Spectra of compound **5** in toluene at 298 K. (Conc. 5.625 x 10^{-4}M; ε_{543} = 894 L $mol^{-1}cm^{-1}$)

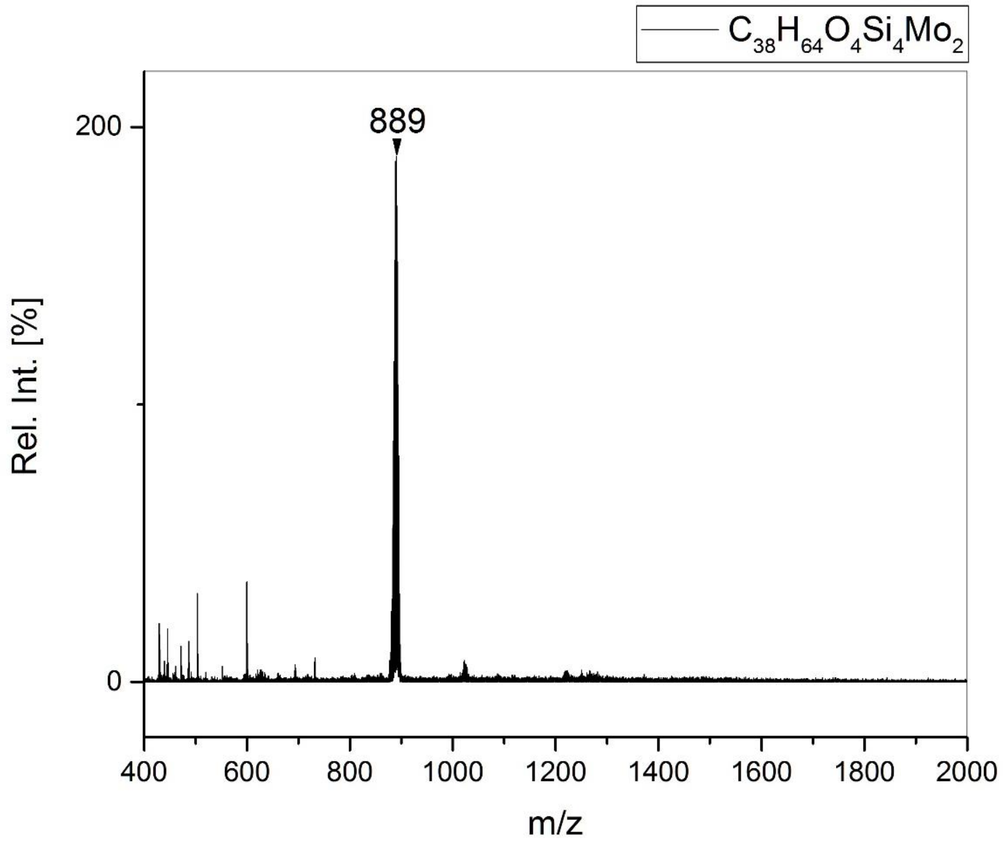

Figure S35. LIFDI-MS spectrum of compound **5** (in toluene solution). Compound observed at m/z = 889.

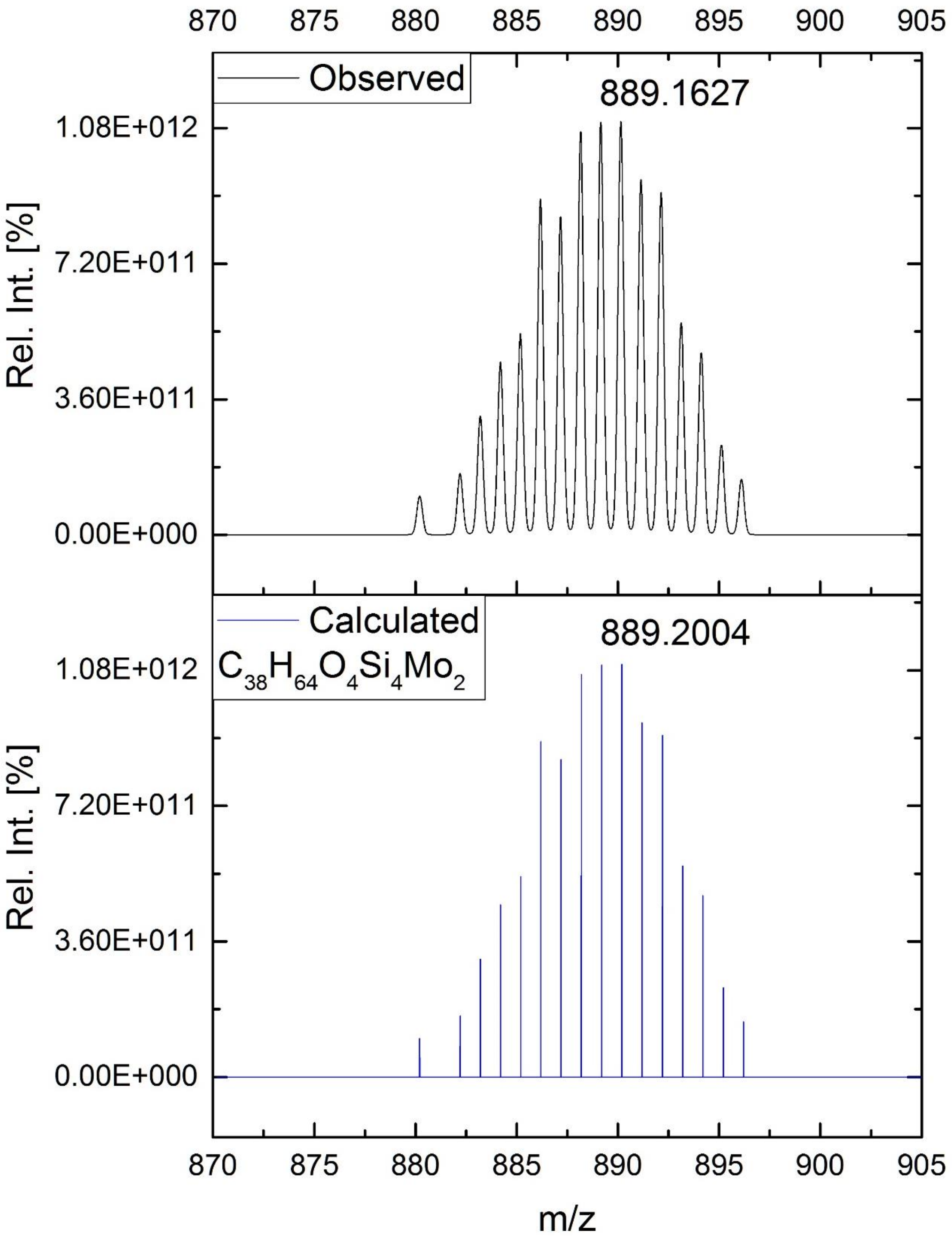

Figure S36. LIFDI-MS Spectrum: expanded region of the product signal illustrating the isotopic pattern of compound **5**. Observed (top) and calculated (bottom).

[Cp(CO)$_2$WSi(Si*t*Bu$_3$)]$_2$ (6): Compound **3** (99 mg, 0.14 mmol) and BPh_3 (35 mg, 0.14 mmol) were dissolved in 8 mL toluene and heated to 90 ˚C. After 30 minutes the dark green solution turned into dark red. Toluene was removed to yield red waxy residue and 15 mL pentane was added (upon pentane addition, beige solid precipitated, which was later characterized as BPh_3·IEt$_2$Me$_2$). Suspension placed at −80 ˚C cold bath for 30 minutes and red solution was filtered. Added 5 mL pentane to the beige solid, stirred 10 minutes, placed in cold bath again for 40 minutes and filtered again. Pentane was evaporated to yield compound **6** as a red solid. (40 mg, 52%). Suitable crystals for single X-ray diffraction analysis were obtained by concentrated hexane solution of **6** at ambient temperature.

^{1}H NMR (400 MHz, C_6D_6, 298K): δ 4.70 (s, 5H, C_5*H*$_5$), 1.44 (s, 27H, ((C*H*$_3$)$_3$C)).

^{13}C NMR (101 MHz, C_6D_6, 298K): δ 225.38 (*C*O), 215.11 (*C*O), 83.99 (*C*$_5$H$_5$), 32.85 (*C*(CH$_3$)$_3$), 25.82 (C(*C*H$_3$)$_3$).

^{29}Si{^{1}H} NMR (79 MHz, C_6D_6, 298K): δ −63.04 (SiW, $^{1}J_{WSi}$ = 52.07 Hz), 43.99 (*t*Bu$_3$*Si*)

^{29}Si INEPT NMR (79 MHz, C_6D_6, 298K): δ 44.10 (*t*Bu$_3$*Si*)

IR (ATR, neat) [cm^{-1}]: υ(CO) = 1860, 1914

LIFDI-MS [m/z]: calculated (for $C_{38}H_{64}O_4Si_4W_2$): 1064.2903, observed: 1064.3159

M.P.: 67−69 ºC

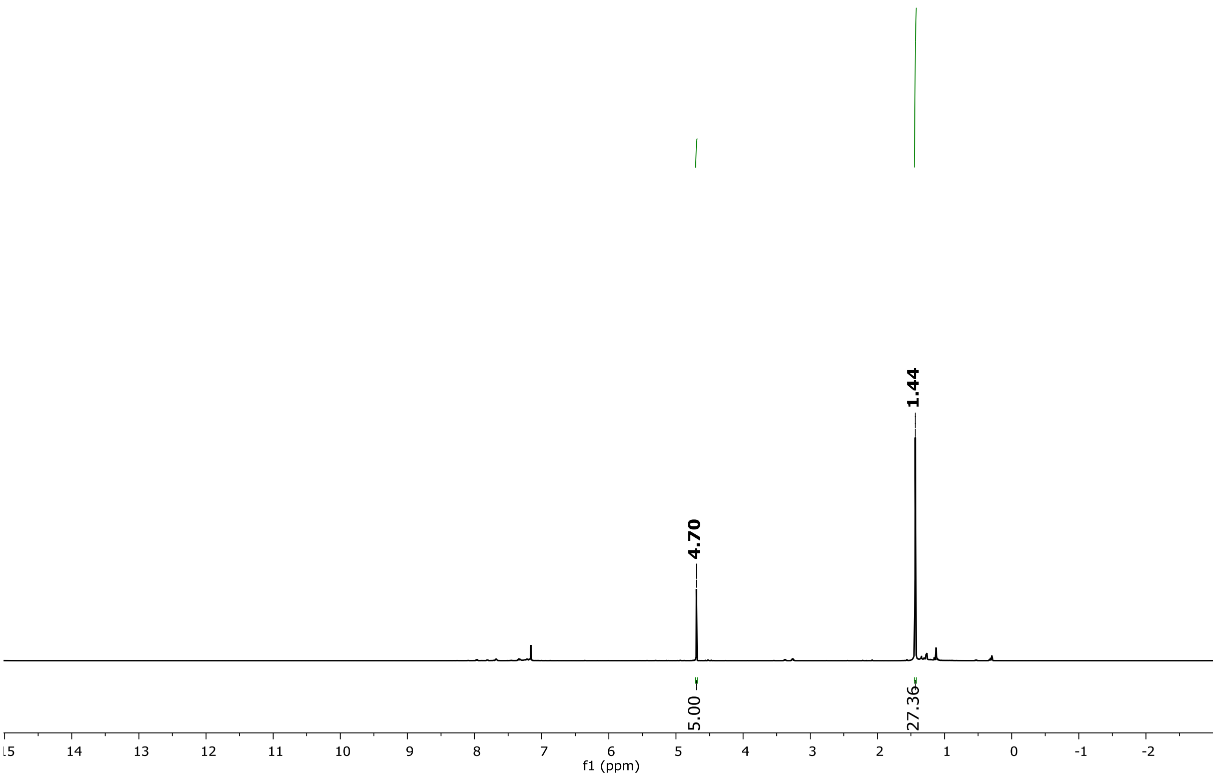

Figure S37. ^{1}H NMR spectrum of compound **6** in C_6D_6 at 298 K.

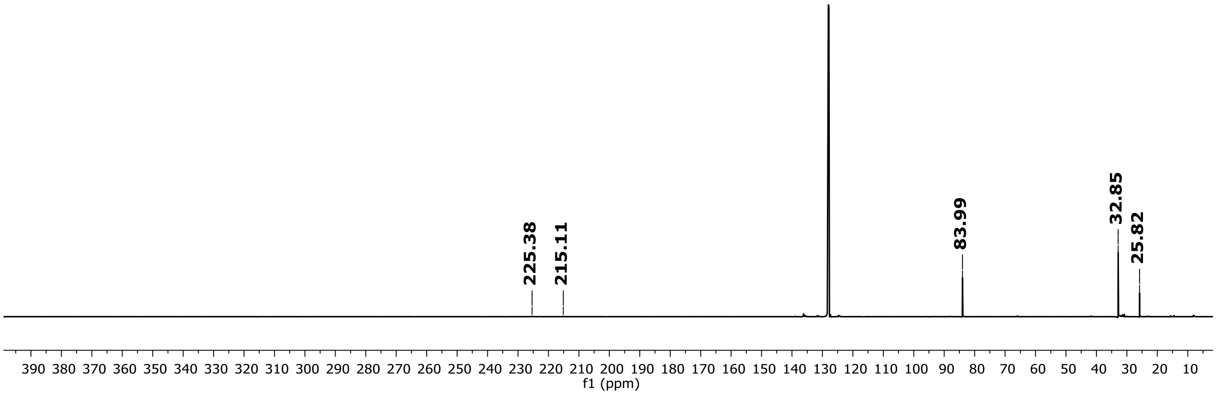

Figure S38. ^{13}C NMR spectrum of compound **6** in C_6D_6 at 298 K.

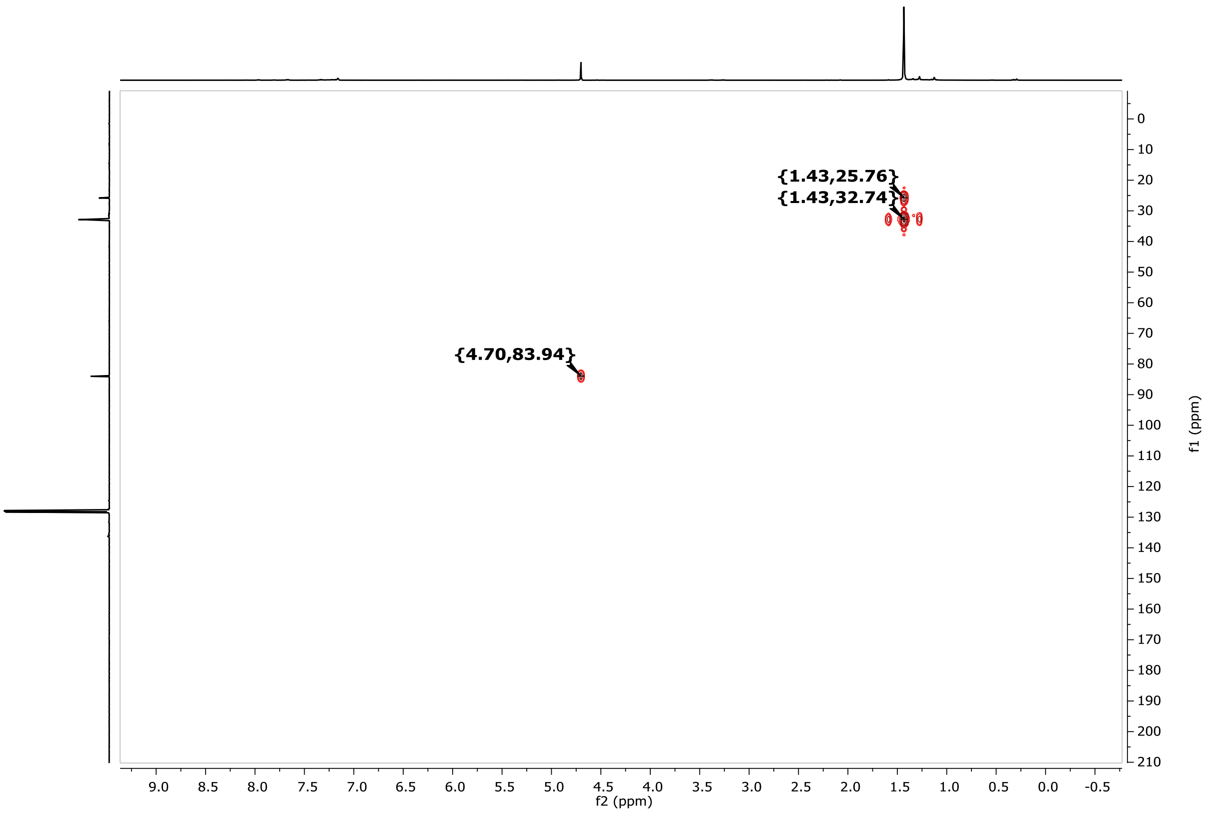

Figure S39. ^{1}H-^{13}C HMBC NMR spectrum of compound **6** in C_6D_6 at 298 K.

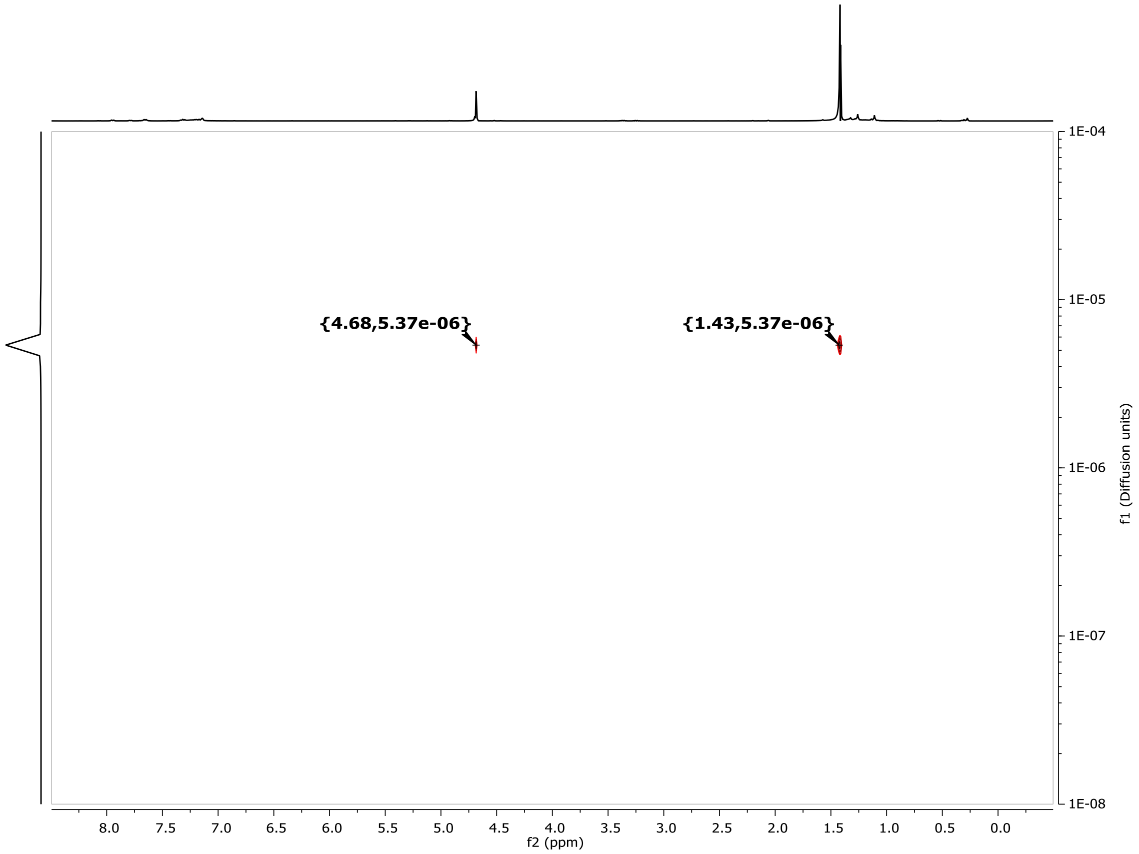

Figure S40. ^{1}H-2D DOSY NMR spectrum of compound **6** in C_6D_6 at 298 K.

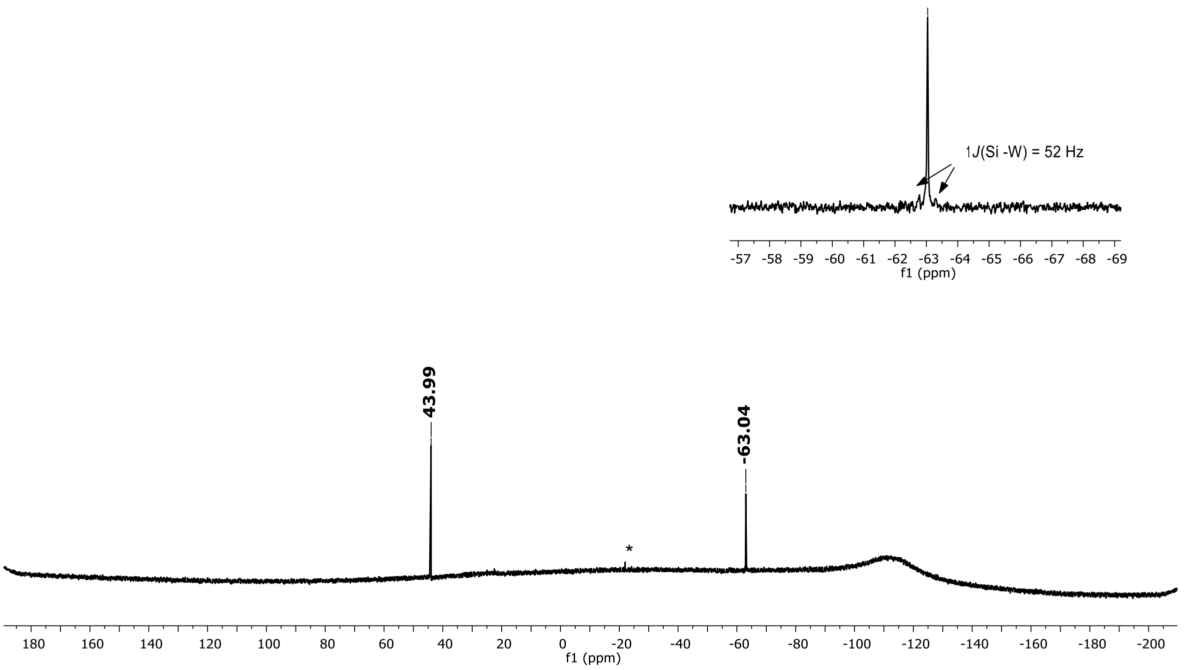

Figure S41. $^{29}Si\{^1H\}$ NMR spectrum of compound **6** in C_6D_6 at 298 K. (* = silicon grease)

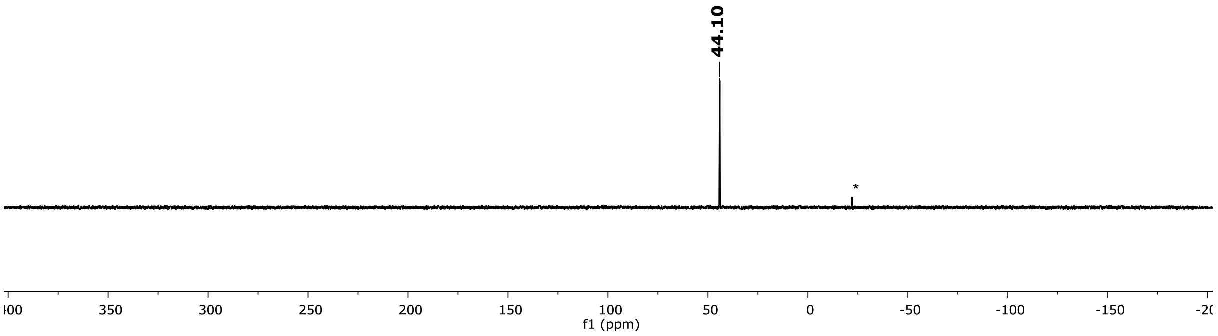

Figure S42. ^{29}Si-INEPT NMR spectrum of compound **6** in C_6D_6 at 298 K. (* = silicon grease)

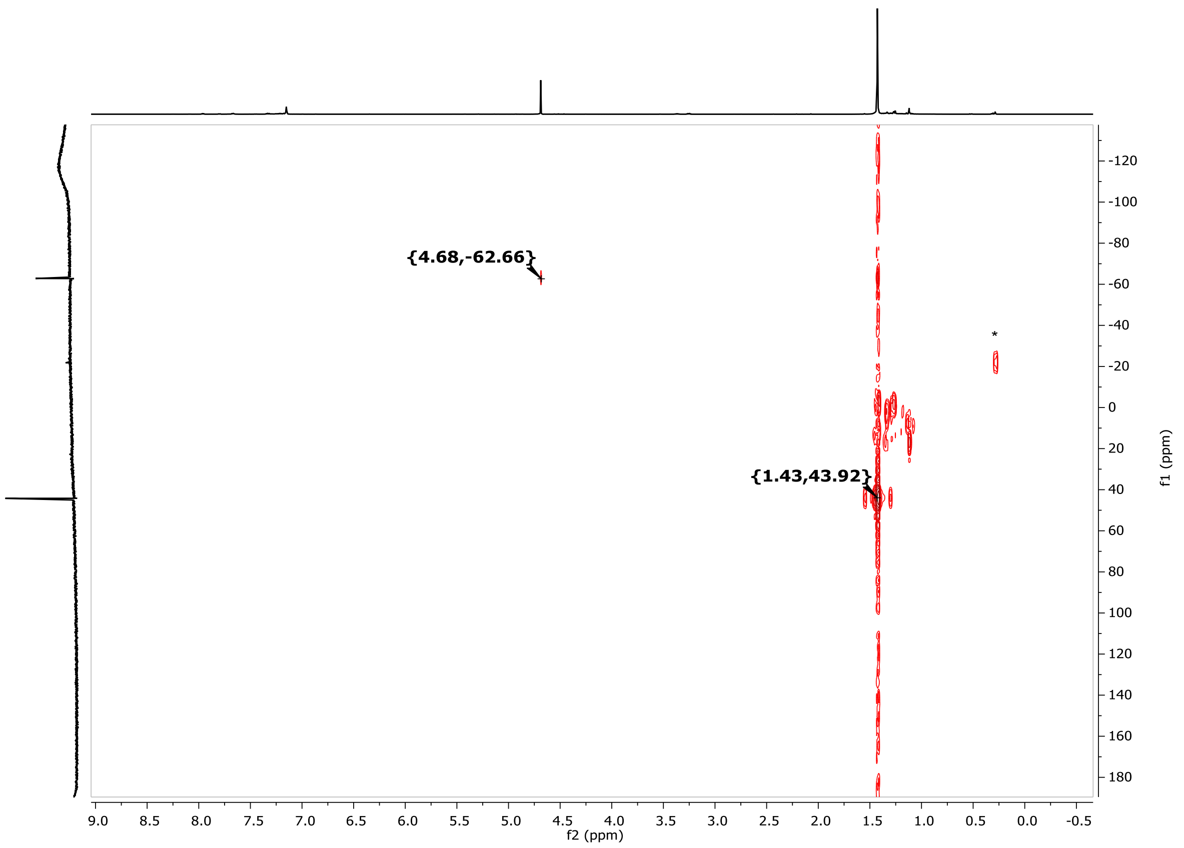

Figure S43. ^{29}Si-^{1}H HMBC NMR spectrum of compound **6** in C_6D_6 at 298 K.

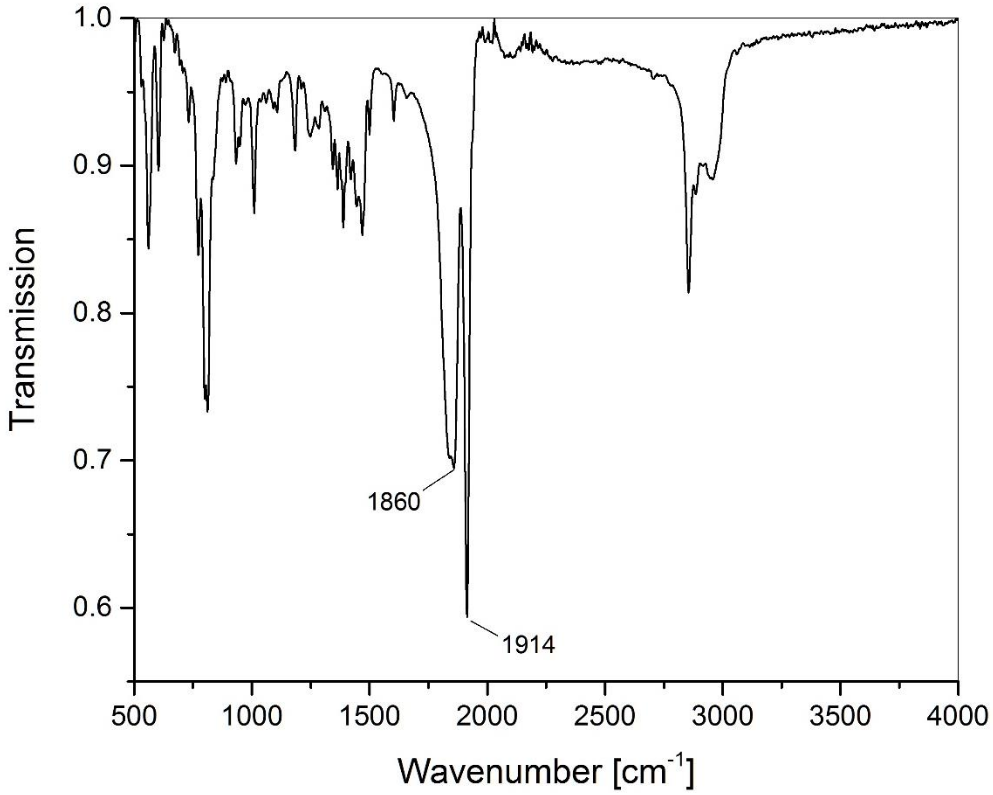

Figure S44. IR Spectrum of compound **6**. (ATR, neat)

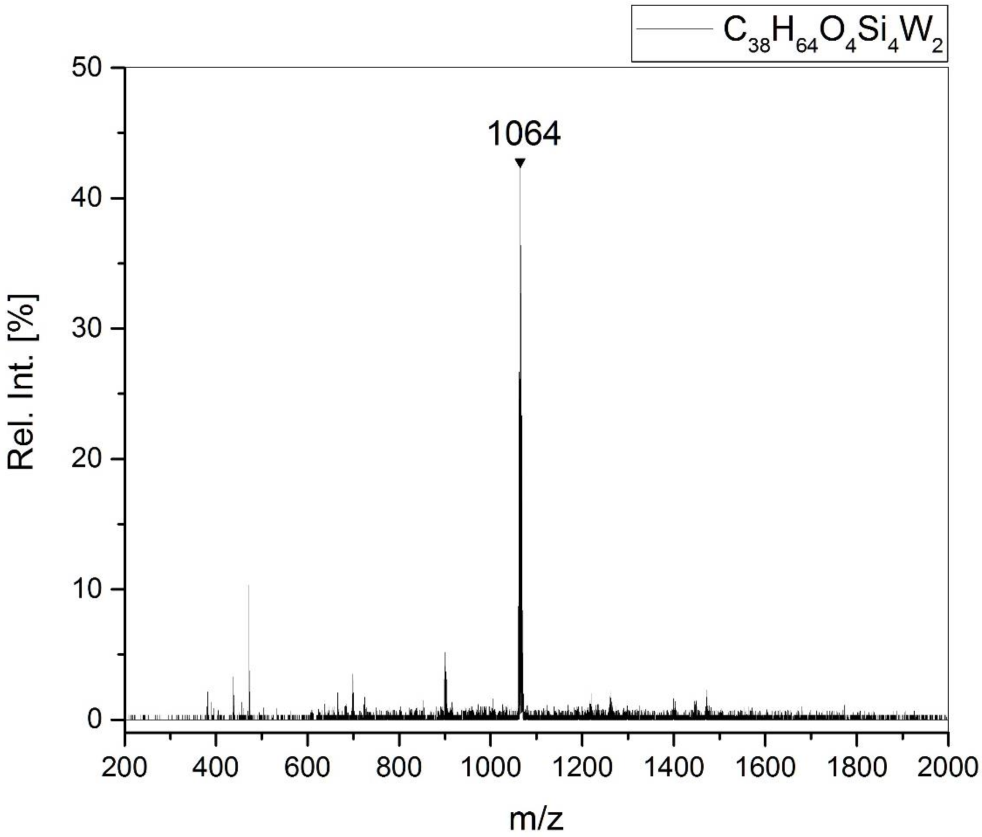

Figure S45. LIFDI-MS spectrum of compound **6** (in toluene solution). Compound observed at m/z = 1064.

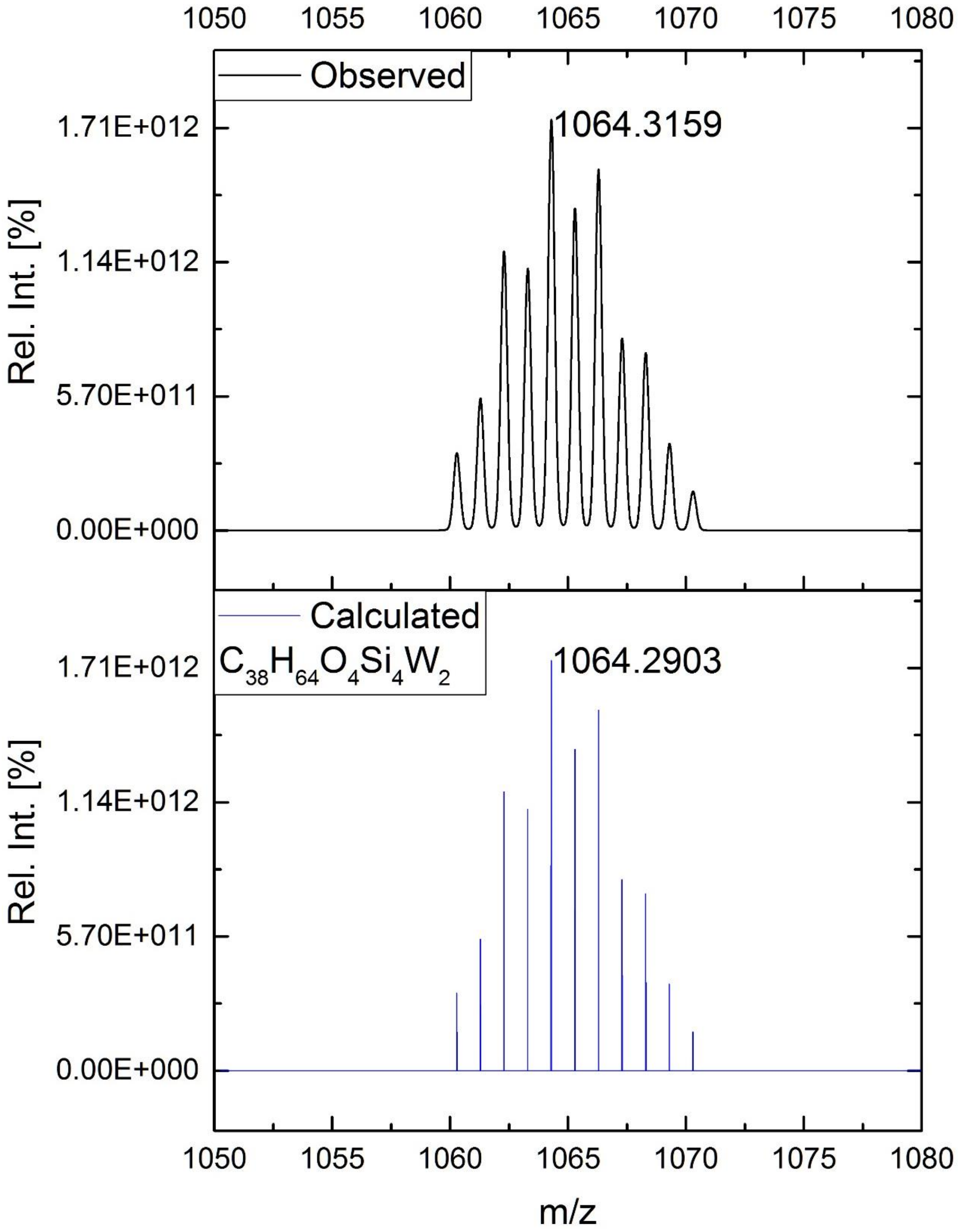

Figure S46. LIFDI-MS Spectrum: expanded region of the product signal illustrating the isotopic pattern of compound **6**. Observed (top) and calculated (bottom).

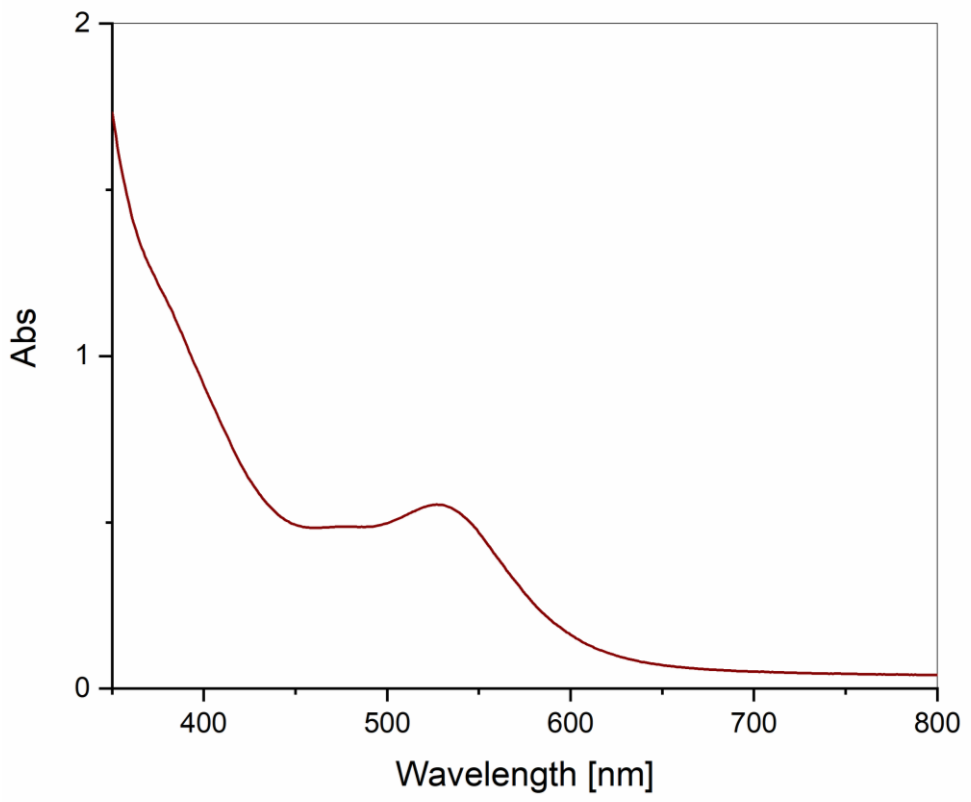

Figure S47. UV-Vis Spectra of compound **6** in toluene at 298 K. (Conc. 4.695 x 10^{-4}M; ε_{530} = 1178 L $mol^{-1}cm^{-1}$)

Isolation of IEt_2Me_2·BPh_3 (beige precipitate that collected from synthesis of compound **6**)

^{1}H NMR (400 MHz, C_6D_6, 298K): δ 7.69 (d, 6H, *o*-C_6H_5), 7.34 (t, 6H, *m*-C_6H_5), 7.21 (t, 3H, *p*-C_6H_5), 3.38 (q, 4H, N-CH_2CH_3), 1.28 (s, 6H, CH_3C=CCH_3), 0.33 (t, 6H, N-CH_2CH_3).

^{11}B NMR (128 MHz, C_6D_6, 298K): −8.53 (br, $w_{½}$ = 46.8 Hz)

^{13}C NMR (101 MHz, C_6D_6, 298K): 169.25[a] (N_2C:BPh_3, IEt_2Me_2), 157.71[a] (B-C_6H_5), 136.2 (*o*-C_6H_5), 127.33 (*m*-C_6H_5), 124.70 (*p*-C_6H_5), 124.29 (CH_3C=CCH_3), 41.77 (N-CH_2CH_3), 14.59 (N-CH_2CH_3), 8.21 (CH_3C=CCH_3).

a: The signals were not observed in ^{13}C-1D NMR spectra but found in the 2D ^{1}H^{13}C HMBC experiment.

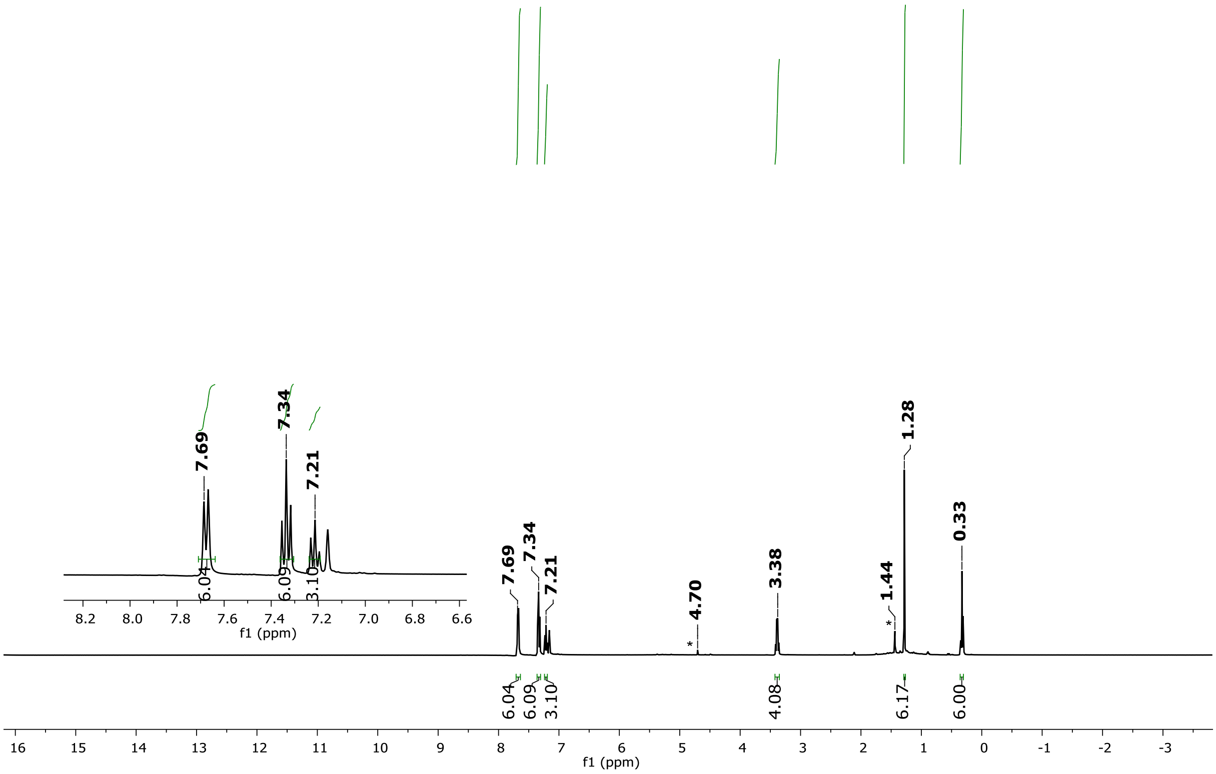

Figure S48. ^{1}H NMR spectrum of $BPh_3{\cdot}IEt_2Me_2$ in C_6D_6 at 298 K. (* = compound **6**)

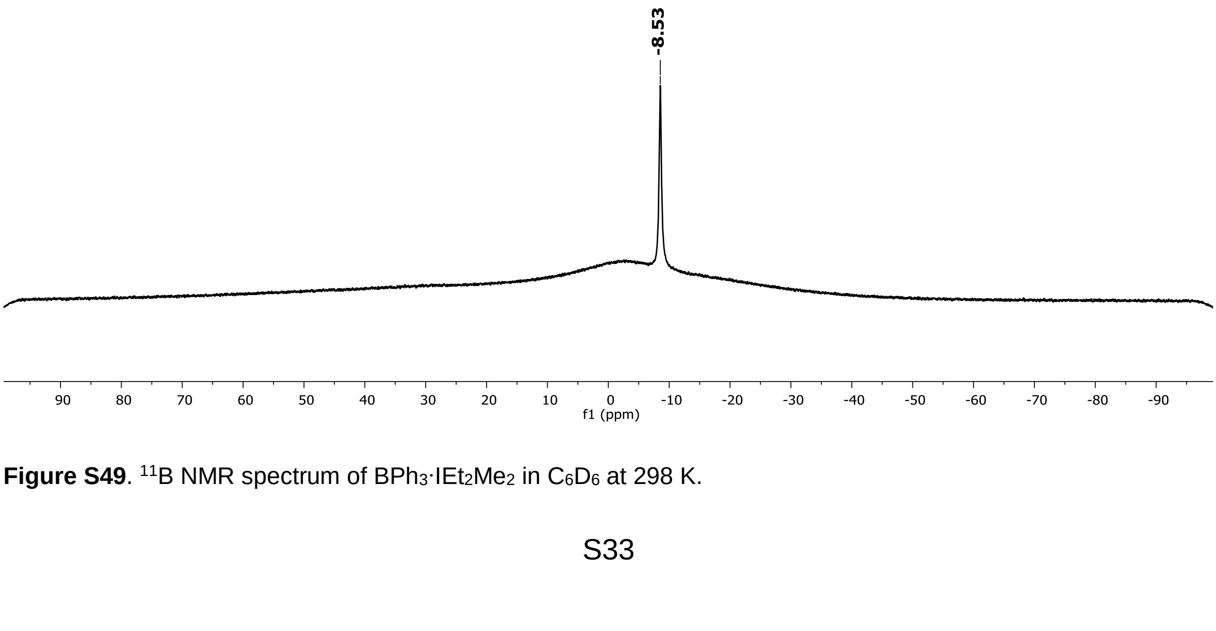

Figure S49. ^{11}B NMR spectrum of $BPh_3{\cdot}IEt_2Me_2$ in C_6D_6 at 298 K.

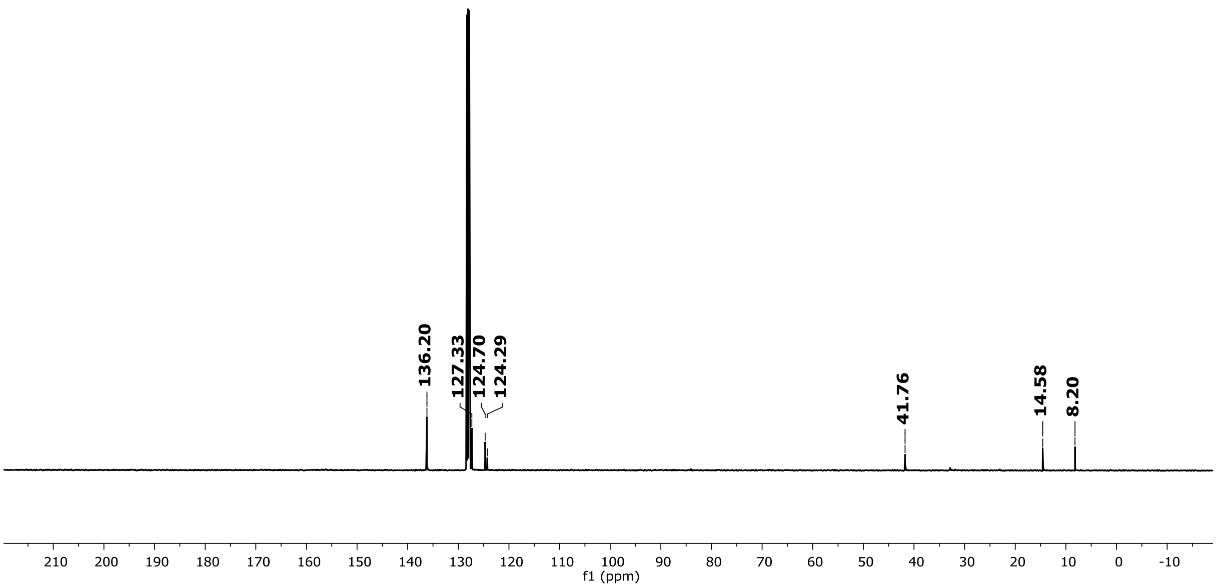

Figure S50. ^{13}C NMR spectrum of $BPh_3{\cdot}IEt_2Me_2$ in C_6D_6 at 298 K.

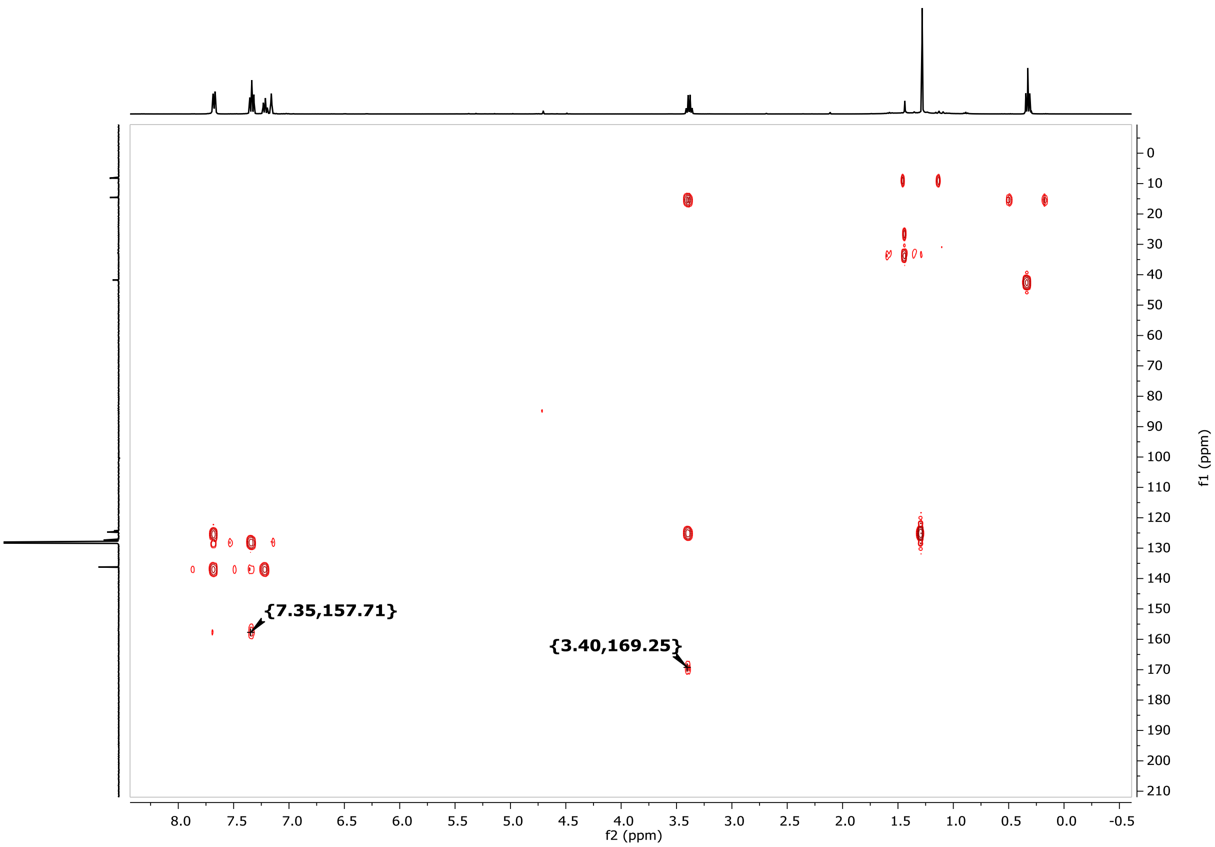

Figure S51. ^{1}H-^{13}C HMBC of $BPh_3{\cdot}IEt_2Me_2$ in C_6D_6 at 298 K.

2. Single Crystal X-ray structure determination

Single crystal diffraction data were recorded on a Bruker Photon CMOS system equipped with a Helios optic monochromator and a Mo IMS microsource λ (= 0.71073 Å) and an Atlas SuperNova system equipped with a mirror monochromator and a Cu micro-focus sealed X-ray tube (λ = 1.54178 Å). The data collection was performed, using the APEX III software package[4] and CrysAlisPro on single crystals coated with Fomblin ® Y as perfluorinated ether. The single crystal was picked on a micro sampler, transferred to the diffractometer and measured frozen under a stream of cold nitrogen. A matrix scan was used to determine the initial lattice parameters. Reflections were merged and corrected for Lorenz and polarization effects, scan speed, and background using SAINT.[5] Absorption corrections, including odd and even ordered spherical harmonics were performed using SADABS.[5] Space group assignments were based upon systematic absences, E statistics, and successful refinement of the structures. Structures were solved by direct methods with the aid of successive difference Fourier maps, and were refined against all data using the APEX III software in conjunction with SHELXL-2014[6] and SHELXLE.[7] H atoms were placed in calculated positions and refined using a riding model, with methylene and aromatic C–H distances of 0.99 and 0.95 Å, respectively, and Uiso(H) = 1.2·Ueq(C). Non-hydrogen atoms were refined with anisotropic displacement parameters. Full-matrix least-squares refinements were carried out by minimizing Σw(Fo2-Fc2)2 with the SHELXL weighting scheme.[8] Neutral atom scattering factors for all atoms and anomalous dispersion corrections for the non-hydrogen atoms were taken from International Tables for Crystallography.[9] The images of the crystal structures were generated by Mercury.[10] The CCDC numbers CCDC-1970332 (**2**), 1970333 (**3**), 1970334 (**3'**), 1970335 (**4a**), 1970336 (**6**) contain the supplementary crystallographic data for the structures **2** - **6**. These data can be obtained free of charge from the Cambridge Crystallographic Data Centre via https://www.ccdc.cam.ac.uk/structures/.

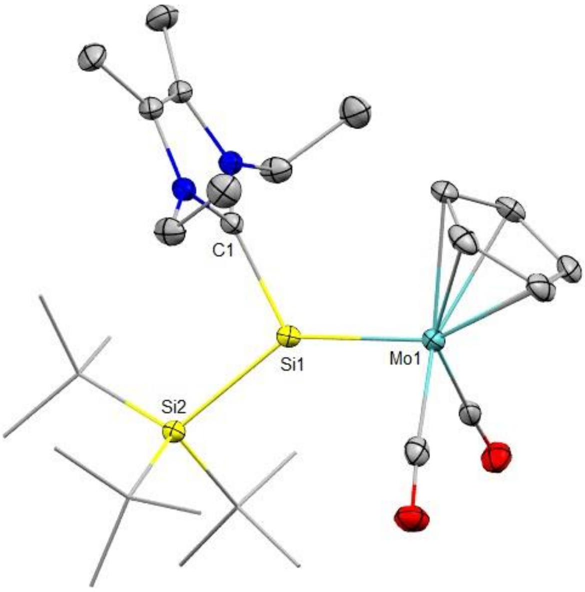

Figure S52. Ellipsoid plot (50% level) of the molecular structure of compound **2 (**one out of two independent molecules in the asymmetric unit is shown). Hydrogen atoms are omitted for clarity and *tert*-butyl groups are depicted in wireframe for simplicity. Selected bond lengths (Å) and angles (˚): Si1−Mo1 2.3499(8), Si1−Si2 2.4418(9), Si1−C1 1.949(2), C1−Si1−Si2 102.64(6), Mo1−Si1−C1 116.33(6), Mo−Si1−Si2 141.03(3)

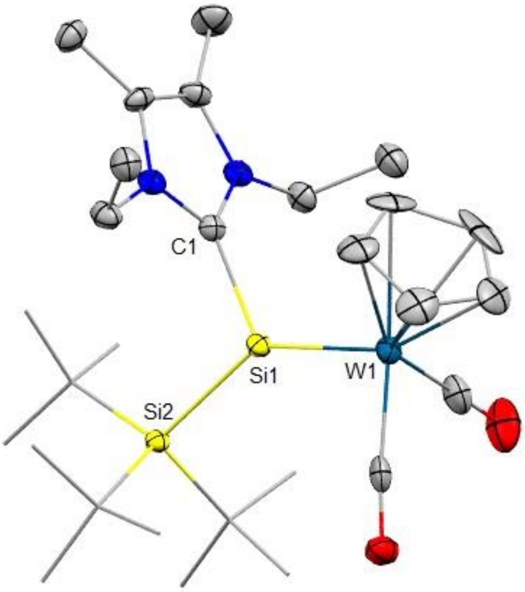

Figure S53. Ellipsoid plot (50% level) of the molecular structure of compound **3** (one out of three independent molecules in the asymmetric unit is shown). Hydrogen atoms are omitted for clarity and *tert*-butyl groups are depicted in wireframe for simplicity. Selected bond lengths (Å) and angles (˚): Si1−W1 2.346(2), Si1−Si2 2.428(3), Si1−C1 1.935(7), C1−Si1−Si2 105.1(2), W1−Si1−C1 113.5(2); W1−Si1−Si2 141.38(10).

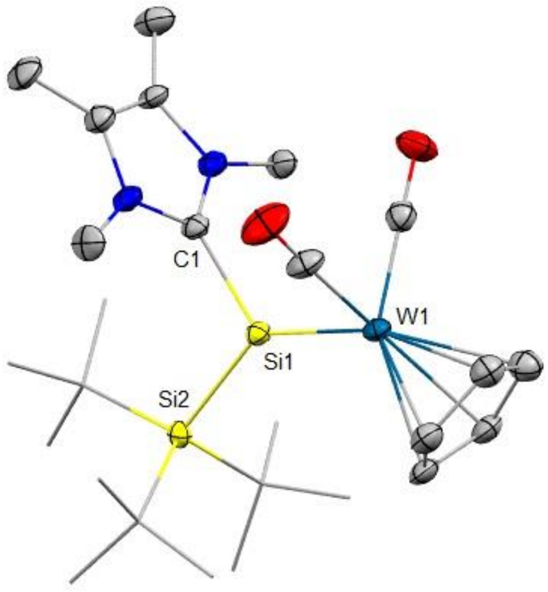

Figure S54. Ellipsoid plot (50% level) of the molecular structure of compound **3`**. Hydrogen atoms are omitted for clarity and *tert*-butyl groups are depicted in wireframe for simplicity. Selected bond lengths (Å) and angles (˚): Si1−W1 2.3534(12), Si1−Si2 2.4402(16), Si1−C1 1.941(5), C1−Si1−Si2 104.15(14), W1−Si1−C1 115.67(1); W−Si1−Si2 140.15(6).

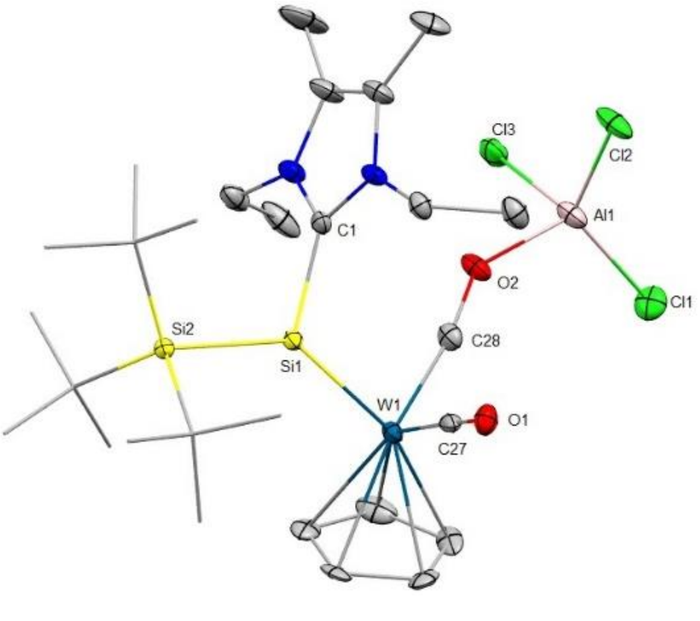

Figure S55. Ellipsoid plot (30% level) of the molecular structure of compound **4a**. Hydrogen atoms are omitted for clarity and *tert*-butyl groups are depicted in wireframe for simplicity. Selected bond lengths (Å) and angles (˚): Si1−W1 2.3630(18), Si1−Si2 2.437(2), Si1−C1 1.940(10), Al1−O2 1.777(8), W1−C27 1.975(7), W1−C28 1.840(7), C1−Si1−Si2 106.4(6), W1−Si1−C1 114.6(6), W−Si1−Si2 138.89(9).

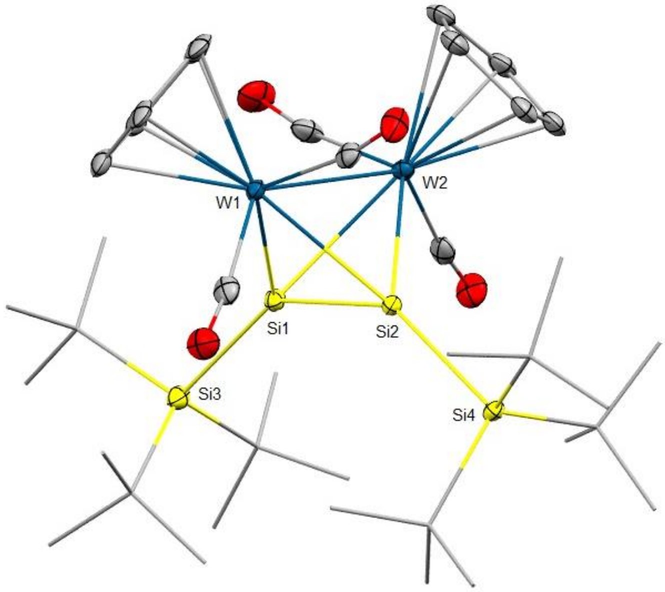

Figure S56. Ellipsoid plot (30% level) of the molecular structure of compound **6** (one out of three independent molecules in the asymmetric unit is shown). Hydrogen atoms are omitted for clarity and *tert*-butyl groups are depicted in wireframe for simplicity. Selected bond lengths [Å] and angles [˚]: Si1−W1 2.5507(15), Si1−W2 2.6913(15), Si2−W1 2.6790(14), Si2−W2 2.5593(14), W1−W2 3.0732(8), Si1−Si2 2.2221(19); W1−Si1−W2 71.73(4), W1−Si1−Si2 67.89(5), W2−Si1−Si2 61.92(5).

Table S1. Crystal data and structure refinement for compound **2**, **3**, **3'**, **4a** and **6**.

Compound #	2	3	3'	4a	6
Chemical formula	C28 H48 Mo N2 O2 Si2	C28 H48 N2 O2 Si2 W	C26 H44 N2 O2 Si2 W	C28 H48 Al Cl3 N2 O2 Si2 W	C38 H64 O4 Si4 W2
Formula weight	596.80 g/mol	684.70 g/mol	656.65 g/mol	818.03 g/mol	1064.93 g/mol
Temperature	100 K	100 K	150.00(10) K	100 K	100 K
Wavelength	0.71073 Å	0.71073 Å	1.54178 Å	0.71073 Å	0.71073 Å
Crystal size	0.142 x 0.109 x 0.104 mm	0.253 x 0.161 x 0.146 mm	0.187 x 0.165 x 0.144 mm	0.208 x 0.099 x 0.096 mm	0.265 x 0.231 x 0.189 mm
Crystal habit	clear green-blue fragment	clear blue fragment	clear dark green fragment	clear orange fragment	clear red fragment
Crystal system	triclinic	monoclinic	triclinic	monoclinic	triclinic
Space group	P -1	P 21/n	P -1	C 2/c	P -1
Unit cell dimensions	a = 11.022(3) Å; α = 87.215(12)°	a = 17.245(4) Å; α = 90°	a = 8.4517(2) Å; α = 88.492(2)°	a = 43.864(4) Å; α = 90°	a = 14.4964(17) Å; α = 86.770(4)°
	b = 16.013(5) Å; β = 74.756(11)°	b = 16.554(4) Å; β = 96.375(8)°	b = 11.0751(3) Å; β = 88.555(2)°	b = 10.3745(9) Å; β = 103.431(3)°	b = 17.691(2) Å; β = 87.223(4)°
	c = 17.938(6) Å; γ = 85.153(12)°	c = 32.197(8) Å; γ = 90°	c = 16.9012(5) Å; γ = 83.191(2)°	c = 17.2634(16)Å; γ = 90°	c = 24.954(3) Å; γ = 79.443(4)°
Volume	3042.5(16) $Å^3$	9135(4) $Å^3$	1569.92(7) $Å^3$	7641.2(12) $Å^3$	6276.5(13) $Å^3$
Z	4	12	2	8	6
Density (calculated)	1.303 g/cm^3	1.494 g/cm^3	1.389 g/cm^3	1.422 g/cm^3	1.691 g/cm^3
Radiation source	IMS microsource	IMS microsource	SuperNova (Cu) X-ray Source	IMS microsource	IMS microsource
Theta range for data collection	1.92 to 25.68°	2.05 to 25.37 °	2.62 to 73.81°	2.02 to 25.35°	1.91 to 25.35°
Index ranges	-13<=h<=13, -19<=k<=19, -21<=l<=21	-20<=h<=20, -19<=k<=19, -38<=l<=38	-9<=h<=10, -9<=k<=13, -20<=l<=20	-52<=h<=52, -12<=k<=12, -20<=l<=20	-17<=h<=17, -21<=k<=21, -30<=l<=30
Reflections collected	66492	16712	10942	64728	22936
Independent reflections	11544	13102	6143	7002	18819
Completeness	0.999	0.999	0.963	0.998	0.998
Absorption correction	Multi-Scan	Multi-Scan	Multi-Scan	Multi-Scan	Multi-Scan
Max. and min. transmission	0.7217 and 0.7467	0.6520 and 0.7452	0.54903 and 1.00000	0.6167 and 0.7452	0.6388 and 0.7416
Refinement method	Full-matrix least-squares on F^2	Full-matrix least-squares on F^2	Full-matrix least-squares on F^2	Full-matrix least-squares on F^2	Full-matrix least-squares on F^2
Function minimized	Σ w(F_o^2 - F_c^2)^2	Σ w(F_o^2 - F_c^2)^2	Σ w(F_o^2 - F_c^2)^2	Σ w(F_o^2 - F_c^2)^2	Σ w(F_o^2 - F_c^2)^2
Data / restraints / parameters	11544 / 0 / 657	16712 / 658 / 1151	6143 / 252 / 358	7002 / 566 / 506	22936 / 672 / 1443
Goodness-of-fit on F^2	1.036	1.147	1.064	1.074	1.140
Final R indices [I>2sigma(I)]	R1 = 0.0238, wR2 = 0.0533	R1 = 0.0430, wR2 = 0.0992	R1 = 0.0388, wR2 = 0.1152	R1 = 0.0457, wR2 = 0.1231	R1 = 0.0281, wR2 = 0.0585
R indices (all data)	R1 = 0.0288, wR2 = 0.0566	R1 = 0.0693, wR2 = 0.1247	R1 = 0.0404, wR2 = 0.1168	R1 = 0.0584, wR2 = 0.1313	R1 = 0.0453, wR2 = 0.0687
Largest diff. peak and hole	0.338 and -0.391 e$Å^{-3}$	6.273 and -3.373 e$Å^{-3}$	1.698 and -2.626 e$Å^{-3}$	3.499 and -2.028 e$Å^{-3}$	1.311 and -1.409 e$Å^{-3}$

3. Computational Data

General

All calculations were performed with ORCA v. 4.0.1.[11] The geometric parameters were optimized using the PBE0 functional,[12] with dispersion correction D3(BJ)[13] and the def2-SVP basis set.[14] For Si and W, the def2-TZVP basis set and the def2-ECP for W were used.[15] Tighter than default scf ("*tightscf*") and optimization criteria ("*tightopt*") were chosen in conjunction with finer than default grid values ("*grid6*"; "*nofinalgrid*"; *"gridx6"*). The RIJCOSX approximation with the related auxiliary basis sets (def2/J) was used.[16] The optimized geometric parameters were verified as true minima by the absence of negative eigenvalues in the harmonic vibrational frequency analysis (one for the transition states, respectively); calculated frequencies were scaled by 0.95 according to the *Database of Frequency Scale Factors for Electronic Model Chemistries* by D. Truhlar.[17] For the analysis of the electronic structure and final energies, single point calculations with either the def2/TZVPP basis set or scalar relativistic calculations with the Zeroth Order Regular Approximation (ZORA)[18] and the all-electron ZORA-def2-TZVPP basis set ("SARC-ZORA-TZVPP" for W; "old-ZORA-TZVPP" for Mo) were subsequently performed with even tighter grid settings (*"grid7"*, *"nofinalgrid"*). The latter method was also used to calculate the NMR shifts[19] and the TD-DFT transitions. For the TD-DFT calculations, 50 roots were calculated WITHOUT application of the Tamm-Dancoff approximation and with inclusion of solvation effects through the SMD model (solvent: TOLUENE).[20] The reported energies for the mechanism relate to the PBE0-D3BJ(SMD=TOLUENE)/ZORA-def2-TZVPP//PBE0-D3BJ/def2-SVP level of theory. Localized orbitals were generated using Knizia's Intrinsic Bond Orbitals at the PBE0-D3BJ/def2-TZVPP//PBE0-D3BJ/def2-SVP level of theory.[21] Single point calculations with the hybrid meta-GGA functionals M06[22] (ZORA-def2-TZVPP) with D3ZERO[23] dispersion correction and TPSSh[24] (ZORA-def2-TZVPP) with D3BJ dispersion correction gave consistent results (vide infra).

For compound **3**, three conformers (NHC-Si-W-Cp torsion) were calculated. The most stable isomer **3** is not the one obtained in the solid-state $\mathbf{3}^{\mathbf{XRAY}}$ ($\Delta\Delta G$ = +2.3 kcal mol^{-1} *vs.* the most stable conformer), but corresponds to the conformation found in **3'**. The energy of the third investigated isomer $\mathbf{3}^{\mathbf{rot}}$ is even higher ($\Delta\Delta G$ = +3.0 kcal mol^{-1} *vs.* the most stable conformer). The most stable conformer **3** was used for the calculation of the dimerization mechanism.

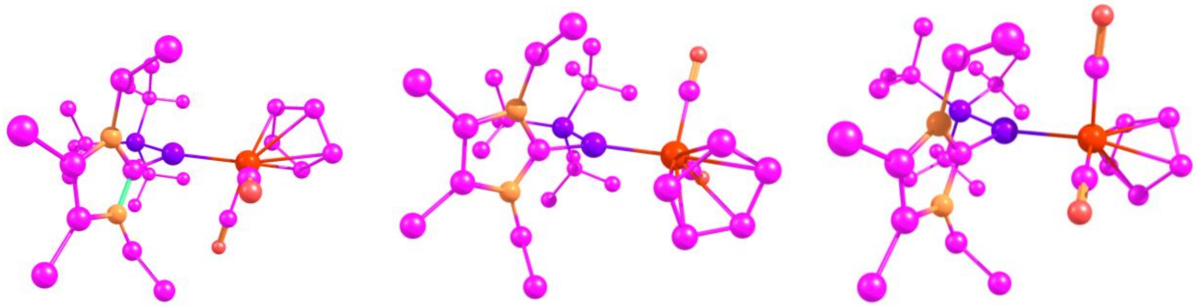

Figure S57. The lowest-energy isomer **3** (left), the solid-state structure conformer **3**$^{\mathbf{XRAY}}$ (middle) and the third investigated isomer **3**$^{\mathbf{rot}}$ (right).

The calculated carbonyl stretching frequencies of the most stable isomer fit best to the experimental values.

Table S2. Comparison of experimental and calculated IR stretching frequencies for the CO ligands. Values are given in cm^{-1}.

	**Compound 3**		**Compound 3^{XRAY}**		**Compound 3^{Rot}**		**Compound 6**	
	**1**	**2**	**1**	**2**	**1**	**2**	**1**	**2**
Exp.	1770	1849	1770	1849	1770	1849	1860	1914
Calc.	1837	1892	1878	1921	1838	1912	1904	1916

Table S3. Comparison of experimental and calculated ^{29}Si NMR shifts for tungsten complexes. Values are given in ppm vs. $SiMe_4$; values are averaged for the two Si atoms for compound **6**.

	**Compound 3**		**Compound 3^{XRAY}**		**Compound 3^{Rot}**		**Compound 6**	
	**Si1 (Si=W)**	**Si2 (*t*Bu$_3$Si)**	**Si1 (Si=W)**	**Si2 (*t*Bu$_3$Si)**	**Si1 (Si=W)**	**Si2 (*t*Bu$_3$Si)**	**Si1 (Si−W)**	**Si2 (*t*Bu$_3$Si)**
Exp.	+230	+12	+230	+12	+230	+12	-63	+44
Calc.	+267	+10	+229	+3	+255	+1	-48	+22

Table S4. Comparison of experimental and calculated ^{29}Si NMR shifts for molybdenum complexes. Values are given in ppm vs. $SiMe_4$; values are averaged for the two Si atoms for compound **5**.

	**Compound 2**		**Compound 2^{XRAY}**		**Compound 2^{Rot}**		**Compound 5**	
	**Si1 (Si=Mo)**	**Si2 (*t*Bu$_3$Si)**	**Si1 (Si=Mo)**	**Si2 (*t*Bu$_3$Si)**	**Si1 (Si=Mo)**	**Si2 (*t*Bu$_3$Si)**	**Si1 (Si−Mo)**	**Si2 (*t*Bu$_3$Mo)**
Exp.	+279	+6	+279	+6	+279	+6	+4	+48
Calc.	+265	-2	+292	-10	+309	+1	+5	+51

Table S5. Comparison of bond lengths (in Å) and angles (in º) for compound **3**$^{\mathbf{XRAY}}$.

**Compd. 3^{XRAY}**					
	**Si1−Si2**	**W1−Si1**	**Si1−C1^{NHC}**	**Si2−Si1−W1**	**Si2−Si1−C1^{NHC}**
X−Ray	2.428(3)	2.346(2)	1.949(2)	141.38(10)	105.1(2)
Calc.	2.412	2.349	1.952	140.5	105.0

Table S6. Comparison of bond lengths (in Å) and angles (in º) for compound **6**.

Compd. 6	X−Ray	Calc.
W1−W2	3.073	3.065
W1−Si1	2.551	2.564
W2−Si2	2.559	2.559
W2−Si1	2.691	2.726
W1−Si2	2.679	2.706
Si1−Si2	2.222	2.235
Si1−Si3	2.437	2.423
Si2−Si4	2.429	2.416
Si1−W1−Si2	50.2	50.1
Si1−W2−Si2	50.0	49.9
Si3−Si1−Si2−Si4	3.5	2.7

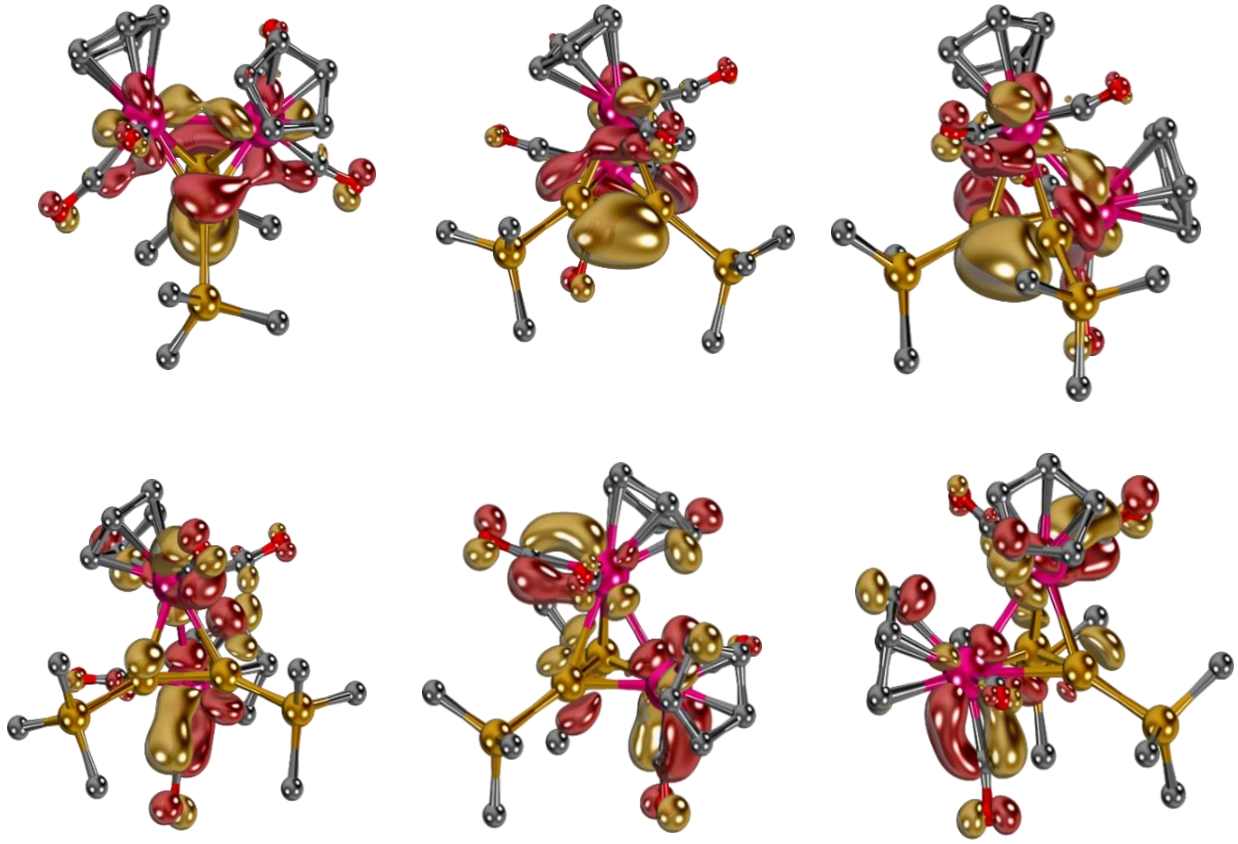

Figure S58. Canonical HOMO (top) and LUMO (bottom) molecular orbitals of **6** from different view angles.

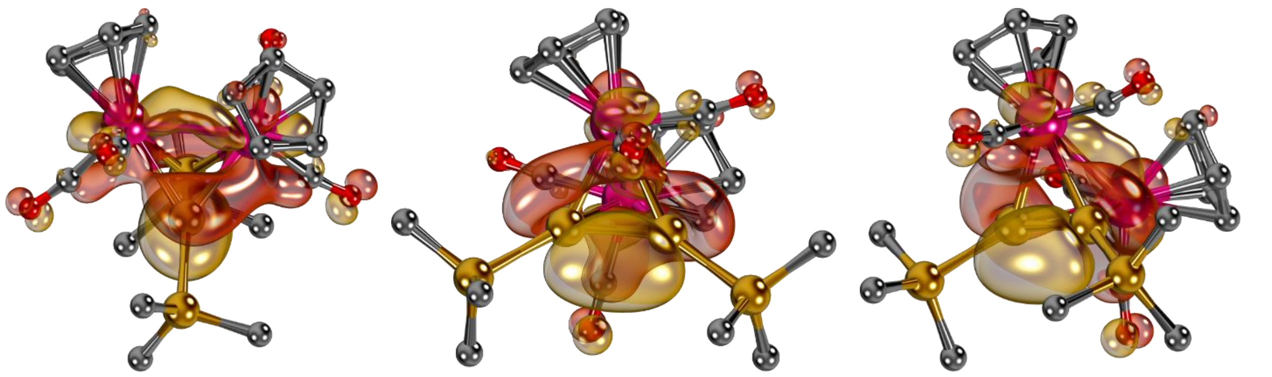

Figure S59. The canonical HOMO of molybdenum complex **5** (shown from three different perspectives) is delocalized, yet shows significant Si–Si π-character indicative of considerable multiple bond character.

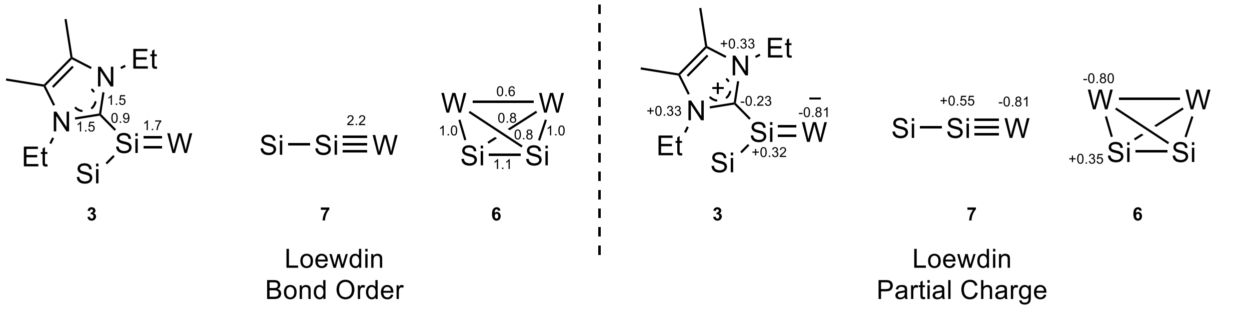

Figure S60. Löwdin bond orders and partial charges of compounds **3**, **6** and **7**.

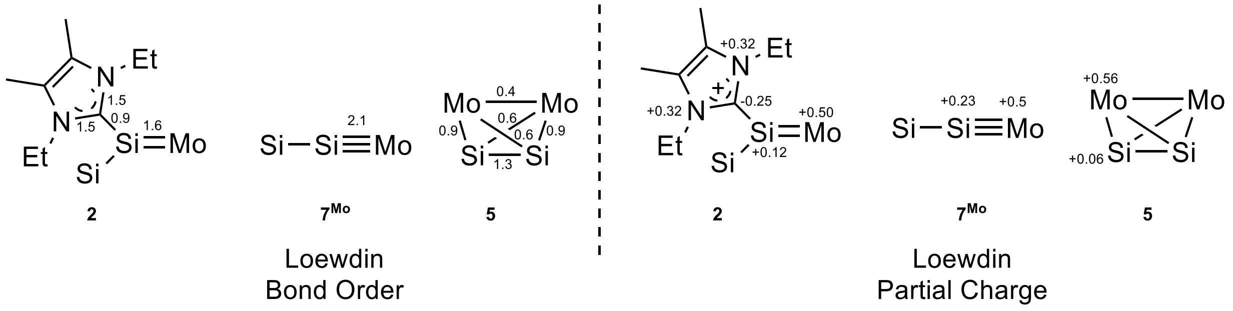

Figure S61. Löwdin bond orders and partial charges of compounds **2**, **5** and **7^{Mo}**.

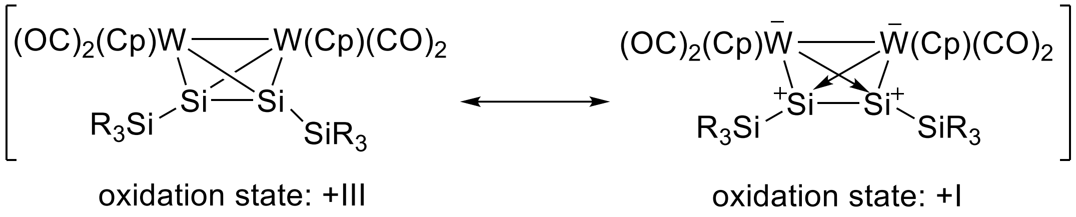

Figure S62. Important resonance structures of compound **6**.

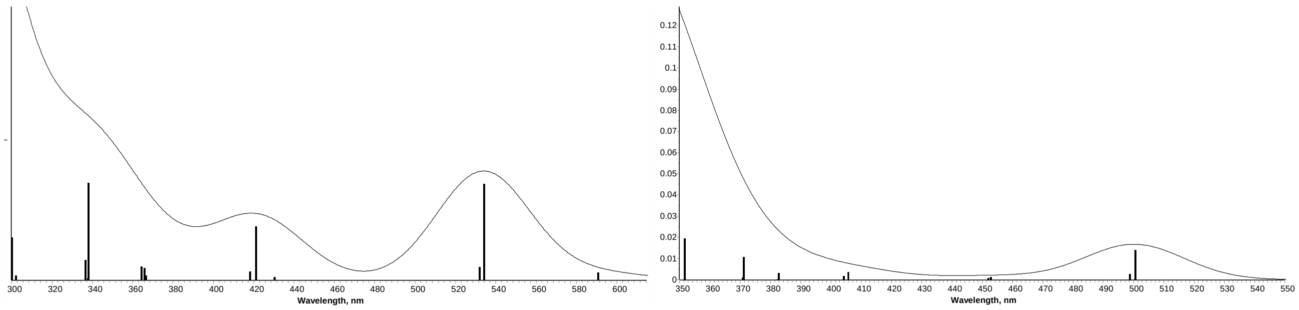

Figure S63. Calculated UV-Vis spectrum of compound **3** (left) and compound **6** (right).

Table S4. Excerpt of the TD-DFT calculations of the compounds **3** and **6** with approximate assignment of transitions.

**3**		**6**	
**Wavelength [nm]**	**MO Contributions**	**Wavelength[nm]**	**MO Contributions**
658	HOMO → LUMO	501	HOMO → LUMO
663	HOMO-1 → LUMO	499	HOMO-1 → LUMO
534	HOMO-2 → LUMO	452	HOMO → LUMO+1
411	HOMO-3 → LUMO	453	HOMO → LUMO+2
397	HOMO → LUMO+2	404	HOMO → LUMO
399	HOMO → LUMO+3		

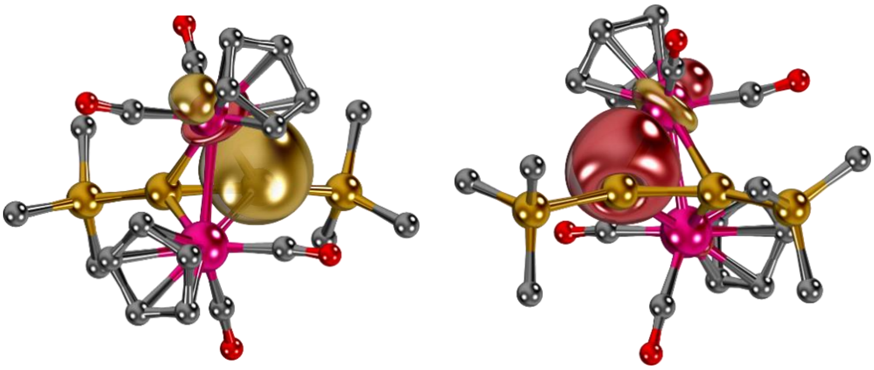

σ-bond Si1-W1

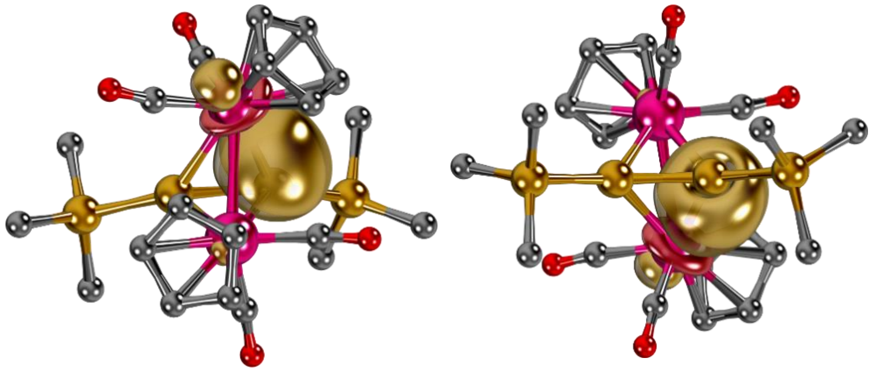

σ-bond Si2-W2

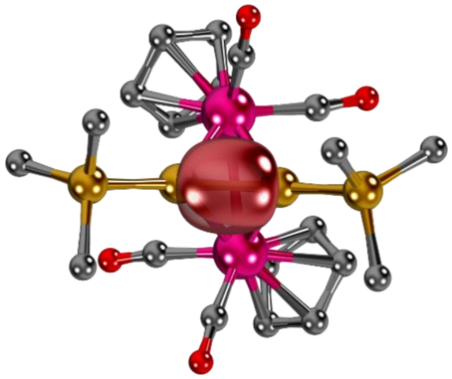

σ-bond Si1-Si2

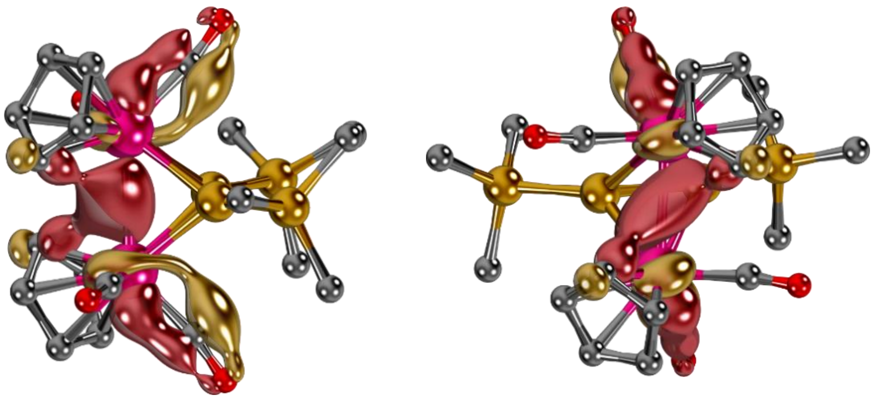

σ-bond W1-W2

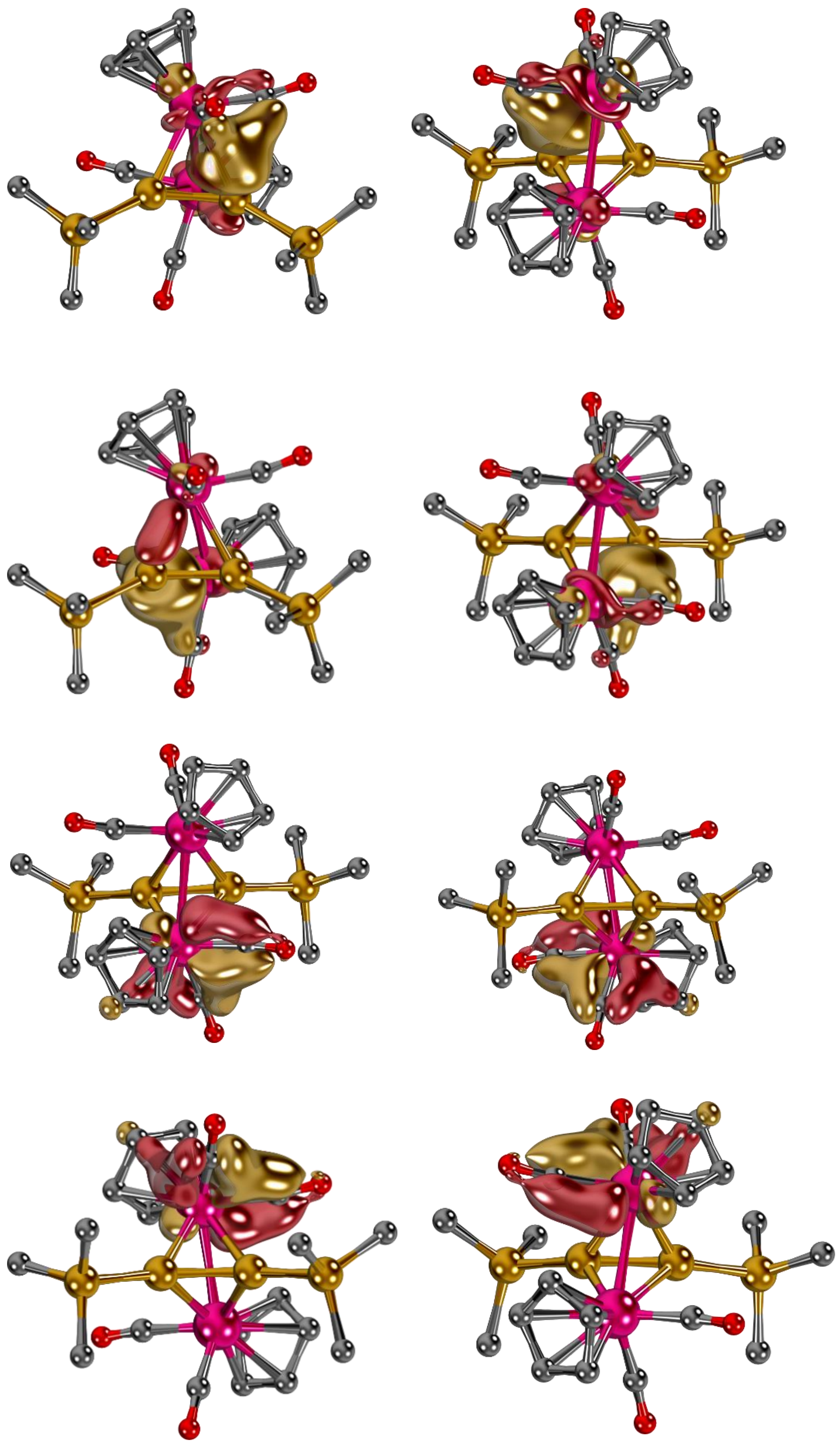

σ-bond Si1-W2

σ-bond Si2-W1

π-bond W1-CO

π-bond W2-CO

Figure S64. Intrinsic Bond Orbitals (IBOs) of compound **6**.

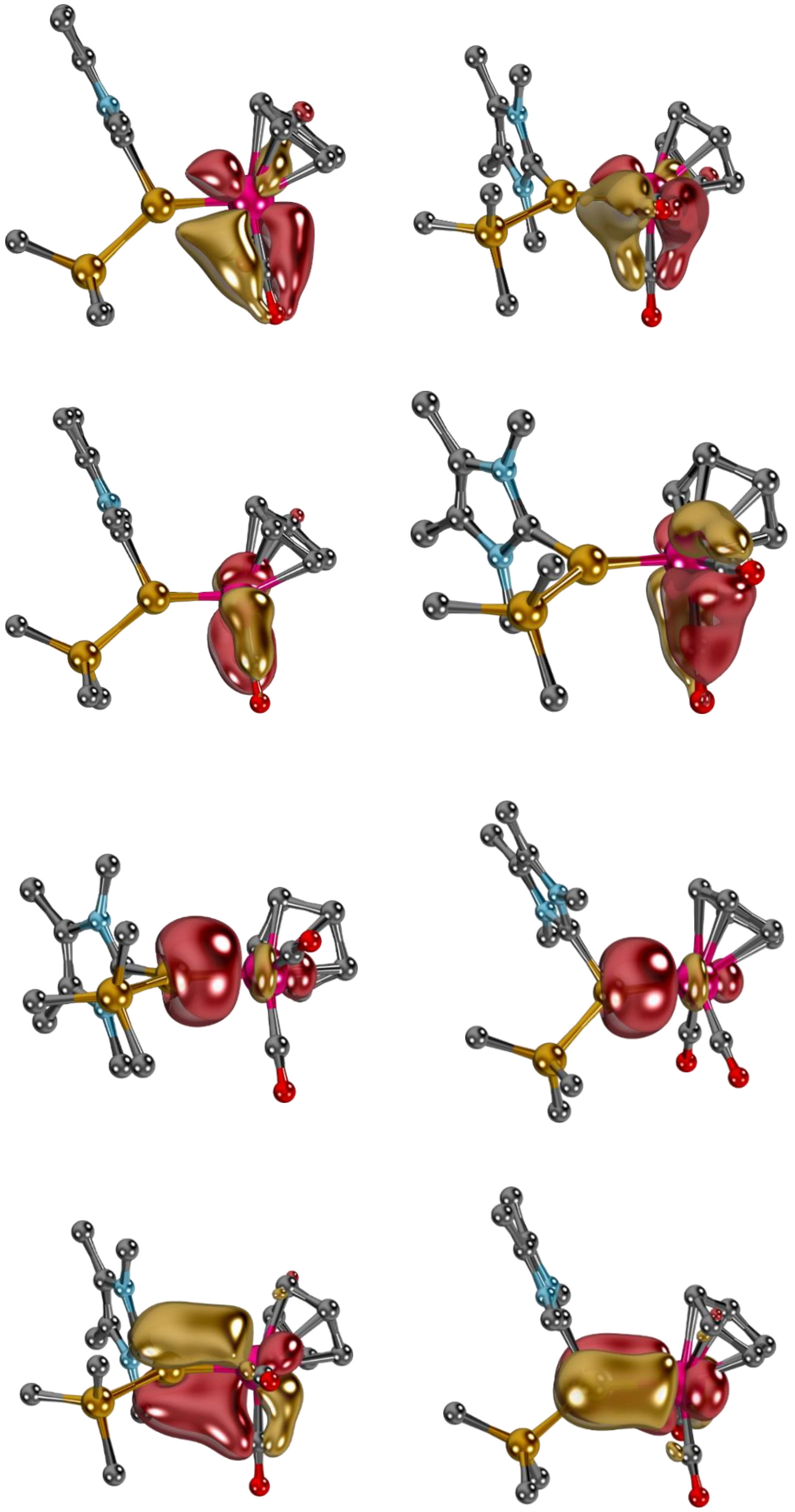

d_{yz} / π-bond CO

d_{xy} / π-bond CO

d_{z^2} / σ-bond Si

d_{z^2} / π-bond Si

Figure S65. Intrinsic Bond Orbitals (IBOs) of compound **3**$^{\mathbf{XRAY}}$.

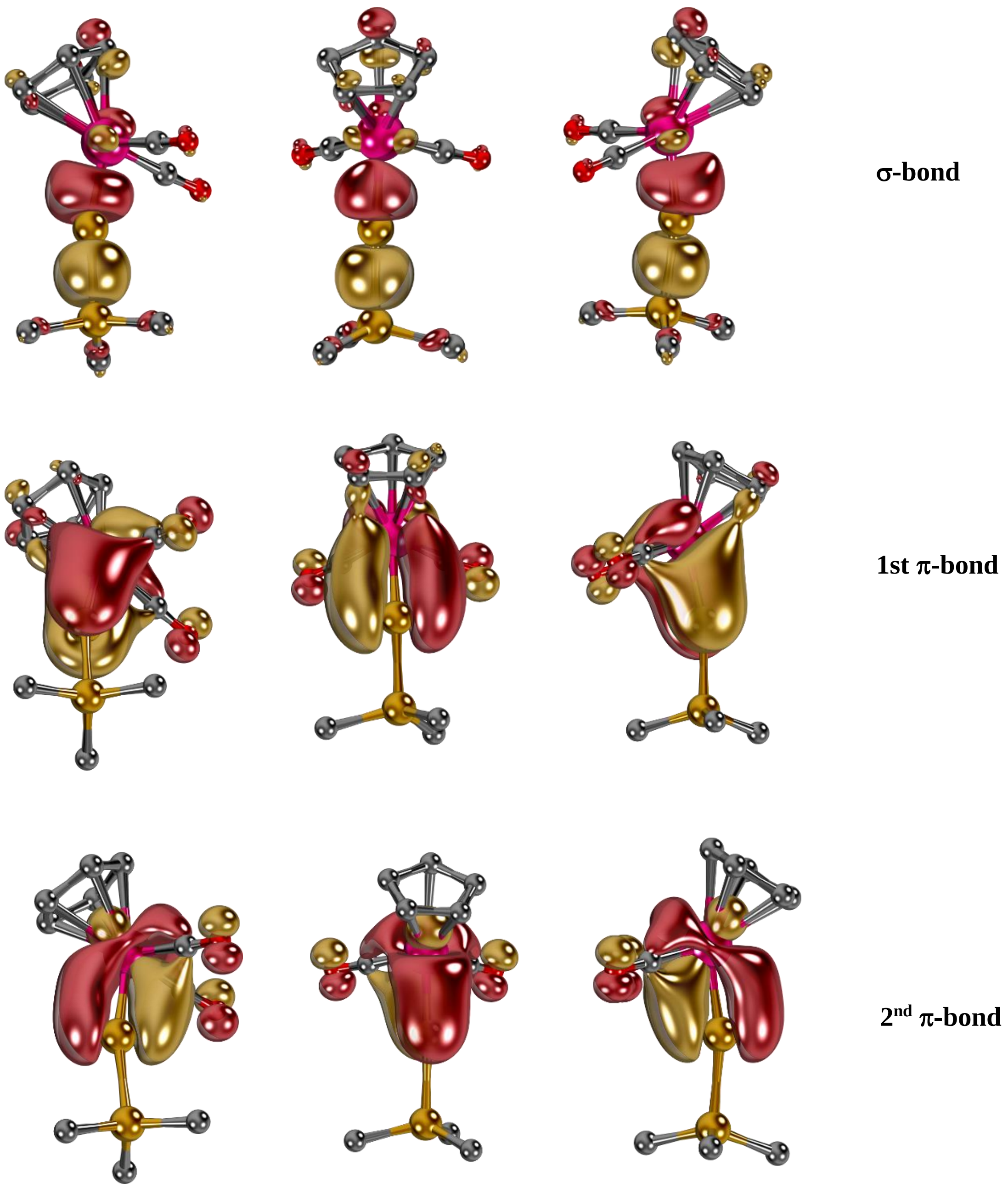

Figure S66. Intrinsic Bond Orbitals (IBOs) of calculated silylidyne **7.**

Multireference Character

Table S8. Vertical singlet/triplet gaps and Fractional occupation densities (FODs) of compounds **3**, **6**, **7** and transition state.

	**s/t gap [kcal mol^{-1}]**	**FOD**
**Compd. 3**	22	0.63
**Compd. 7**	25	0.44
**Transition State**	12.5	1.69
**Compd. 6**	27	0.53

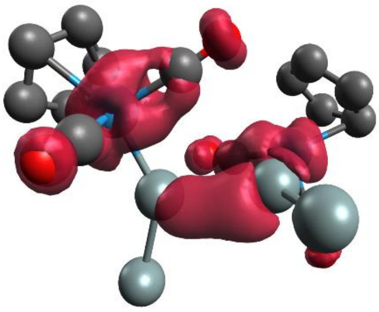

Figure S67. FOD plot of transition state (*t*Bu groups and H atoms omitted for clarity)

NEVPT2/CASSCF(12,12) calculations

State averaged (triplet, singlet) NEVPT2/CASSCF(12,12) calculations were performed on top of the DFT optimized transition state structure using the scalar relativistic ZORA method, the def2-TZVPP basis set and without using the frozencore approximation (*"nofrozencore*"). The calculations suggest a vertical singlet/triplet gap of 0.9 eV and indicate moderate multireference character. The active space relates to the metal valence d-orbitals, *viz.* the molecular orbitals associated with the π-/π*-interaction with the silylidyne / carbonyl ligands (antibonding and bonding interaction).

```
0.79876 [     0]: 222222000000
0.03449 [     7]: 222220200000
0.01441 [   111]: 222202020000
0.00985 [    35]: 222211110000
0.00834 [   781]: 222022002000
0.00817 [  1563]: 221221100100
0.00771 [ 15133]: 202222000020
0.00726 [ 49741]: 022222000002
0.00386 [   772]: 222022101000
0.00379 [  2070]: 221122001100
0.00378 [  7282]: 212122001010
0.00362 [   777]: 222022011000
0.00344 [   112]: 222202011000
0.00314 [  1560]: 221221200000
0.00274 [  2060]: 221122101000
0.00262 [  1632]: 221212110000
0.00251 [   275]: 222121100100
```

Figure S68. Weight of configuration state functions.

Energies

Table S9. Calculated Energies and imaginary frequencies.

	Imag [cm^{-1}]	PBE0/def2-SVP	PBE0/def2-SVP	PBE0/ZORA-def2-TZVPP// PBE0-def2-SVP	PBE0(SMD)/ ZORA-def2-TZVPP// PBE0/def2-SVP	PBE0/def2-TZVPP//PBE0/ def2-SVP	M06/ZORA-def2-TZVPP//PBE0-def2-SVP	TPSSh/ZORA-def2-TZVPP// PBE0/def2-SVP
		E in [H]	*G* in [H]	*E* in [H]	*E* in [H]	*E* in [H]	*E* in [H]	*E* in [H]
**2^{XRAY}**	-	-2000.24248	-1999.59267	-6024.41583	-6024.44417	-2001.91944	-6025.45827	-6026.65264
**2**	-	-2000.24781	-1999.59851	-6024.42144	-6024.44748	-2001.92503	-6025.46426	-6026.65807
**2^{rot}**	-	-2000.24426	-1999.59508	-6024.41727	-6024.44238	-2001.9209	-6025.4602	-6026.65445
**3^{XRAY}**	-	-1999.13023	-1998.48144	-18567.2136	-18567.2423	-2000.80334	-18568.4797	-18569.0821
**3**	-	-1999.13547	-1998.48713	-18567.2189	-18567.245	-2000.80866	-18568.4855	-18569.0874
**3^{rot}**	-	-1999.13221	-1998.48407	-18567.2154	-18567.2407	-2000.80497	-18568.482	-18569.0843
**Dimerization_ W_Silylidyne TS**	-52	-3075.69875	-3074.83416	-36212.383	-36210.2539	-3078.06816	-36212.383	-36212.7819
**5**	-	-3078.03052	-3077.15966	-11124.7358	-11124.76226	-3080.40433	-11126.43	-11128.0232
**6**	-	-3075.81128	-3074.94228	-36210.3398	-36210.3663	-3078.17885	-36212.4837	-36212.8895
**6^{isomer}**	-	-3075.79762	-3074.92756	-36210.326	-36210.3528	-3078.16608	-36212.4696	-36212.8754
**$6^{triangle}$**	-	-3075.76715	-3074.89987	-36210.2928	-36210.3211	-3078.13349	-36212.4354	-36212.8446
**$6^{quadrangle}$**	-	-3075.70572	-3074.84306	-36210.2796	-36210.3076	-3078.07667	-36212.3835	-36212.8315
**7**	-	-1537.84152	-1537.42475	-18105.1121	-18105.1297	-1539.0311	-18106.1871	-18106.3843
**7^{Mo}**	-	-1538.95499	-1538.53815	-5562.31354	-5562.33075	-1540.14716	-5563.16436	-5563.95395
**NHC**	-	-461.211612	-461.007568	-462.03349	-462.054493	-461.702762	-461.902537	-462.628445
**NHC_BPh_3**	-	-1179.77885	-1179.30746	-1181.81734	-1181.84028	-1181.01074	-1182.29374	-1183.35556
**NHC_BPh_3_ isomer**	-	-1179.77938	-1179.30768	-1181.81802	-1181.84713	-1181.01139	-1182.29501	-1183.35628
**BPh_3**	-	-718.48888	-718.249696	-719.714015	-719.733555	-719.237788	-720.001761	-720.658419
**Add NHC BPh_3 TS**	-51	-1179.71819	-1179.25024	-1182.25217	-1181.79097	-1180.95401	-1182.25217	-1183.30158
**3_minusCO**	-	-1885.91415	-1885.27032	-18453.7465	-18453.7701	-1887.45371	-18454.9503	-18455.4873
**CO**	-	-113.096482	-113.109772	-113.345053	-113.340665	-113.231974	-113.417016	-113.4731

Mechanism

The potential energy surface is very flat, complicating the search for transition states (cf. imaginary frequencies for transitions states). Accordingly, no transition states could be found for model systems with truncated ligands and barrierless dimerization was obtained instead. Furthermore, no transition state could be found for the dimerization of the silylidyne on the triplet hypersurface and instead also barrierless dimerization was predicted. Equally, no transition state could be found for the abstraction of the NHC from **3** by BPh_3. A scan of the NHC–Si bond distance indicates also here barrierless dissociation without interaction between the NHC and the silylidyne at a distance of 3.7 Å.

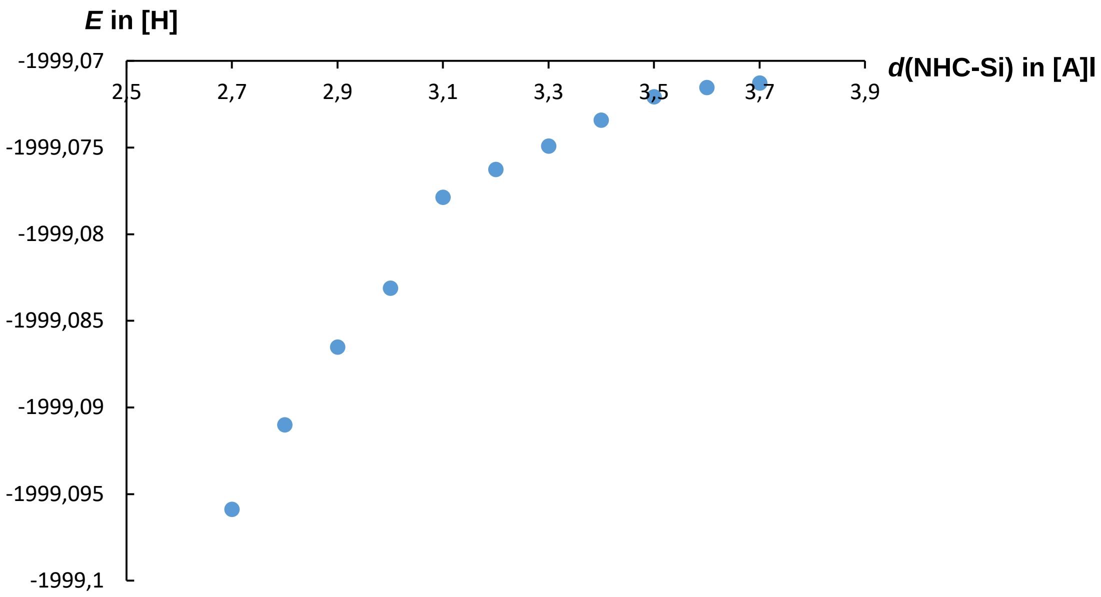

Figure S69. Scan of the NHC–Si distance in **3**.

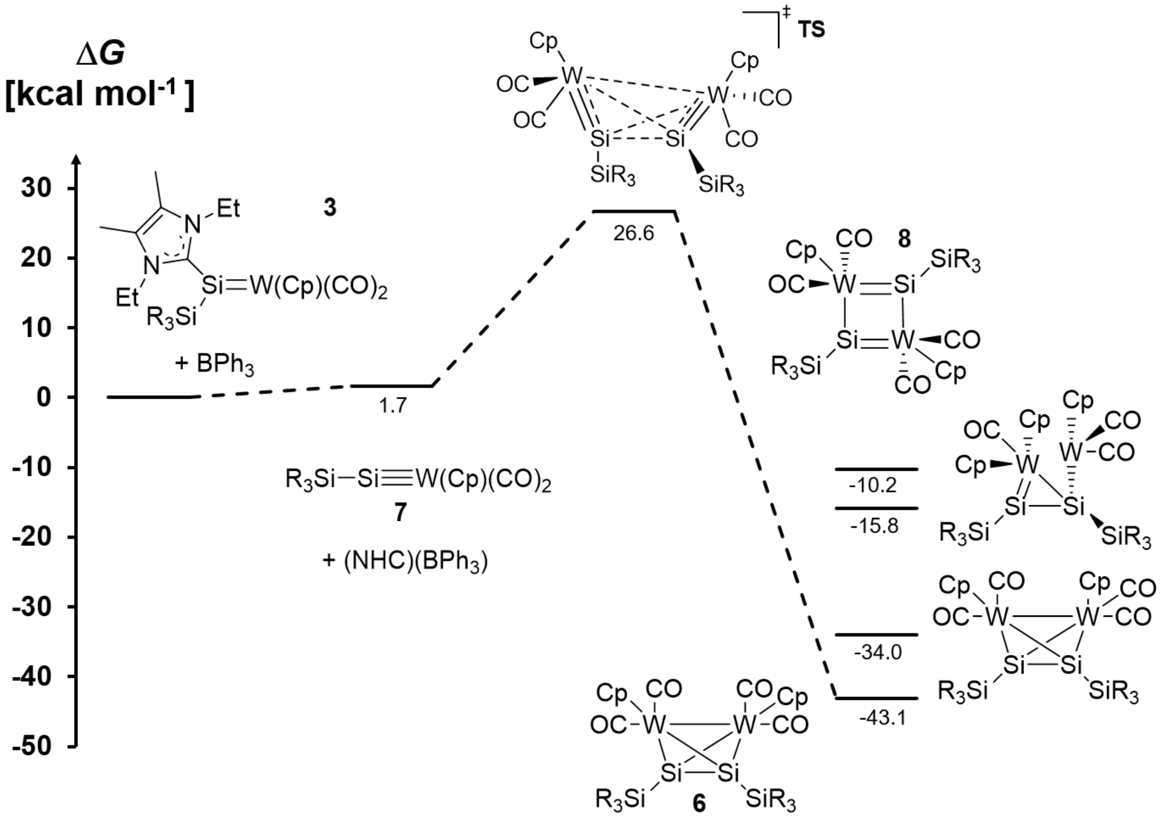

Figure S70. Calculated reaction profile for the formation of W_2Si_2 tetrahedral cluster (**6**).

The reaction of the free NHC with BPh_3 (Addition NHC BPh_3 TS) proceeds with a low barrier of 13.7 kcal mol^{-1} (imaginary frequency: 51 cm^{-1}).

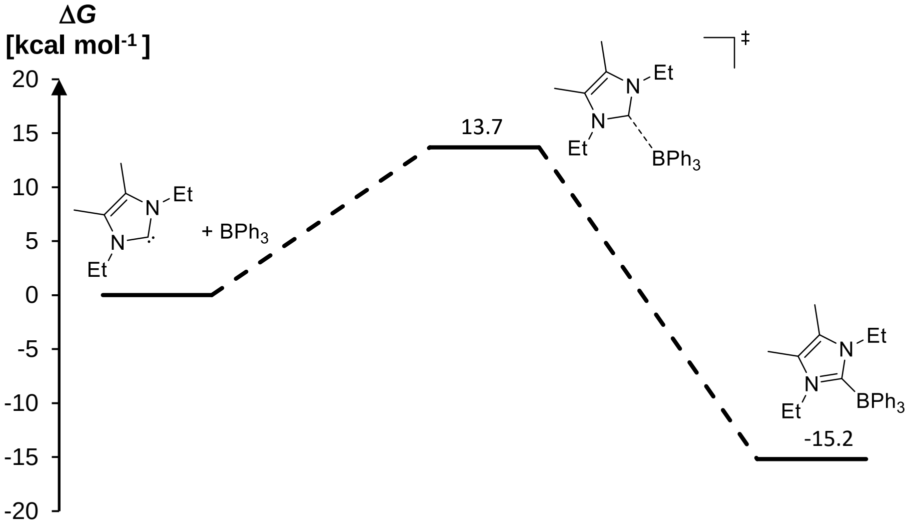

Figure S71. Calculated reaction profile for the reaction of the NHC with BPh_3.

XYZ Coordinates

Silylidene 3

W	5.80079	1.14558	8.94433
C	7.04750	1.33319	10.44622
O	7.73023	1.34996	11.39590
C	6.95458	2.28164	7.87700
O	7.64277	2.96573	7.21403
C	4.92286	-0.34194	7.33002
H	4.77398	-0.03554	6.29597
C	6.09525	-0.95130	7.87247
H	7.00959	-1.18668	7.33212
C	5.84027	-1.21476	9.24511
H	6.53865	-1.66979	9.94586
C	4.51127	-0.80585	9.55273
H	4.01927	-0.90067	10.51870
C	3.95276	-0.25381	8.37066
H	2.94725	0.15053	8.26423
Si	4.91555	3.09497	9.88313
Si	3.28507	3.68162	11.55707
C	5.83784	4.74317	9.41157
C	7.35150	6.39311	9.21159
C	6.40191	6.58265	8.24615
C	7.77023	4.62749	10.95877
H	7.13962	3.84309	11.39839
H	7.94785	5.38098	11.74265
C	9.06277	4.02417	10.44417
H	8.84854	3.29032	9.65654
H	9.57739	3.50377	11.26297
H	9.73909	4.78673	10.03302
C	8.55869	7.20057	9.51595
H	9.45780	6.79892	9.02287
H	8.75937	7.24214	10.59611
H	8.41737	8.23142	9.16426
C	6.33416	7.60876	7.17837
H	6.69979	7.21242	6.21711
H	6.96202	8.46934	7.44266
H	5.31028	7.97782	7.02133
C	4.34323	5.32709	7.52005
H	3.84324	6.29341	7.35474
H	3.63956	4.69453	8.08021
C	4.72002	4.65935	6.21206
H	3.84161	4.62472	5.55143
H	5.07235	3.63447	6.39821
H	5.51922	5.20380	5.69042
N	6.98740	5.24666	9.89990
N	5.48380	5.55580	8.39387
C	3.80987	2.67498	13.16705
C	5.03686	3.34469	13.79868
H	5.87545	3.40709	13.08693
H	5.38949	2.73753	14.65094
H	4.83064	4.35345	14.18451
C	4.23230	1.23693	12.83223
H	4.97513	1.19316	12.02362
H	3.38393	0.60613	12.53912
H	4.68788	0.77787	13.72843
C	2.68808	2.60193	14.21008
H	1.81600	2.04600	13.83541
H	2.33777	3.58732	14.54534
H	3.05015	2.06134	15.10347
C	1.58183	3.03804	10.81587
C	1.57891	1.50746	10.86764
H	0.73025	1.11610	10.27840
H	1.46656	1.12515	11.89185
H	2.50125	1.08469	10.44289
C	0.33314	3.54235	11.54846
H	0.18861	4.62773	11.44638
H	0.34683	3.30585	12.62317
H	-0.56164	3.05577	11.11837
C	1.46741	3.42444	9.33492
H	2.28929	2.98441	8.74652
H	1.46605	4.50990	9.16609
H	0.52200	3.02896	8.92070
C	3.18730	5.61004	11.93348
C	4.57089	6.25955	12.06690
H	5.06658	6.35333	11.09260
H	5.24745	5.71637	12.73800
H	4.45924	7.28237	12.47148
C	2.41504	5.87502	13.23310
H	2.26116	6.96246	13.35811
H	2.97017	5.52537	14.11526
H	1.42451	5.39906	13.25054
C	2.48014	6.33829	10.78580
H	2.99640	6.18055	9.82638
H	2.48097	7.42665	10.97889
H	1.43166	6.03440	10.66371

Silylidene 3^{rot}

W	5.29667	1.17904	8.69639
C	5.73132	2.38735	7.25758
O	6.04227	3.10727	6.38065
C	3.42414	1.20774	8.08258
O	2.31326	1.12552	7.74076
C	5.48044	-0.84096	10.00825
H	4.81360	-1.06099	10.83945
C	5.27211	-1.20676	8.64668
H	4.41049	-1.74943	8.25910
C	6.38524	-0.76852	7.88121
H	6.54215	-0.92909	6.81616
C	7.27292	-0.08581	8.76426
H	8.22796	0.35688	8.48521
C	6.71153	-0.13665	10.07714
H	7.16672	0.27241	10.97729
Si	4.78101	3.08651	9.96159
Si	4.23998	3.68525	12.24542
C	4.84944	4.72340	8.89889
C	5.69382	6.45279	7.72967
C	4.38369	6.30918	7.37004
C	7.26542	5.22035	9.24734
H	7.10483	4.61781	10.14829

H	7.66961	6.18773	9.58176
C	8.23383	4.50464	8.32693
H	7.89191	3.47899	8.13483
H	9.21990	4.46717	8.81313
H	8.34673	5.01042	7.35871
C	6.71203	7.42706	7.26518
H	7.49137	6.93978	6.65910
H	7.21261	7.93683	8.10295
H	6.23766	8.19474	6.64105
C	3.54900	7.14746	6.47582
H	3.20487	6.60414	5.58397
H	4.12667	8.01537	6.13352
H	2.65854	7.52533	7.00298
C	2.58647	4.61997	7.88480
H	1.81331	5.38800	8.04536
H	2.46666	3.86507	8.67683
C	2.46155	3.96594	6.52017
H	1.53293	3.38198	6.47086
H	3.30108	3.27953	6.35133
H	2.45244	4.70556	5.70803
N	5.95325	5.46665	8.66868
N	3.89525	5.22818	8.09003
C	5.90382	4.36839	13.04971
C	6.13245	5.81624	12.59482
H	6.06665	5.93916	11.50446
H	7.13905	6.15129	12.90494
H	5.40851	6.50855	13.04664
C	7.11353	3.53094	12.60135
H	7.11007	3.29139	11.52566
H	7.16475	2.56755	13.12379
H	8.04739	4.07633	12.82761
C	5.88078	4.35698	14.58172
H	5.81235	3.33747	14.98662
H	5.04853	4.93949	14.99850
H	6.81450	4.79897	14.97452
C	3.70491	1.99509	13.07356
C	4.93093	1.09888	13.27358
H	4.60207	0.10128	13.61825
H	5.61808	1.48894	14.03745
H	5.49069	0.95967	12.33700
C	3.03278	2.19400	14.43800
H	2.07542	2.72962	14.36675
H	3.67032	2.74033	15.14815
H	2.81183	1.20671	14.88338
C	2.74179	1.23269	12.15284
H	3.21279	0.98380	11.18870
H	1.81991	1.78752	11.93456
H	2.44340	0.28597	12.64033
C	2.78196	5.00350	12.38294
C	2.97142	6.14820	11.38212
H	2.85538	5.79651	10.34847
H	3.94872	6.64160	11.45847
H	2.20058	6.92027	11.55606
C	2.68125	5.60800	13.79020
H	1.81967	6.29866	13.83435
H	3.57038	6.19446	14.06194
H	2.52990	4.84835	14.56902
C	1.42725	4.36246	12.05534
H	1.40868	3.91884	11.04684
H	0.64018	5.13736	12.09302
H	1.13867	3.58067	12.77002

Silylidene 3^{XRAY}

W	5.73523	1.01184	8.94357
C	3.90332	0.70141	8.32508
O	2.82883	0.49779	7.92253
C	5.13895	-0.19950	10.35426
O	4.81438	-0.98910	11.14858
C	8.13267	1.10793	8.71130
H	8.77689	1.45374	9.51655
C	7.72726	-0.23638	8.51119
H	8.00399	-1.08115	9.13982
C	6.90906	-0.28856	7.35175
H	6.45823	-1.18267	6.92598
C	6.78335	1.04178	6.84745
H	6.24806	1.32886	5.94353
C	7.54321	1.90910	7.68973
H	7.69837	2.97735	7.54176
Si	5.01265	2.95164	10.05299
Si	3.33844	3.68152	11.62823
C	5.90403	4.58718	9.46800
C	7.33071	6.32047	9.26153
C	6.40427	6.42272	8.26087
C	7.73986	4.66120	11.12204
H	7.13392	3.83125	11.51538
H	7.76400	5.44499	11.89621
C	9.13219	4.16941	10.77030
H	9.07559	3.38726	10.00266
H	9.61602	3.74053	11.65992
H	9.77620	4.97445	10.38855
C	8.44706	7.23662	9.60428
H	9.39705	6.93719	9.13320
H	8.61072	7.28525	10.69015
H	8.21020	8.25354	9.26279
C	6.29362	7.43114	7.17914
H	6.64387	7.03263	6.21337
H	6.91018	8.30728	7.41771
H	5.25855	7.77710	7.04127
C	4.41009	5.06366	7.54052
H	3.86465	6.00581	7.38110
H	3.74458	4.39077	8.09947
C	4.80808	4.41904	6.22858
H	3.92752	4.34414	5.57485
H	5.19136	3.40500	6.41472
H	5.57595	4.99867	5.69642
N	7.01321	5.17137	9.97116
N	5.54277	5.34684	8.41079
C	3.84564	2.85530	13.34077
C	4.97617	3.64234	14.01432
H	5.85231	3.73969	13.35314
H	5.31427	3.09299	14.91132
H	4.68045	4.64903	14.34365

C	4.39885	1.44604	13.10959
H	5.27419	1.45457	12.44436
H	3.66884	0.75653	12.67194
H	4.72054	1.01701	14.07638
C	2.66652	2.74916	14.31428
H	1.86625	2.10747	13.91957
H	2.22476	3.72182	14.57213
H	3.01131	2.28373	15.25581
C	1.66032	2.95529	10.91849
C	1.68124	1.42944	11.06614
H	0.84461	0.99206	10.49458
H	1.56871	1.10866	12.11144
H	2.60411	0.98347	10.67081
C	0.39733	3.48771	11.60767
H	0.23742	4.56203	11.43111
H	0.39926	3.32147	12.69507
H	-0.48346	2.96038	11.19810
C	1.54681	3.26193	9.41873
H	2.38202	2.82529	8.85257
H	1.49827	4.33736	9.20021
H	0.62474	2.80409	9.01864
C	3.20657	5.63312	11.84142
C	4.57644	6.30964	11.97844
H	5.09050	6.36711	11.01043
H	5.24435	5.80846	12.68947
H	4.44218	7.34864	12.33250
C	2.38689	5.96709	13.09548
H	2.21824	7.05826	13.14967
H	2.91101	5.67574	14.01640
H	1.40139	5.48181	13.10550
C	2.52749	6.27863	10.62946
H	3.06480	6.05079	9.69680
H	2.52356	7.37817	10.74419
H	1.48253	5.96385	10.50471

6

W	0.95300	7.82770	22.76892
C	1.42549	6.54499	24.18628
O	1.64348	5.90094	25.12143
C	2.71772	8.60767	23.10396
O	3.64859	9.22986	23.41587
C	-1.38523	8.34948	22.79252
H	-2.16475	7.72713	22.36470
C	-0.72850	9.43042	22.14386
H	-0.92387	9.77052	21.13130
C	0.18061	10.01368	23.06372
H	0.81254	10.87940	22.87055
C	0.08155	9.30327	24.30096
H	0.61672	9.53590	25.21968
C	-0.88148	8.26468	24.12090
H	-1.20691	7.55574	24.88061
W	1.63645	7.82696	19.78073
C	1.62757	6.42061	18.39725
O	1.64264	5.74536	17.45812
C	-0.29735	7.94424	19.42648
O	-1.38071	8.19973	19.10201
C	3.64406	9.10574	19.76039
H	4.56318	8.82065	20.26204
C	3.26551	8.75976	18.43197
H	3.84576	8.14823	17.74273
C	2.01927	9.39515	18.14378
H	1.48546	9.36949	17.19558
C	1.62393	10.11321	19.31434
H	0.72650	10.72026	19.42031
C	2.63138	9.94565	20.29859
H	2.64996	10.40407	21.28210
Si	0.58905	5.78884	21.25788
Si	-0.94319	3.91714	21.12540
C	-0.61245	2.70713	22.62512
C	-0.34643	3.47924	23.92237
H	0.56111	4.09040	23.85090
H	-0.18506	2.76263	24.74753
H	-1.16956	4.14058	24.21774
C	0.61989	1.84155	22.36838
H	0.48730	1.14744	21.52752
H	0.82960	1.22939	23.26298
H	1.50842	2.45170	22.17389
C	-1.80385	1.76576	22.84126
H	-2.06642	1.19610	21.93769
H	-2.70449	2.29206	23.18540
H	-1.54376	1.03227	23.62455
C	-0.57873	3.08004	19.39879
C	0.93436	2.92014	19.19832
H	1.13302	2.55813	18.17391
H	1.37619	2.19399	19.89197
H	1.47594	3.86768	19.32233
C	-1.22871	1.69628	19.27113
H	-2.32142	1.72488	19.39204
H	-0.82806	0.97301	19.99511
H	-1.02186	1.28995	18.26475
C	-1.11737	3.96176	18.26586
H	-0.77658	3.55931	17.29618
H	-0.75801	4.99909	18.32814
H	-2.21614	3.98316	18.24433
C	-2.75544	4.64398	21.27016
C	-3.83812	3.65161	20.83035
H	-3.75709	3.39213	19.76479
H	-4.82906	4.11938	20.97366
H	-3.83227	2.71716	21.40607
C	-2.87683	5.89576	20.40209
H	-2.73704	5.69900	19.33264
H	-2.13454	6.64928	20.68529
H	-3.87682	6.35028	20.52497
C	-3.04002	5.07105	22.71706
H	-2.23804	5.70552	23.12438
H	-3.16645	4.21882	23.39771
H	-3.97897	5.65302	22.75141
Si	2.75905	6.27817	21.48054
Si	4.92560	5.25611	21.79793
C	6.07512	6.45581	22.82579
C	7.52940	5.96584	22.87954
H	7.99970	5.95962	21.88515

H	7.64030	4.96439	23.31378
H	8.11883	6.65784	23.50657
C	6.10771	7.86998	22.23818
H	6.66341	7.92145	21.29425
H	6.61050	8.54809	22.94876
H	5.10751	8.27940	22.07562
C	5.52690	6.54773	24.25608
H	4.46833	6.84468	24.28567
H	6.09146	7.30882	24.82164
H	5.62306	5.59790	24.79976
C	5.60522	4.94336	19.99218
C	6.09532	6.24543	19.35311
H	6.38898	6.05104	18.30574
H	6.97649	6.66094	19.86036
H	5.30750	7.01138	19.33957
C	6.78203	3.95953	20.01980
H	7.17315	3.82777	18.99507
H	6.49402	2.96450	20.38567
H	7.61490	4.31602	20.64221
C	4.50123	4.39055	19.08576
H	4.89779	4.24160	18.06557
H	3.66136	5.09498	19.00733
H	4.10144	3.42885	19.42467
C	4.63032	3.57033	22.74844
C	3.55919	3.76250	23.82276
H	2.60724	4.07299	23.37021
H	3.82326	4.51053	24.57961
H	3.37840	2.80821	24.34880
C	4.10849	2.50144	21.78488
H	3.23515	2.85675	21.22023
H	3.79203	1.61421	22.36018
H	4.86647	2.16557	21.06498
C	5.90452	3.03592	23.41302
H	6.73093	2.89355	22.70103
H	5.68912	2.05211	23.86698
H	6.25982	3.68755	24.22304

6^{quadrangle}

W	3.01173	4.75285	21.61299
C	1.46312	5.82802	20.81261
O	0.52145	6.31099	20.35709
C	4.96311	4.78166	22.16039
O	6.05613	4.63142	22.50076
C	3.01372	2.86442	22.95333
H	3.60299	2.80464	23.86631
C	3.45074	2.47529	21.64040
H	4.42925	2.07196	21.38604
C	2.36785	2.65070	20.74314
H	2.36651	2.41628	19.68221
C	1.27306	3.15899	21.49327
H	0.28749	3.39186	21.09212
C	1.66315	3.28709	22.85682
H	1.03108	3.62686	23.67170
W	4.08861	7.75134	21.10799
C	2.37009	8.43144	20.25263
O	1.47985	8.93165	19.72260
C	6.02187	7.40739	21.75465
O	7.09864	7.18203	22.07715
C	4.86422	9.78746	20.04726
H	4.31508	9.84697	19.10736
C	4.33698	10.09074	21.35012
H	3.32888	10.44835	21.56146
C	5.45093	10.29228	22.21328
H	5.38161	10.51515	23.27695
C	6.63354	10.18875	21.46317
H	7.64574	10.24280	21.86365
C	6.28428	9.85149	20.15003
H	6.97996	9.66305	19.33202
Si	3.07004	6.81500	23.06211
Si	2.54856	7.31703	25.35234
C	0.61829	7.12778	25.57042
C	3.14039	9.13452	25.75826
C	3.55957	5.98222	26.35707
Si	4.08105	5.85187	19.63371
Si	4.94709	5.31058	17.48219
C	6.85339	5.43200	17.91584
C	4.35851	6.64777	16.18733
C	4.39731	3.51008	16.97038
C	2.71132	10.10879	24.65350
H	2.98666	9.73792	23.65522
H	1.63248	10.30556	24.65395
H	3.21894	11.07833	24.80424
C	4.67088	9.17206	25.81382
H	5.00340	10.21160	25.98577
H	5.08582	8.56669	26.63008
H	5.12421	8.83027	24.87031
C	2.58991	9.63826	27.09822
H	3.01335	10.63499	27.31782
H	1.49616	9.75170	27.08427
H	2.84922	8.98136	27.94083
C	0.22710	7.02039	27.04961
H	0.52574	7.90362	27.63099
H	-0.86989	6.92118	27.13975
H	0.67152	6.13949	27.53472
C	-0.09457	8.33304	24.94671
H	0.19264	8.48754	23.89347
H	-1.18708	8.17147	24.96597
H	0.10275	9.26554	25.49254
C	0.10634	5.87861	24.84794
H	0.51755	4.95553	25.27559
H	-0.99301	5.82442	24.94333
H	0.35063	5.89299	23.77359
C	4.93698	5.80138	25.70538
H	5.52832	6.72415	25.66734
H	5.52355	5.05455	26.26941
H	4.85044	5.42111	24.67581
C	3.73587	6.36293	27.83119
H	4.34130	7.27008	27.96445
H	2.77411	6.52519	28.34028
H	4.25873	5.55018	28.36628
C	2.85914	4.62147	26.28948
H	3.50867	3.85109	26.74229

H	1.90756	4.60211	26.83908
H	2.66107	4.32490	25.25000
C	2.96145	6.33204	15.64286
H	2.64860	7.14481	14.96484
H	2.92870	5.40247	15.05793
H	2.20784	6.26720	16.44321
C	4.26292	8.03125	16.84077
H	5.22236	8.39097	17.23396
H	3.90548	8.76609	16.09735
H	3.54795	8.03316	17.67676
C	5.33815	6.69807	15.00770
H	5.44626	5.72269	14.50965
H	4.97600	7.41429	14.24918
H	6.34093	7.03012	15.31354
C	7.25351	6.90311	18.07156
H	8.28391	6.96241	18.46659
H	7.23170	7.45593	17.12194
H	6.60189	7.42129	18.79013
C	7.75024	4.76776	16.86889
H	7.55549	3.68914	16.77107
H	7.64110	5.22205	15.87316
H	8.80912	4.87681	17.16637
C	7.10575	4.78292	19.28263
H	6.52312	5.27491	20.07784
H	6.87856	3.71013	19.31408
H	8.16584	4.90993	19.56487
C	4.81198	3.13496	15.54271
H	4.49065	2.10005	15.32383
H	4.34129	3.78242	14.78924
H	5.90008	3.17986	15.39075
C	2.87215	3.38893	17.07860
H	2.34494	3.95101	16.30101
H	2.57638	2.32944	16.96586
H	2.50007	3.74909	18.05111
C	5.00509	2.49430	17.94293
H	4.78110	2.75184	18.99082
H	4.58396	1.49234	17.74274
H	6.09665	2.41388	17.84007

$6^{triangle}$

W	1.82922	7.80405	21.69639
C	1.54025	9.69186	22.21706
O	1.40016	10.78401	22.56757
C	0.42453	7.67082	23.03910
O	-0.26736	7.57241	23.96712
C	2.90923	6.14551	22.86201
H	2.35835	5.44785	23.49070
C	3.43532	7.41175	23.26634
H	3.36615	7.84981	24.26062
C	4.09810	7.98062	22.13958
H	4.59985	8.94548	22.09978
C	4.02499	7.04895	21.06747
H	4.45126	7.20261	20.07987
C	3.29765	5.91561	21.51001
H	3.07373	5.02297	20.93233
W	0.94301	8.84286	18.75438
C	-0.35638	9.14493	20.23758
O	-1.26019	9.54242	20.85288
C	2.63313	9.40994	19.61206
O	3.65632	9.94427	19.79342
C	0.58725	9.15711	16.47431
H	0.65683	8.35508	15.74366
C	-0.60745	9.57119	17.13250
H	-1.58878	9.11280	17.03002
C	-0.29472	10.69732	17.94188
H	-0.99442	11.23214	18.58238
C	1.08302	10.98563	17.79754
H	1.62339	11.79154	18.29098
C	1.63493	10.03780	16.88181
H	2.66626	10.02184	16.53807
Si	0.26950	6.57622	20.09908
Si	-1.81780	5.39156	20.06522
C	-1.26101	3.52575	19.83112
C	-2.88183	6.05224	18.55670
C	-2.74002	5.64066	21.76671
Si	1.73671	6.55805	18.36974
Si	3.24323	5.32004	16.86857
C	4.50794	6.68422	16.26255
C	2.17288	4.59457	15.39081
C	4.13941	3.89375	17.85118
C	-2.03735	6.21573	17.28913
H	-1.14403	6.83063	17.47110
H	-1.70844	5.25701	16.87027
H	-2.63916	6.71731	16.50914
C	-3.44055	7.43565	18.91229
H	-3.91934	7.87917	18.02060
H	-4.21187	7.38491	19.69248
H	-2.66133	8.13105	19.25575
C	-4.05630	5.12453	18.22218
H	-4.66603	5.58189	17.42204
H	-3.71972	4.14549	17.84909
H	-4.72261	4.94936	19.07775
C	-2.37494	2.51059	20.10230
H	-3.27182	2.69093	19.49200
H	-2.00970	1.49587	19.85970
H	-2.68556	2.49870	21.15670
C	-0.77983	3.33265	18.39459
H	-0.00769	4.07442	18.13741
H	-0.32889	2.32966	18.28696
H	-1.59079	3.40394	17.65614
C	-0.06815	3.20168	20.74098
H	-0.33921	3.17146	21.80318
H	0.34513	2.21270	20.47184
H	0.74444	3.93718	20.62248
C	-2.82255	7.12171	22.14594
H	-3.51893	7.68299	21.51300
H	-3.17919	7.21441	23.18668
H	-1.85515	7.62725	22.09233
C	-4.17087	5.09139	21.73909
H	-4.81806	5.67483	21.06873
H	-4.23228	4.03633	21.43607
H	-4.60748	5.17223	22.75080

C	-1.96018	4.91533	22.87208
H	-2.39503	5.17504	23.85263
H	-2.00751	3.82156	22.77243
H	-0.90179	5.21303	22.90348
C	1.57011	3.22695	15.72992
H	0.86494	2.94025	14.93119
H	2.32799	2.43484	15.78577
H	1.00896	3.22666	16.66903
C	1.00652	5.54341	15.09394
H	1.34157	6.53982	14.77276
H	0.38168	5.12983	14.28222
H	0.35246	5.66785	15.97114
C	3.00829	4.39917	14.11759
H	3.86816	3.73329	14.28321
H	2.37787	3.92452	13.34472
H	3.38401	5.34002	13.69362
C	3.80008	7.63331	15.29410
H	4.46228	8.48578	15.06035
H	3.53329	7.15866	14.34055
H	2.88378	8.03879	15.74301
C	5.73477	6.08006	15.56774
H	6.35389	5.49223	16.26052
H	5.47549	5.43649	14.71523
H	6.37205	6.89497	15.18041
C	4.99082	7.54421	17.43666
H	4.15430	8.03011	17.96030
H	5.58099	6.97941	18.16966
H	5.64162	8.34966	17.05382
C	4.86019	2.92816	16.89872
H	5.40814	2.17636	17.49428
H	4.17469	2.37817	16.24068
H	5.59969	3.43814	16.26484
C	3.13141	3.11250	18.70178
H	2.39874	2.56081	18.10035
H	3.66423	2.37699	19.32971
H	2.56100	3.77581	19.36843
C	5.19471	4.46869	18.79817
H	4.77671	5.20329	19.49587
H	5.62536	3.65301	19.40565
H	6.02525	4.94553	18.26002

6^{isomer}

W	2.43224	7.39341	21.84786
C	0.72339	8.04323	22.61734
O	-0.17202	8.49848	23.18264
C	1.91253	5.59321	22.46020
O	1.70850	4.56892	22.95902
C	3.46243	7.62666	23.87545
H	2.99029	7.27759	24.79227
C	3.35984	8.93486	23.30953
H	2.77264	9.76636	23.69520
C	4.19228	8.98110	22.15702
H	4.34374	9.84691	21.51852
C	4.79283	7.70984	21.98195
H	5.50468	7.45586	21.20460
C	4.33547	6.86540	23.03605
H	4.64103	5.83485	23.20760
W	1.57227	8.81040	19.15827
C	-0.26124	8.92708	19.80312
O	-1.34230	9.21819	20.12176
C	1.91392	9.97972	20.69259
O	2.01957	10.89609	21.41025
C	0.79648	9.44774	17.06777
H	-0.19797	9.16096	16.73129
C	1.12928	10.63322	17.79533
H	0.43237	11.40365	18.12053
C	2.54030	10.64764	17.98323
H	3.10358	11.42013	18.50484
C	3.07976	9.47908	17.40659
H	4.13327	9.21979	17.37032
C	1.99744	8.72574	16.84255
H	2.08029	7.78527	16.30310
Si	0.64293	6.50318	20.01129
Si	-1.45514	5.27469	19.81751
C	-0.86971	3.43103	19.52818
C	-2.40467	5.93919	18.23151
C	-2.51932	5.44992	21.44146
Si	2.79611	6.52810	19.42957
Si	4.45857	5.12977	18.36381
C	5.80587	6.33433	17.60098
C	3.58896	4.07940	16.96351
C	5.23285	3.95842	19.72895
C	-1.40720	6.28704	17.11799
H	-0.63390	6.99028	17.46540
H	-0.89264	5.40593	16.71430
H	-1.94510	6.76686	16.28022
C	-3.22438	7.19736	18.53720
H	-3.66835	7.56990	17.59643
H	-4.06046	6.98857	19.21937
H	-2.63164	8.00854	18.97051
C	-3.40557	4.89952	17.70278
H	-3.95035	5.33815	16.84773
H	-2.93597	3.97499	17.34348
H	-4.15725	4.62482	18.45643
C	-1.96369	2.39300	19.80296
H	-2.87867	2.56525	19.21756
H	-1.58059	1.39395	19.52670
H	-2.24396	2.34691	20.86460
C	-0.40565	3.24761	18.08194
H	0.33631	4.00238	17.79085
H	0.06821	2.25734	17.97282
H	-1.23106	3.28706	17.36075
C	0.32093	3.12993	20.44056
H	0.08229	3.23213	21.50655
H	0.66920	2.09430	20.27338
H	1.16893	3.79949	20.23100
C	-2.72789	6.91692	21.82649
H	-3.30289	7.48048	21.08422
H	-3.28588	6.96461	22.77895
H	-1.78488	7.44879	21.98075
C	-3.90448	4.81318	21.25140
H	-4.50202	5.34965	20.50093

H	-3.86809	3.75548	20.95880
H	-4.45956	4.86868	22.20462
C	-1.81649	4.77031	22.62272
H	-2.41932	4.92294	23.53530
H	-1.69441	3.68586	22.49199
H	-0.82307	5.20067	22.81474
C	2.88255	2.86446	17.57114
H	2.27667	2.36994	16.79175
H	3.59124	2.11687	17.94901
H	2.20750	3.13498	18.38992
C	2.53495	4.93486	16.25001
H	2.98715	5.75446	15.67477
H	1.96327	4.31277	15.53798
H	1.81636	5.36983	16.96306
C	4.57935	3.54074	15.92331
H	5.35615	2.90606	16.37460
H	4.02962	2.91406	15.19814
H	5.07889	4.33116	15.34817
C	5.28131	6.95712	16.30256
H	5.95376	7.77234	15.98263
H	5.24359	6.23402	15.47762
H	4.27668	7.38237	16.41339
C	7.12323	5.61881	17.26866
H	7.63220	5.23451	18.16377
H	6.98524	4.77990	16.57178
H	7.81420	6.33230	16.78462
C	6.11121	7.47576	18.57971
H	5.19746	7.98609	18.92620
H	6.66012	7.12433	19.46468
H	6.75264	8.22791	18.08634
C	6.03835	2.81482	19.09850
H	6.47985	2.19962	19.90291
H	5.42148	2.14340	18.48696
H	6.86706	3.17536	18.47248
C	4.12883	3.37182	20.61448
H	3.43418	2.72229	20.06972
H	4.58432	2.76548	21.41777
H	3.53670	4.16384	21.09381
C	6.17266	4.72833	20.65745
H	5.64181	5.53853	21.16738
H	6.55782	4.04519	21.43531
H	7.04358	5.15228	20.13889

7

W	5.76185	1.50307	8.62325
C	4.67350	2.72465	7.51904
O	4.06426	3.43429	6.83331
C	4.20481	0.29797	8.59241
O	3.30746	-0.43694	8.55388
C	7.90567	0.68374	9.28237
H	8.13274	0.46612	10.32478
C	7.34756	-0.22763	8.34890
H	7.08646	-1.26413	8.55659
C	7.22336	0.44320	7.09250
H	6.85422	0.00874	6.16541
C	7.69888	1.77736	7.26846
H	7.74949	2.54582	6.49859
C	8.12371	1.92486	8.61503
H	8.53874	2.82668	9.06223
Si	4.67571	2.43643	10.34503
Si	3.14132	3.40328	11.85138
C	3.81864	2.92884	13.61890
C	5.01446	3.80988	13.99550
H	5.82255	3.75712	13.24837
H	5.43639	3.45664	14.95324
H	4.73950	4.86628	14.12790
C	4.32060	1.47726	13.61882
H	5.11651	1.31133	12.87274
H	3.52843	0.74532	13.42066
H	4.75293	1.23948	14.60728
C	2.73783	3.07771	14.69495
H	1.87698	2.41811	14.51457
H	2.35979	4.10878	14.77297
H	3.15641	2.80218	15.67972
C	1.44089	2.56109	11.40011
C	1.43163	1.11441	11.90738
H	0.50300	0.61997	11.57379
H	1.45999	1.04260	13.00326
H	2.26941	0.52752	11.49866
C	0.24792	3.30911	12.00601
H	0.14520	4.32678	11.60037
H	0.30520	3.38780	13.10211
H	-0.68377	2.76764	11.76255
C	1.26462	2.49354	9.87581
H	2.10398	1.98344	9.37772
H	1.14763	3.47761	9.40732
H	0.36022	1.90583	9.63968
C	3.22694	5.30251	11.43083
C	4.68534	5.76427	11.29117
H	5.23460	5.16866	10.54229
H	5.24499	5.72212	12.23357
H	4.70616	6.81117	10.93836
C	2.52736	6.13193	12.51400
H	2.51297	7.19797	12.22440
H	3.04655	6.06672	13.48235
H	1.48275	5.82340	12.67200
C	2.56218	5.55896	10.07253
H	3.00463	4.94821	9.26869
H	2.71033	6.61680	9.79237
H	1.47887	5.37530	10.08346

7^{Mo}

Mo	5.81411	1.52968	8.68367
C	4.69836	2.72609	7.60947
O	4.05913	3.41569	6.93301
C	4.28071	0.33780	8.81014
O	3.39221	-0.40675	8.85187
C	7.95737	0.67483	9.18377
H	8.27805	0.50449	10.21031
C	7.27848	-0.25865	8.35953
H	6.99988	-1.27161	8.64612
C	7.05210	0.35252	7.08664

H	6.57750	-0.11337	6.22517
C	7.59777	1.66905	7.14038
H	7.60021	2.39432	6.32801
C	8.15263	1.86939	8.43058
H	8.63857	2.77597	8.78774
Si	4.80946	2.50780	10.40322
Si	3.16964	3.41973	11.85448
C	3.79568	2.93874	13.64015
C	4.96112	3.83737	14.06685
H	5.79456	3.81047	13.34670
H	5.35791	3.48051	15.03402
H	4.66023	4.88629	14.20281
C	4.32324	1.49670	13.64217
H	5.14634	1.35176	12.92144
H	3.55003	0.75411	13.41119
H	4.72727	1.25577	14.64184
C	2.67747	3.05567	14.68143
H	1.83863	2.37735	14.46985
H	2.27335	4.07733	14.75295
H	3.07001	2.78330	15.67783
C	1.49199	2.56100	11.34899
C	1.49784	1.10231	11.82187
H	0.58361	0.60173	11.45901
H	1.50570	1.00569	12.91627
H	2.35060	0.53681	11.41512
C	0.27238	3.27097	11.94889
H	0.15755	4.29628	11.56638
H	0.30362	3.32219	13.04737
H	-0.64276	2.71789	11.67089
C	1.34177	2.53059	9.82080
H	2.21411	2.08538	9.31964
H	1.17569	3.52156	9.38273
H	0.47433	1.90244	9.55324
C	3.22828	5.32718	11.46436
C	4.67875	5.82491	11.38977
H	5.27377	5.25004	10.65970
H	5.20094	5.78883	12.35356
H	4.68831	6.87477	11.04530
C	2.46335	6.13161	12.52155
H	2.43865	7.19977	12.24016
H	2.93830	6.06870	13.51260
H	1.41959	5.79977	12.62847
C	2.61450	5.58381	10.08201
H	3.09218	4.98168	9.29221
H	2.76167	6.64470	9.81294
H	1.53361	5.38883	10.05235

Dimerization_Siylidyne TS

W	1.40311	6.73536	22.73563
C	1.91511	4.89353	23.19323
O	2.17314	3.82108	23.55267
C	3.27500	7.28578	22.35007
O	4.32491	7.76027	22.26296
C	-0.62341	7.54164	23.66445
H	-1.60184	7.19925	23.34161
C	0.06171	8.68498	23.16227
H	-0.27039	9.32147	22.34472
C	1.27376	8.81336	23.88440
H	2.02072	9.59217	23.73730
C	1.34061	7.75887	24.84652
H	2.14075	7.59135	25.56573
C	0.16564	6.96423	24.69869
H	-0.10546	6.09788	25.30035
W	2.49451	8.80853	18.26328
C	1.57038	7.93983	16.75208
O	1.08249	7.49617	15.79660
C	0.89109	9.01135	19.34846
O	-0.01718	9.26253	20.03564
C	4.16457	9.89352	16.95788
H	4.60003	9.42062	16.07932
C	3.01043	10.73616	16.97568
H	2.39999	11.00511	16.11507
C	2.80120	11.17052	18.31068
H	1.99880	11.82287	18.65223
C	3.81427	10.59714	19.12899
H	3.94682	10.75205	20.19836
C	4.64928	9.80100	18.29015
H	5.53650	9.26274	18.61329
Si	0.58200	5.89609	20.74426
Si	-1.36565	4.87121	19.70311
C	-1.31525	2.97072	20.14593
C	-1.35232	5.21595	17.79840
C	-2.84096	5.77652	20.62245
Si	2.91272	6.70196	19.11845
Si	4.55117	4.87777	18.85462
C	6.21761	5.91676	18.81866
C	4.24299	4.06896	17.09526
C	4.50300	3.55235	20.27987
C	0.02284	4.85996	17.23012
H	0.82614	5.42831	17.72556
H	0.25248	3.79332	17.32469
H	0.06485	5.11873	16.15954
C	-1.60637	6.70248	17.52840
H	-1.46438	6.91202	16.45528
H	-2.62910	7.00800	17.78990
H	-0.90491	7.35131	18.07363
C	-2.41351	4.38959	17.06100
H	-2.38845	4.64150	15.98588
H	-2.23535	3.30700	17.14156
H	-3.43394	4.58943	17.41832
C	-2.72089	2.36712	20.03805
H	-3.13826	2.45255	19.02377
H	-2.67560	1.29187	20.28901
H	-3.43569	2.82462	20.73379
C	-0.39446	2.19558	19.19842
H	0.62464	2.60564	19.18068
H	-0.31844	1.14860	19.54159
H	-0.77055	2.17180	18.16571
C	-0.78899	2.75370	21.57100
H	-1.34530	3.31336	22.33157
H	-0.87232	1.68201	21.82576
H	0.27147	3.03178	21.66916

C	-2.58142	7.28685	20.67454
H	-2.66100	7.76343	19.69085
H	-3.32708	7.76737	21.33251
H	-1.58546	7.53655	21.06791
C	-4.18553	5.55487	19.91568
H	-4.19944	5.98363	18.90374
H	-4.46523	4.49484	19.83878
H	-4.98077	6.06243	20.49053
C	-2.96467	5.26530	22.06266
H	-3.70142	5.88174	22.60730
H	-3.31821	4.22678	22.11710
H	-2.01093	5.31930	22.60684
C	3.19191	2.95915	17.16831
H	2.96033	2.61038	16.14614
H	3.53881	2.08464	17.73582
H	2.25202	3.31007	17.61412
C	3.72521	5.10884	16.09480
H	4.38008	5.98192	15.98796
H	3.63206	4.63859	15.09945
H	2.73102	5.48474	16.37164
C	5.53278	3.44248	16.54238
H	5.96611	2.69064	17.21816
H	5.30422	2.92994	15.59091
H	6.30954	4.18620	16.32284
C	6.31475	6.72080	17.51560
H	7.18114	7.40466	17.57220
H	6.46830	6.08379	16.63463
H	5.41609	7.32957	17.33841
C	7.46223	5.02515	18.92931
H	7.55134	4.55899	19.92122
H	7.49767	4.22518	18.17761
H	8.36158	5.65059	18.78262
C	6.24629	6.90972	19.98228
H	5.38278	7.59048	19.96553
H	6.25066	6.41859	20.96329
H	7.16072	7.52793	19.92080
C	5.40694	2.34916	19.97854
H	5.31703	1.61383	20.79755
H	5.12873	1.82840	19.05100
H	6.46784	2.62863	19.90585
C	3.06987	3.04097	20.45851
H	2.73203	2.43909	19.60876
H	3.00679	2.40933	21.35972
H	2.35560	3.86904	20.59632
C	4.95514	4.16130	21.61060
H	4.36011	5.04065	21.89086
H	4.82512	3.41792	22.41524
H	6.01521	4.45156	21.59839

BPh$_3$

B	5.00819	-1.06686	-2.80148
C	5.05365	-0.81168	-1.25563
C	5.79497	-1.67835	-0.43042
C	4.42658	0.29136	-0.64517
C	5.95468	-1.42250	0.92934
C	4.54577	0.52880	0.72174
C	5.33105	-0.31629	1.50694
H	6.27264	-2.55665	-0.87361
H	3.83136	0.97413	-1.25721
H	6.56216	-2.09143	1.54395
H	4.03899	1.38334	1.17715
H	5.45919	-0.10837	2.57253
C	5.13408	0.13110	-3.79916
C	4.70102	0.02497	-5.13611
C	5.67024	1.36957	-3.39131
C	4.78258	1.10224	-6.01385
C	5.76122	2.44906	-4.26438
C	5.31072	2.31783	-5.57840
H	4.27222	-0.91722	-5.48599
H	6.03575	1.48108	-2.36796
H	4.42511	0.99663	-7.04127
H	6.18977	3.39353	-3.91961
H	5.37208	3.16523	-6.26606
C	4.82252	-2.53415	-3.31938
C	5.50541	-3.05132	-4.43687
C	3.89812	-3.37693	-2.67394
C	5.27049	-4.34657	-4.89286
C	3.62539	-4.65630	-3.15195
C	4.31357	-5.14349	-4.26282
H	6.23891	-2.42659	-4.95408
H	3.36995	-3.00762	-1.79062
H	5.82560	-4.73413	-5.75100
H	2.88129	-5.28120	-2.65194
H	4.10077	-6.14695	-4.64123

NHC_BPh$_3$

C	5.84621	4.71537	9.54301
C	7.31519	6.39897	9.19188
C	6.75407	6.08919	7.98710
C	6.97449	5.71436	11.55327
H	8.03502	5.96985	11.68726
H	6.79950	4.75206	12.04390
C	6.07893	6.78806	12.13787
H	6.24418	7.76466	11.65809
H	6.27388	6.89621	13.21476
H	5.02167	6.51385	12.00778
C	8.34933	7.40365	9.54800
H	9.23677	6.92862	9.99613
H	7.97389	8.14929	10.26602
H	8.68290	7.94192	8.65166
C	6.99510	6.68270	6.64619
H	7.56455	6.00817	5.98930
H	7.56757	7.61411	6.74125
H	6.05192	6.92294	6.13248
C	4.97947	4.50570	7.20106
H	4.46974	5.35463	6.71798
H	4.20498	3.93499	7.72199
C	5.68486	3.63498	6.17989
H	4.93677	3.19546	5.50478
H	6.21588	2.81158	6.67421
H	6.40016	4.20101	5.56623

N	6.74041	5.54652	10.12476
N	5.85403	5.06147	8.22991
B	4.93611	3.47425	10.16113
C	5.32494	2.19308	9.20304
C	4.37986	1.40350	8.53132
C	6.67482	1.85494	8.98600
C	4.74936	0.34558	7.69794
C	7.06045	0.79019	8.16972
C	6.09382	0.02791	7.51442
H	3.31743	1.63853	8.64053
H	7.45804	2.45981	9.45623
H	3.97764	-0.23365	7.18382
H	8.12060	0.55944	8.02958
H	6.38793	-0.80015	6.86437
C	3.35805	3.90409	10.09189
C	2.37941	2.99299	10.53517
C	2.89026	5.16842	9.70196
C	1.02482	3.31449	10.56463
C	1.53441	5.50746	9.72774
C	0.59222	4.57716	10.15495
H	2.69758	2.00959	10.89352
H	3.60360	5.93238	9.37538
H	0.29980	2.57719	10.91961
H	1.21340	6.50413	9.41153
H	-0.46927	4.83808	10.18063
C	5.27997	3.12063	11.73548
C	4.57365	3.74465	12.78218
C	6.25350	2.18473	12.12592
C	4.85168	3.49082	14.12486
C	6.53744	1.91351	13.46536
C	5.84369	2.57437	14.47646
H	3.77426	4.44947	12.53595
H	6.80525	1.63374	11.36264
H	4.27828	4.00417	14.90180
H	7.30023	1.17253	13.72024
H	6.06407	2.36004	15.52594

Addition NHC BPh_3 TS

C	6.78199	5.93522	7.43181
C	6.16955	6.31755	9.60464
C	5.11403	5.70952	8.98982
C	8.35769	7.22906	8.82937
H	9.08519	6.89737	8.08076
H	8.77195	6.98950	9.82173
C	8.10587	8.72028	8.69656
H	7.74562	8.95271	7.68307
H	9.03029	9.28707	8.88239
H	7.34436	9.06931	9.40969
C	6.36554	6.69121	11.02849
H	6.61741	7.75510	11.16400
H	7.17904	6.10270	11.48303
H	5.45507	6.48040	11.60552
C	3.77043	5.36793	9.52643
H	3.58437	5.92109	10.45696
H	3.65476	4.29393	9.74703
H	2.97487	5.64707	8.81806
C	4.69023	4.88727	6.65552
H	5.25537	5.00605	5.72194
H	3.76570	5.47914	6.54488
C	4.36067	3.42530	6.91574
H	3.92529	2.96621	6.01565
H	3.63614	3.29657	7.73160
H	5.26696	2.86544	7.18937
N	7.14972	6.45560	8.62992
N	5.52003	5.48941	7.67693
B	8.91246	4.21910	6.39204
C	8.52870	3.34825	7.65008
C	8.77536	3.80729	8.95853
C	7.89712	2.09762	7.53048
C	8.38123	3.09042	10.08258
C	7.48572	1.37415	8.64933
C	7.71874	1.87148	9.93039
H	9.26998	4.76910	9.09647
H	7.72157	1.67853	6.53696
H	8.58789	3.48861	11.08027
H	6.97772	0.41472	8.51986
H	7.38776	1.30812	10.80663
C	8.43646	3.82053	4.94144
C	9.34856	3.88123	3.87206
C	7.14103	3.36488	4.64150
C	8.98704	3.51356	2.57641
C	6.75836	3.02850	3.34612
C	7.68410	3.10087	2.30512
H	10.36951	4.22152	4.06433
H	6.41415	3.28029	5.44657
H	9.72508	3.56083	1.77167
H	5.73691	2.69345	3.14598
H	7.38966	2.82415	1.28922
C	10.04957	5.31013	6.48363
C	9.96196	6.49024	5.71991
C	11.18493	5.15241	7.29745
C	10.93896	7.47889	5.79074
C	12.17747	6.13153	7.36275
C	12.05232	7.30300	6.61689
H	9.08450	6.64156	5.08644
H	11.30152	4.23989	7.88886
H	10.83082	8.39343	5.20192
H	13.05175	5.97608	8.00046
H	12.82358	8.07588	6.67410

4. References

[1] N. Kuhn, T. Kratz, *Synthesis* **1993**, *1993*, 561-562.

[2] W. Malisch, R. Lankat, S. Schmitzer, R. Pikl, U. Posset, W. Kiefer, *Organometallics* **1995**, *14*, 5622-5627.

[3] G. Dübek, F. Hanusch, S. Inoue, *Inorg. Chem.* **2019**, *58*, 15700-15704.

[4] APEX suite of crystallographic software, APEX 3 version 2015.5-2; Bruker AXS Inc.: Madison, Wisconsin, USA, 2015

[5] SAINT, Version 7.56a and SADABS Version 2008/1; Bruker AXS Inc.: Madison, Wisconsin, USA, 2008.

[6] G.M. Sheldrick, SHELXL-2014, University of Göttingen, Göttingen, Germany, 2014.

[7] C.B Hübschle; G.M. Sheldrick; B.J. Dittrich, Appl. Cryst. 2011, 44, 1281-1284.

[8] G.M Sheldrick, SHELXL-97, University of Göttingen, Göttingen, Germany, 1998.

[9] A.J.C. Wilson, International Tables for Crystallography, Vol. C, Tables 6.1.1.4 (pp. 500-502), 4.2.6.8 (pp. 219-222), and 4.2.4.2 (pp. 193-199); Kluwer Academic Publishers: Dordrecht, The Netherlands, 1992.

[10] C.F. Macrae; I.J. Bruno; J.A. Chisholm;P.R. Edgington; P. McCabe; E. Pidcock; L. Rodriguez-Monge; R. Taylor; J. van de Streek;P.A.J. Wood, Appl. Cryst. 2008, 41, 466-470.

[11] a) F. Neese, *Wiley Interdiscip. Rev.: Comput. Mol. Sci.* **2018**, *8*, e1327; b) F. Neese, *Wiley Interdiscip. Rev.: Comput. Mol. Sci.* **2012**, *2*, 73-78.

[12] a) J. P. Perdew, M. Ernzerhof, K. Burke, *J. Chem. Phys.* **1996**, *105*, 9982-9985; b) C. Adamo, V. Barone, *J. Chem. Phys.* **1999**, *110*, 6158-6170.

[13] a) S. Grimme, S. Ehrlich, L. Goerigk, *J. Comput. Chem.* **2011**, *32*, 1456-1465; b) S. Grimme, J. Antony, S. Ehrlich, H. Krieg, *J. Chem. Phys.* **2010**, *132*, 154104.

[14] F. Weigend, R. Ahlrichs, *Phys. Chem. Chem. Phys.* **2005**, *7*, 3297-3305.

[15] a) D. Andrae, U. Häußermann, M. Dolg, H. Stoll, H. Preuß, *Theor. Chim. Acta* **1990**, *77*, 123-141; b) K. Eichkorn, F. Weigend, O. Treutler, R. Ahlrichs, *Theor. Chem. Acc.* **1997**, *97*, 119-124.

[16] F. Weigend, *Phys. Chem. Chem. Phys.* **2006**, *8*, 1057-1065.

[17] https://comp.chem.umn.edu/freqscale/190107_Database_of_Freq_Scale_Factors_v4.pdf. Version from January 7, 2018.

[18] a) E. v. Lenthe, E. J. Baerends, J. G. Snijders, *J. Chem. Phys.* **1993**, *99*, 4597-4610; b) C. van Wüllen, *J. Chem. Phys.* **1998**, *109*, 392-399.

[19] Kaupp, M.; Buhl, M.; Malkin, V. (Eds) Calculation of NMR and EPR Parameters. Theory and Applications. Wiley-VCH, 2004.

[20] C. J. Cramer, D. G. Truhlar, *Acc. Chem. Res.* **2008**, *41*, 760-768.

[21] G. Knizia, J. E. M. N. Klein, *Angew. Chem., Int. Ed.* **2015**, *54*, 5518-5522.

[22] Y. Zhao, D. G. Truhlar, *Theor Chem Acc* **2008**, *120*, 215-241.

[23] L. Goerigk, *J Phys Chem Lett* **2015**, *6*, 3891-3896.

[24] a) J. Tao, J. P. Perdew, V. N. Staroverov, G. E. Scuseria, *Phys. Rev. Lett.* **2003**, *91*, 146401; b) V. N. Staroverov, G. E. Scuseria, J. Tao, J. P. Perdew, *J. Chem. Phys.* **2003**, *119*, 12129-12137.
